# In-depth magnetometry and EPR analysis of the spin structure of human-liver ferritin: from DC to 9 GHz†

**DOI:** 10.1039/d3cp01358h

**Published:** 2023-10-09

**Authors:** Lucia Bossoni, Jacqueline A. Labra-Muñoz, Herre S. J. van der Zant, Vera Čaluković, Anton Lefering, Ramon Egli, Martina Huber

**Affiliations:** a C. J. Gorter Center for High Field MRI, Department of Radiology, Leiden University Medical Center The Netherlands; b Department of Physics, Huygens-Kamerlingh Onnes Laboratory, Leiden University 2300 RA Leiden The Netherlands huber@physics.leidenuniv.nl; c Kavli Institute of Nanoscience, Delft University of Technology 2628 CJ Delft The Netherlands; d RST-FAME, Delft University of Technology Delft The Netherlands; e GeoSphere Austria, Department of Geophysics Howe Warte 38 1190 Vienna Austria ramon.egli@geosphere.at

## Abstract

Ferritin, the major iron storage protein in organisms, stores iron in the form of iron oxyhydroxide most likely involving phosphorous as a constituent, the mineral form of which is not well understood. Therefore, the question of how the *ca.* 2000 iron atoms in the ferritin core are magnetically coupled is still largely open. The ferritin core, with a diameter of 5–8 nm, is encapsulated in a protein shell that also catalyzes the uptake of iron and protects the core from outside interactions. Neurodegenerative disease is associated with iron imbalance, generating specific interest in the magnetic properties of ferritin. Here we present 9 GHz continuous wave EPR and a comprehensive set of magnetometry techniques including isothermal remanent magnetization (IRM) and AC susceptibility to elucidate the magnetic properties of the core of human liver ferritin. For the analysis of the magnetometry data, a new microscopic model of the ferritin-core spin structure is derived, showing that magnetic moment is generated by surface-spin canting, rather than defects. The analysis explicitly includes the distribution of magnetic parameters, such as the distribution of the magnetic moment. This microscopic model explains some of the inconsistencies resulting from previous analysis approaches. The main findings are a mean magnetic moment of 337*μ*_B_ with a standard deviation of 0.947*μ*_B_. In contrast to previous reports, only a relatively small contribution of paramagnetic and ferrimagnetic phases is found, in the order of maximally 3%. For EPR, the over 30 mT wide signal of the ferritin core is analyzed using the model of the giant spin system [Fittipaldi *et al.*, *Phys. Chem. Chem. Phys.*, 2016, **18**, 3591–3597]. Two components are needed minimally, and the broadening of these components suggests a broad distribution of the magnetic resonance parameters, the zero-field splitting, *D*, and the spin quantum number, *S*. We compare parameters from EPR and magnetometry and find that EPR is particularly sensitive to the surface spins of the core, revealing the potential to use EPR as a diagnostic for surface-spin disorder.

## Abstract 2

Electron paramagnetic resonance and SQUID magnetometry reveal the magnetic properties of the iron-rich ferritin core. A new theory links the spin state of ∼2000 Fe atoms in the core to surface-spin canting.

## Graphical abstract

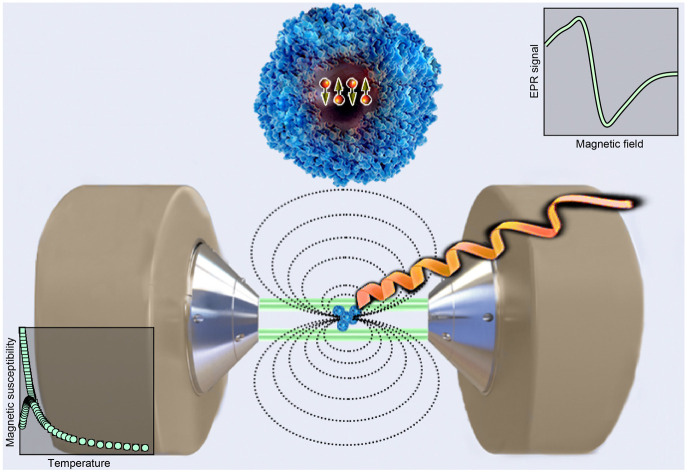

## 1 Introduction

Ferritin, has fascinated scientists for decades. This ubiquitous iron-storage protein is made of a protein shell enclosing a core of bioavailable iron mineral.^1^ The mammalian apoferritin shell contains two distinct polypeptide subunits: a heavy (21 kDa) and a light (19 kDa) chain.^2^ These self-assemble into a 24-mer spherical structure which, depending on the organism and the specific organ it is found in, can have different heavy- *vs.* light-chain ratios. While the heavy chain has a ferroxidase activity and protects cells from redox-active iron by rapid uptake of Fe(ii) and catalytic oxidation to Fe(iii), the light chain promotes the nucleation and storage of iron as a biomineral.^3^ The inner and outer dimensions of the protein shell are ∼7–8 nm and ∼12 nm, respectively. The biomineral inside the ferritin hollow cavity has received the attention of the biomedical community, because of a link between the protective function of ferritin against cellular iron toxicity^4^ and altered core composition in the brain of patients with neurodegenerative diseases.^5,6^ From a physics standpoint, ferritin is also relevant in the fundamental study of nanoparticle properties. Through the biochemical machinery of iron incorporation and the protective protein shell, the composition, spin configuration, and size of the core are well-controlled and protected from post-assembly modification, factors that sometimes are difficult to control in man-made iron nanoparticles. Furthermore, the apoferritin shell prevents contact between the cores, eliminating magnetic exchange interactions.

Here, we focus on the magnetic properties of ferritin, which are an indicator for the spin structure and composition of the ferritin core. The magnetic properties of ferritin nanoparticles, for example their saturation magnetization, are directly linked to the relaxation rates of ferritin-rich tissue. As such, the magnetism of the protein influences the contrast of R2 and R2*-weighted MRI images.^7–9^

In the past decades, ‘bulk’ magnetometry techniques have been used to characterize the magnetic and mineral state of ferritin,^10,11^ along with spectroscopy techniques such as Mössbauer spectroscopy,^11,12^ electron paramagnetic resonance (EPR),^13,14^ nuclear magnetic resonance (NMR),^15–17^ as well as electron and X-ray microscopy techniques,^18,19^ and diamond-based quantum spin relaxometry to study the ferritin room temperature magnetic properties.^20^ Electron paramagnetic resonance (EPR), sometimes also referred to by the more general term electron magnetic resonance (EMR), has also been applied to ferritin,^13,14,21–25^ in spite of intrinsic challenges related to extreme spectral broadening.

It is generally agreed upon that the ferritin core is predominantly composed of a mineral resembling ferrihydrite,^26^ a poorly crystalline ferric oxyhydroxide. While some studies suggested a multiphase core composition,^6,27,28^ this hypothesis is not supported by NMR,^16^ magnetooptical measurements,^17^ electron energy-loss spectroscopy,^29^ and only partially by muon spin rotation. On the other hand, there is a general consensus on the following properties: (1) iron-spins in the cores are antiferromagnetically (AF) coupled, (2) the cores possess a spontaneous magnetic moment of the order of ∼300*μ*_B_, (3) the magnetic moments becomes blocked below *T*_b_ ≈ 12 K over the typical time span of magnetometric measurements, and (4) ferritin is superparamagnetic above *T*_b_.^13,14,21,24^ However, several questions remain still unanswered: for instance, the origin of the spontaneous magnetic moment of the cores has been generically attributed to randomly distributed defects in the AF lattice,^10,30,31^ but the nature and location of these defects (*e.g.*, in the bulk or at the surface) remain ambiguous.^32–34^ The magnetic moment might also arise from core alteration, as it has been postulated in the case of ferrihydrite, which becomes partially ferrimagnetic during its transformation to hematite.^35^ The maghemite-like ferrimagnetic phase resulting from this alteration process might explain the postulated low-coercivity phase in ferritin.^27^ The apparent multiphase nature of ferritin might also originate from the interaction between different units within the core,^26,36^ or between core and surface spins.^13^ Such interactions can explain spin glass-like signatures such as shifted field-cooled hysteresis loops.^37^ Finally, the absence of a spin-flop transition in fields up to 50 T,^38^ which is incompatible with the reported exchange and anisotropy fields of ferritin, questions the definition of anisotropy energy and energy barriers in AF nanoparticles.^39^

Several limitations concur to our presently incomplete picture of the magnetic properties of ferritin. Interpretations of magnetometry and spectral techniques are intrinsically non-unique, therefore relying on models that require some a-priori knowledge of the spin structure of ferritin cores. Furthermore, most if not all magnetic parameters of ferritin are broadly distributed.^40^ This can lead to erroneous conclusions if such broad distributions are replaced with mean values without considering possible correlations. Furthermore, broad parameter distributions make model fits very sensitive to initial assumptions and measurement noise, as seen with the multiple approaches used to model the superparamagnetic and linear contributions to in-field magnetization curves.^10,30,32,39,41^ Finally, magnetometric and spectral techniques have been rarely combined,^34^ despite the intrinsic advantages of using complemental information to better constrain existing models.

In this work, we focus on the EPR and magnetometric properties of human-liver ferritin (HuLiFt) and address some of the issues mentioned above. Using these techniques, we explore the spin dynamics over a broad frequency range that includes DC (magnetization) to sub-kHz (AC-susceptibility) and microwave (9 GHz, EPR) measurements. The present work not only combines different methods, but also different fields of research, such as magnetism and, due to the mineral core of ferritin and the widespread occurrence of ferrihydrite in nature, geological aspects. We unified different naming conventions used for the characterization of magnetic materials using a single symbol for each quantity, except the magnetic moment, for which *m* is used in the case of magnetometric measurements and *μ* in the EPR context (see the list of symbols given in the ESI†).

Most EPR studies have been performed at 9 GHz on horse-spleen ferritin.^13,14,21,23,24^ The broad superparamagnetic signal located at *g*′ = 2 was associated with antiferromagnetically coupled Fe(iii) ions in the ferritin cores, while a weak signal near *g*′ = 4.3 was attributed to a small number of mononuclear Fe(iii) centers showing typical paramagnetic behaviour.^13,21,23^ A few EPR studies have been performed on human ferritin, specifically from human spleens,^14,24^ proposing that the broad EPR signal results from two overlapping broad contributions, a very anisotropic one at lower fields, and an isotropic one around *g*′ = 2. Interestingly, in ref. 24 the 35 GHz data show the lower field EPR component of horse-spleen ferritin has a much lower intensity than its counterpart in human-spleen ferritin, however, the paper does not comment on this difference.

The EPR lineshape of superparamagnetic nanoparticles have been addressed using qualitative descriptions, such as the one by Noginova *et al.*^42^ based on surface quantum effects, according to which the EPR intensity is proportional to exp(−*μB*/*k*_B_*T*). Previously, Usselman *et al.*^43^ used two different models to simulate temperature-dependent lineshape trends of iron oxide nanoparticles mineralized in *Listeria innocua* protein cages. The first model^44,45^ provides a qualitative description of the lineshape dependence on temperature^46^ and frequency,^47^ assuming an ensemble of non-interacting single-domain particles, whose magnetization dynamics is described by the Landau–Lifshitz equation.^45^ The second model^48^ calculates the moment-distribution function by considering superparamagnetic fluctuations and ensemble broadening due to a distribution of anisotropy axes.^48^ Other models include an improved static model to describe the decrease in the magnetic anisotropy as temperature increases.^49^ However, these models do not reproduce all features of the measured EPR spectra.

Here, we use a quantum mechanical model^50,51^ to analyze 9 GHz EPR spectra of ferritin in solution, rather than in the freeze dried state, to exclude ferritin–ferritin interactions. Our analysis reveals multiple EPR spectral components resulting from the distributed nature of moment and anisotropy field distributions.

Magnetic simulations of equilibrium magnetization curves are used to understand the origin of the magnetic moment of ferritin cores. The theory behind these simulations is described in Section 3 and its application in Sections 4.2–4.5. Using these simulations and the measurement of isothermal remanent magnetization, we developed a new approach for the determination of the anisotropy field, magnetic moment, and blocking temperature distributions, as well as the relations existing between these parameters and between magnetometric and EPR measurements.

Our results can be explained by a simple model of AF nanoparticles whose magnetic moment is generated by surface-spin canting, rather than defects. This model explains the lack of a spin-flop transition below ∼50 T. We also observe minor (<3%) contributions to the anisotropy field distribution that are compatible with a ferrimagnetic phase and a phase with very large anisotropy, respectively. Finally, we show that EPR is particularly sensitive to surface spins.

## 2 Materials and methods

### 2.1 Properties and characterization of the human-liver ferritin

**Fig. 1:**
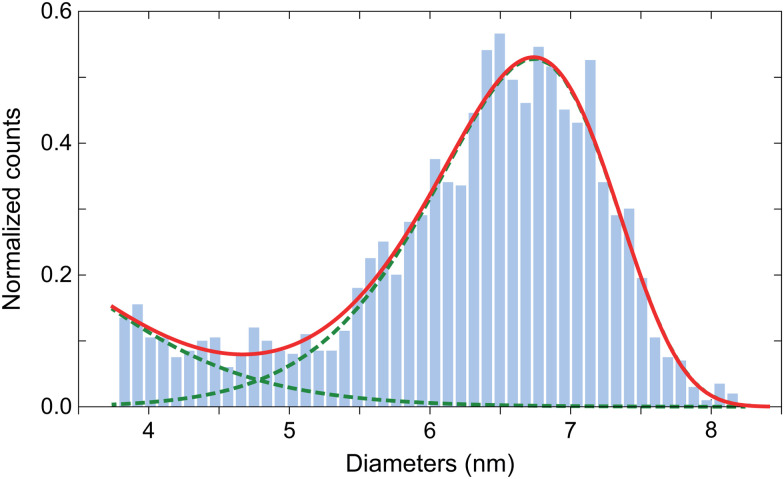
Distribution of human ferritin core size and fit to an empirical function given by the sum (solid line) of a lognormal distribution with parameters *μ* = 1.128 and *σ* = 0.2704, and a Weibull distribution with parameters *β* = 6.797 and *η* = 11.08 (dashed lines).

Commercial human-liver ferritin (HuLiFt) was obtained from LEE Biosolutions (Cat. No. 270-40, Lot 08E1805) and used without further purification. The protein concentration was 3.6 mg ml^−1^ with >95% purity as assessed by SDS-PAGE, Coomassie blue and Prussian blue stains (see ESI† for details). The protein loading factor was determined by inductively coupled plasma mass spectrometry (ICP-MS), yielding 1967 ± 78 iron atoms per ferritin. The size distribution of ferritin cores was obtained from transmission electron microscopy (TEM) images (Fig. S1, ESI†), using automatic circle detection based on the MATLAB function *regionprops*. The analysis of 2100 particles showed that core sizes strongly deviate from a unimodal lognormal-like distribution (Fig. 1). The main peak of the core size distribution is well approximated by a Weibull distribution with a mean of 6.5 nm and a median of 6.6 nm. An excess of small sizes with respect to the Weibull fit is observed below 4.5 nm and might represent incomplete fragmented cores.

### 2.2 Magnetometry

The ferritin solution as purchased, *i.e.* without adding glycerol, see materials, was immersed into liquid nitrogen and subsequently freeze-dried over ∼48 hours. For further details and sample handling see also [ref. 52]. The obtained powder sample was pressed into a gel capsule and loaded into a Quantum Design MPMS-XL SQUID magnetometer mounting the reciprocating sample option (RSO, noise floor: 1 pA m^2^). First, the magnetic moment in a 5 mT field was measured as a function of temperature after cooling to 5 K in zero field (zero-field-cooled, ZFC) and in 5 mT (field-cooled, FC), respectively. Then, the field-induced magnetization was measured at 5 K and 150 K (complete hysteresis loops) and in the 5–250 K range (initial magnetization curves). Low-field AC susceptibility was measured after ZFC to 20 K in a 5 mT DC field with superimposed longitudinal AC field of 0.38 mT amplitude and frequencies *ν* = 0.113, 0.669, 4.481, 29.99, 59.9 Hz. Only the in-phase AC susceptibility was processed, as the quadrature component was too noisy. ZFC and FC hysteresis loops were measured at 5 K and 25 K in order to detect the presence of an exchange field. Finally, high-resolution isothermal remanent magnetization (IRM) curves were acquired from 0 to 5 T in steps comprised between 1 and 200 mT, at 3, 5, 9, 11, 13, 17, and 20 K. Each point *M*_r_(*B*) of a IRM curve is obtained by ramping the field from 0 to *B* and then back to 0, with no overshoot. Repeated measurements were acquired to ensure reproducibility and enhance the signal-to-noise ratio (SNR): a SNR ≥100 is ideally needed over the field range containing relevant coercivity contributions. All measurements are expressed as mass magnetization obtained by dividing the magnetic moment by the sample mass. All data analyses were carried out in Matlab2016a and Mathematica 12 using built-in non-linear minimization routines.

### 2.3 Electron paramagnetic resonance

In order to avoid ferritin-ferritin interactions that might occur in the freeze-dried samples^14,21,23^ we used the buffered ferritin solution for EPR measurements. For this purpose, 100 μL of the ferritin solution with 20% glycerol (vol/vol) solution were transferred into a 4 mm outer diameter EPR tube. The tube was then immediately frozen in liquid nitrogen. Continuous wave (CW) EPR measurements were performed with a 9 GHz ELEXSYS E680 EPR spectrometer (Bruker, Rheinstetten, Germany), equipped with a rectangular cavity. The spectra were recorded with 20 mW power, 90 kHz modulation frequency, and 29.46 *G*_pp_ field modulation amplitude at temperatures comprised between 5 and 210 K. The accumulation time was 11.2 min per spectrum. A helium flux cryostat was used to control the temperature. Temperatures were taken from the readout of the Oxford temperature unit connected to a thermocouple placed beneath the sample. Measurement of the buffered suspension instead of freeze-dried powders produces a lower EPR signal intensity; however, signal quality was still sufficient for further processing.

Simulations of measured spectra have been conducted with the EasySpin package (5.2.4) using a Matlab (R2019a) script. Spectra between 5 and 15 K were not analyzed because it was not possible to accurately discriminate the broad signal from background noise. Final simulations have been performed with two components. The parameters *D* and *H*_strain_ (Gaussian broadening) were adjusted independently for each component and each temperature, along with the relative contributions of the two components and the pepper routine parameters of the EasySin package, after choosing *S* = 10 and *g* = 2.01 as fixed parameters for both components. The sensitivity of the model to the parameters *D* and *H*_strain_ was tested by changing, for example, *D* of one component and leaving all other parameters unchanged, until a visible lineshape alteration was detected (see section “Sensitivity of EPR parameters” in the ESI†).

## 3 Theoretical background of magnetometry analysis

In the following, we discuss different models of the spin structure of ferritin cores and the implications they have on magnetometry and EPR results.

Starting from models proposed in the literature and our measurements we derive a new model for the spin structure of ferritin. First, we analyze the implications of polyphase ferritin cores for the interpretation of magnetometry results, showing that individual phases coexisting in the same core cannot be discriminated by isothermal magnetic measurements. Next, we use these findings to discuss possible spin structures that are compatible with equilibrium magnetization curves, showing that spin canting is needed to explain the ferritin-core susceptibility at lower field and the lack of a spin-flop transition. The universal relation between mean magnetic moment and number of Fe atoms in ferritin and ferrihydrite nanoparticles further confirms these findings. Finally, magnetometric parameters are discussed in relation to the energy barrier that needs to be overcome to switch the magnetic moment of ferritin cores, showing that the expression *E*_b_ = *KV* is valid also in the case of AF nanoparticles, and that a wide distribution of anisotropy fields can be a consequence of the fact that *B*_a_, the anisotropy field, is inversely proportional to *m*, the magnetic moment.

### 3.1 Magnetic phases proposed for ferritin in the literature

The use of TEM X-ray Adsorption Near Edge Spectroscopy (XANES) and Small-Angle X-ray Scattering (SAXS) for probing the composition of ferritin cores (*i.e.*, the proportion of Fe and O atoms), and of Electron Energy-Loss Spectroscopy (EELS) for probing the oxidation state of Fe ions suggests a polyphase structure^6,27,28^ made mainly of ferrihydrite (Fh) or a phosphorous-rich phase whose structure is similar to that of ferrihydrite,^17^ with minor hematite (α-Fe_2_O_3_), magnetite (Fe_3_O_4_), and wüstite (FeO) contributions.^28^ In the human brain, these secondary contributions tend to increase with age and in patients with neurological diseases.^6^ Spatial EELS analyses suggest that magnetite is concentrated at the core surface.^28^ On the other hand, other studies based on magnetooptical measurements suggest a single-phase core structure.

The hypothetical polyphase nature of ferritin cores might be the result of Fh alteration or variable iron storage mechanisms. Fh is known to form an ordered ferrimagnetic structure during aging,^35^ as intermediate product on the pathway to full conversion to hematite. The conversion rate of synthetic Fh is very slow at room temperature, but it is greatly enhanced in the presence of ligands.^53^ Heating during sample preparation might therefore be an issue for the assessment of ferritin core composition. On the other hand, a 3D morphology study suggests that ferritin cores are composed of up to eight regions with disordered surfaces, consistent with the eight channels in the protein shell that deliver iron to the central cavity.^26^ Disordered surfaces are magnetically distinct from the bulk and can therefore be considered as an additional phase.

Ferritin-core phases identified so far are characterized by different forms of magnetic order, including AF (Fh, FeO), canted AF (hematite), ferrimagnetic (magnetite), and speromagnetic (surfaces). However, if these phases coexist within the same core units, exchange coupling is expected to produce a collective spin behavior that is not equivalent to the superposition of bulk-phase properties. Magnetometry data might still suggest a polyphase composition, for instance through a bimodal magnetic moment or energy-barrier distribution.^27,36^ In our case, the existence of multiple magnetic phases is supported by IRM acquisition curves (see Sections 4 and 5).

Magnetic evidences used so far in support of significant contributions from phases other than Fh depend heavily on the way energy barrier and magnetic moment distributions are measured and modelled. For instance, the energy barrier distribution obtained from magnetic viscosity measurements is bimodal,^36^ while the same distribution derived from quadrature AC susceptibility data is strictly unimodal.^54^ The case of the magnetic moment distribution is even more ambiguous, as the fit of equilibrium magnetization curves with two superparamagnetic components with distinct single-valued magnetic moments, as proposed by Brem *et al.*,^27^ is a valid alternative to the distributed moment model described in the Results section. Evidently, the additional degree of freedom of the two-component model enables better fits to the data. Nevertheless, as we demonstrate further below, this interpretation is incompatible with the IRM results obtained in the present study and therefore discarded.

### 3.2 The magnetic signature of idealized spin structures

Consider a collinear two-sublattice AF particle with sublattice magnetization *M*_0_ and exchange constants *A*_*a*_, *A*_*b*_, and *A*_*ab*_. In the case of slightly uncompensated sublattices, the two sublattice magnetizations are given by *M*_*b*_ = *M*_0_ and *M*_*a*_ = (1 + *α*)*M*_0_, respectively, where *α* > −1 is the fraction of excess moment in one sublattice. This creates a net spontaneous magnetic moment *m*_uc_ = *αM*_0_*V* in a particle with volume *V*. In the following, it is assumed that *m*_uc_ is rigidly coupled to the sublattice magnetizations, due to the strong AF coupling,^55,56^ so that any change of the magnetic moment is produced by the uniform rotation of all spins in both sublattices. Furthermore, particles possess a positive uniaxial magnetic anisotropy^57^ with anisotropy constant *K* and easy axis parallel to the unit vector **e** = (sin *ϕ*,0,cos *ϕ*), so that 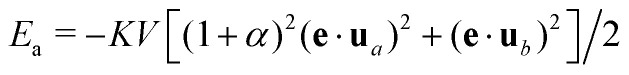 is the anisotropy energy corresponding to sublattice magnetizations parallel to the unit vectors **u**_*a*_ and **u**_*b*_, respectively.^58^ In the absence of external fields, the total energy is minimized when the sublattice magnetizations are exactly antiparallel and oriented along the easy axis. The application of a field **B** rotates the lattice magnetizations away from the easy axis and introduces an induced spin canting (Fig. S23, ESI†). Following Bogdanov *et al.*,^58^ we define the spin canting angle −π/2 ≤ *ε* ≤ π/2, such that **u**_*a*,*b*_ = ±**p** cos *ε* + **n** sin *ε*, where *p* is the so-called Néel unit vector parallel to the staggered magnetization direction **u**_*a*_ − **u**_*b*_, and **n**⊥**p** is the unit vector parallel to the canting magnetization direction **u**_*a*_ + **u**_*b*_. In a spherical coordinate system with **B**‖**ẑ**, **p** = (sin *θ* cos *ψ*,sin *θ* sin *ψ*,cos *θ*) and **n** = **n**_1_ cos *λ* + **n**_2_ sin *λ* with **n**_1_ = (**ẑ** × **p**) × **p** parallel to the plane spanned by **B** and **e**, **n**_2_ = **ẑ** × **p**⊥**z**, 0 ≤ ***θ*** ≤ π, and −π ≤ *ψ*, *λ* ≤ π. In this case, the total energy *E* per unit of volume of a particle with the above properties is given by1
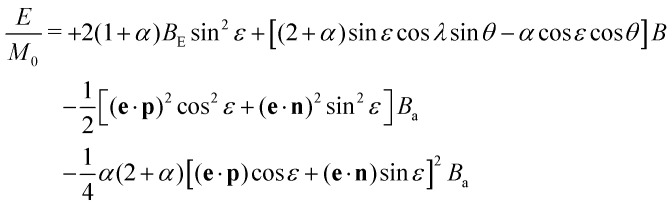
with the exchange field *B*_E_ = *A*_*ab*_*M*_0_ and the anisotropy field *B*_a_ = 2*K*/*M*_0_. The equilibrium magnetization of an ensemble of non-interacting particles is given by^59^2
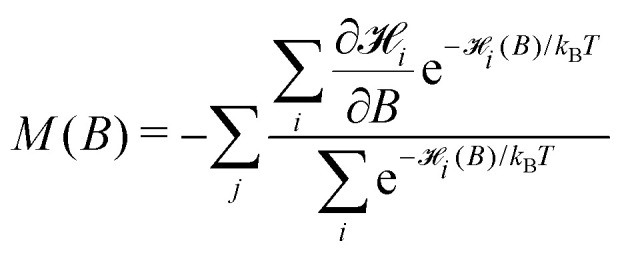
for all particles with easy axis orientations **e**_*j*_ and states *i* of their Hamiltonian ℋ. In the classical case where the magnetic moments can take any orientation, ℋ = *E*(*θ*,*ψ*,*λ*,*ε*) and ∂_*i*_ℋ/∂*B* = −*M*_0_*Vζ*, with

3 *ζ* = *α* cos *ε* cos *θ* − (2 + *α*)sin *ε* sin *θ* cos *λ*

being the ratio between the component of the magnetic moment parallel to *B*, and *M*_0_*V*. In this case, summations in eqn (2) are replaced by integrals, obtaining4
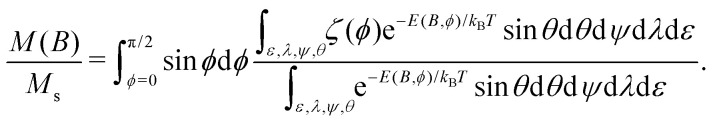
This result cannot be further simplified, since the dependence of *E* on all five integration variables is not separable, except when spin canting and anisotropy are negligible: in this case eqn (4) converges to the well-known Langevin law of superparamagnetism.^59^

**Fig. 2:**
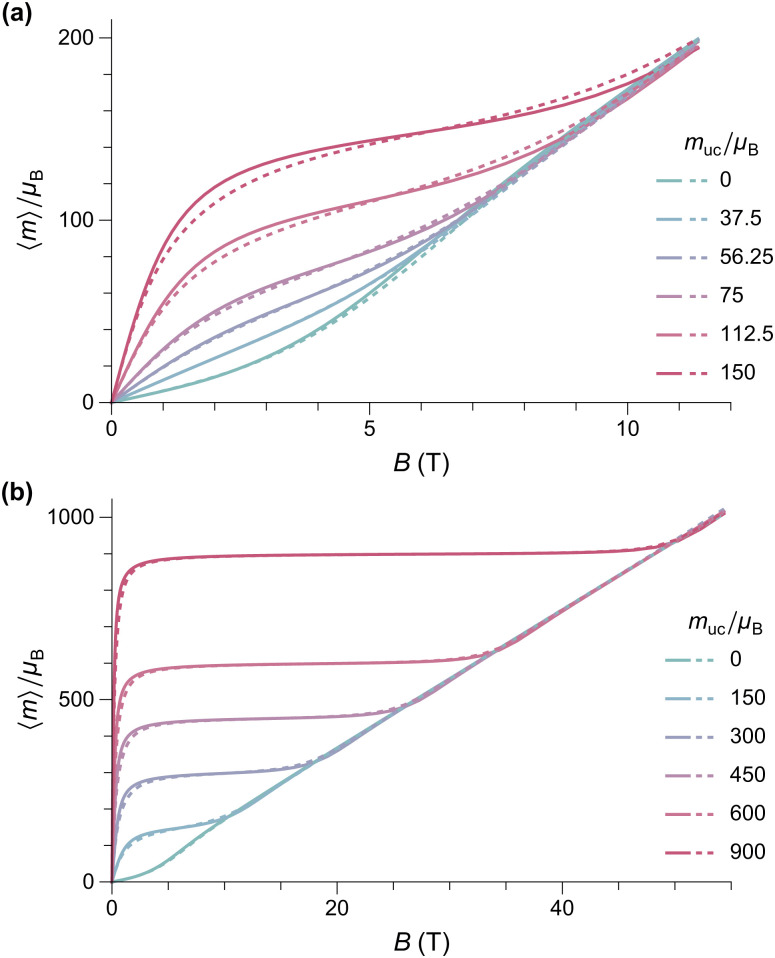
Numerical simulations of the *T* = 50 K equilibrium magnetization of randomly oriented AF particles with no anisotropy (*K* = 0, solid lines), and with anisotropy (*K* = 18.3 kJ m^−3^, dashed lines), for selected values of *m*_uc_. Other model parameters are *M*_0_ = 366.8 kA m^−1^, *B*_E_ = 320 T, and *M*_0_*V* = 6000*μ*_B_. (a) and (b) Represent the same simulations over different field ranges.

Numerical evaluations of eqn (4) are extremely time consuming, due to the five-fold integrals: on a PC, a single equilibrium magnetization calculation takes ∼7 min using an optimized method (ESI:† Equilibrium magnetization models). Simulations of *M*(*B*) at 50 K using *B*_E_ ≈ 320 T for ferritin,^56^ show that the equilibrium magnetization is governed by two regimes (Fig. 2). In small fields, thermal fluctuations, which act on each degree of freedom, induce a small spin-canting angle that adds a random canting moment *m*_c_ perpendicular to the uncompensated moment *m*_uc_. If *m*_uc_ = 0, the random canting moment generates a low-field susceptibility *χ*_lf_ = *χ*_⊥_/3, where *χ*_⊥_ = *M*_0_/*B*_E_ is the bulk perpendicular susceptibility of the AF lattice. In larger fields, the canting moment gets progressively aligned with the field, producing a transition to the high-field regime given by *M* = *χ*_⊥_*B* (Fig. 2b). If *αm*_nc_*B*_E_ ≫ *k*_B_*T*, the uncompensated moment is much larger than the canting moment, and the low-field magnetization converges to the Langevin model prediction, with *χ*_lf_ = *M*_s_*m*_uc_/*k*_B_*T*, regardless of single particle anisotropy. At larger fields, anisotropy decreases the equilibrium magnetization, because of the competition between field and easy axis alignment (dashed lines, Fig. 2a). The same result has been obtained previously^59,60^ for non-interacting ferromagnetic particles. In the simulations of Fig. 2, this ferromagnetic-like regime holds for *m*_uc_ ≥ 150*μ*_B_ and *B* < 5 T.

**Fig. 3:**
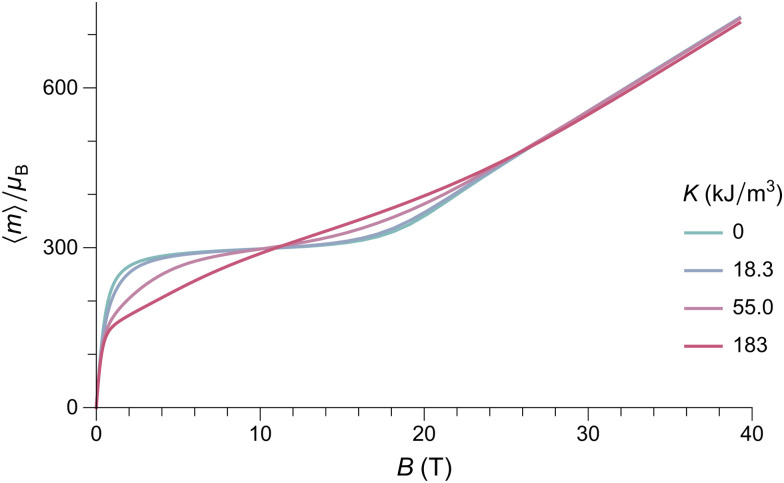
Numerical simulations of the *T* = 50 K equilibrium magnetization of randomly oriented AF particles with *m*_uc_ = 300*μ*_B_ and selected values of the anisotropy constant. Other model parameters are the same as in Fig. 2.

External fields increase the canting angle of all particles whose Néel vector **p** is not parallel to the field direction; however, at the same time, the uncompensated moment tends to align the Néel vector with the field, so that spin canting becomes less effective. As a result, the spin canting angle continues to be controlled only by thermal fluctuations until the alignment of the canting moment becomes energetically more favorable than that of *m*_uc_: at this point, a so-called spin-flop transition takes place through rotation of the Néel vector by 90°. In bulk antiferromagnets, this is a sudden event that occurs at the spin-flop field *B*_sf_ ≈ (2*B*_E_*B*_a_)^1/2^ predicted by mean-field theory.^58^ This result does not hold for the equilibrium magnetization of AF nanoparticles, because thermally activated spin canting occurs in all fields. As shown by our simulations, the spin-flop field is defined, in this case, by the intersection of the Langevin law with the high-field regime *M* = *χ*_⊥_*B* of perfect antiferromagnets (Fig. 2b). For sufficiently large values of *α* (*e.g.*, *α* ≈ 0.025 for the 50 K simulations of Fig. 2), the spin-flop field for the equilibrium magnetization is then given by *B*_sfe_ = *αB*_E_. Contrary to bulk antiferromagnetism,^39^ magnetic anisotropy does not affect *B*_sfe_; instead, it increases the slope of *M*(*B*) in the Langevin saturation regime, until it becomes indistinguishable from the spin-flop regime (Fig. 3). This occurs because the additional Néel vector misalignment produced by randomly oriented anisotropy axes enhances the induced spin canting and its contribution to the equilibrium magnetization already in fields <*B*_sfe_. *Vice versa*, the same anisotropy effect reduces the alignment of the canting moment above *B*_sfe_.

**Fig. 4:**
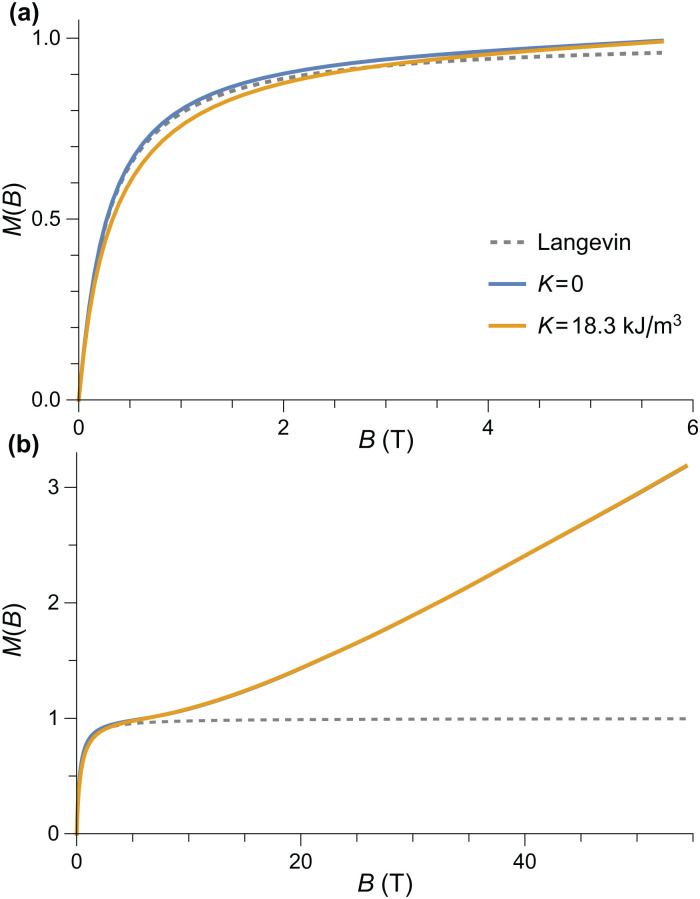
Numerical simulations of the *T* = 50 K equilibrium magnetization of randomly oriented AF particles with the lognormal distribution of magnetic moments deduced from the modified Langevin fit of *M*(*B*) measurements (logarithmic mean: 215*μ*_B_, logarithmic standard deviation: 0.963), calculated using the Langevin model (dashed line), and the AF model of Fig. 2 (solid lines). (a) and (b) Represent the same simulations over different field ranges.

The defect moment model illustrated above is our starting point for assessing the validity of the modified Langevin fit we used to estimate the distribution of *m*_uc_ from *M*(*B*) measurements, and for testing the origin of the uncompensated moment in ferritin cores. For this purpose, we used eqn (4) to calculate *M*(*B*) at 50 K for an ensemble of randomly oriented particles with the same lognormal distribution of *m*_uc_ obtained from the modified Langevin fit, together with model parameters representative for ferritin, that is, *K* = 18.3 kJ m^−3^ (Section 5.2), *B*_E_ = 320 T, and *M*_0_*V* = 6000*μ*_B_ (Fig. 4b). According to this simulation, the spin-flop transition is expected to occur at ∼10 T, instead of (2*B*_E_*B*_a_)^1/2^ ≈ 36 T. This is beyond the maximum field used in our *M*(*B*) measurements, but well below the ∼50 T maximum field used in experiments that failed to detect such a transition.^38,39^ The lack of a spin-flop transition below 50 T has been attributed to larger-than-expected values of *B*_E_ and/or *B*_a_.^38,39^ As shown by our simulations, *B*_sfe_ is not affected by single particle anisotropy, while the >5 times larger exchange field required to push *B*_sfe_ beyond the maximum field range of available measurements does not comply with *B*_E_ values obtained from high-field estimates of the AF susceptibility.^39^ Therefore, the only plausible explanation for the discrepancy between the simulation of Fig. 4 and actual high-field measurements of *M*(*B*) is that the defect model of Néel^61^ does not provide a correct description of the in-field magnetic moment of ferritin cores.

As far as the effect of anisotropy on the shape of *M*(*B*) is concerned, a relatively small but non-negligible reduction of the equilibrium magnetization occurs over the 0.5–3 T field range, before the onset of saturation (Fig. 4a). This alters the magnetic moment distribution obtained by the modified Langevin fit, lowering the apparent mean moment by ∼40% and increasing the apparent moment distribution width by ∼30%. A-posteriori corrections of the Langevin fit according to these results, however, would not be meaningful, since the absence of a spin-flop transition in fields ≤50 T requires a different model for the superparamagnetic behavior of ferritin. Therefore, we look for spin configurations that produce a net spontaneous moment that is not parallel to the Néel vector. A possible source for such spin configurations is surface anisotropy, because it affects the orientation of surface spins with respect to the bulk.

Surface spins are often assumed to be in a disordered, spin-glass-like state created by a distribution of exchange field vectors pointing to different directions.^62,63^ The exchange interaction between surface and internal spins manifests itself through exchange bias, that is, the horizontal shift of FC hysteresis. The existence of this exchange bias in horse-spleen ferritin^10^ and in our sample (see Section 4), along with data from dynamic Mössbauer spectroscopy,^34^ testifies for the existence of a surface spin layer in ferritin cores. Due to the sensitivity of exchange interactions to the position of ions, surface spins can take multiple configurations that are not necessarily associated to a complete disorder, as seen for instance with the spike, throttled, and two-pole configurations obtained from simulations of ferrimagnetic nanoparticles.^64^ These configurations decrease the net moment of particles with ferrimagnetic order, but represent a possible source of spontaneous moments in AF nanoparticles. Furthermore, the exchange coupling between surface and internal spins can alter the AF ordering of the whole particle.^65^ Recent simulations of small AF nanoparticles demonstrate this effect, with spike, throttled, and disordered internal spin configurations, as well as spin canting (Fig. 11 and 12 in Laura-Ccahuana and De Biasi^66^). Most importantly, these simulations show that surface anisotropy increases the spin-flop field and/or limits spin flopping to subregions of the particles or suppresses it completely, so that the bulk magnetization does no longer show the effects of a spin-flop transition.^66^ Similar effects might also occur at interfaces between different phases in a polyphase model of ferritin cores, especially if secondary phases consist of few surface atomic layers.^28^ Specific sources of spin canting moment include topological chiral magnetism induced by the Dzyaloshinskii–Moriya exchange interaction on surfaces and interfaces.^67^

In principle, the equilibrium magnetization of AF particles with surface anisotropy can be calculated by evaluating eqn (2) with the Hamiltonian^64,66^5
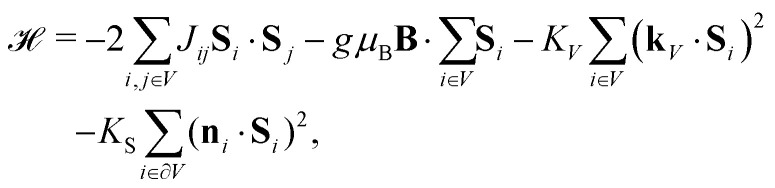
where **S**_*i*_ are the spin vectors, *J* the exchange constant, *K*_*V*_ the uniaxial volume anisotropy with easy axis **k**_*V*_, *K*_S_ the surface anisotropy, and *n*_*i*_ the surface normal vector for the *i*-th surface spin. This requires sampling the whole parameter space spanned by the spin vectors. Some characteristics of the complete solution can be captured by simulations based on an equivalent homogeneous system where the spontaneous moment *m*_c_ = 2*M*_0_*V* sin *ε*_s_ is produced by a zero-field canting angle *ε*_s_ (ESI:† Spontaneous spin canting).

**Fig. 5:**
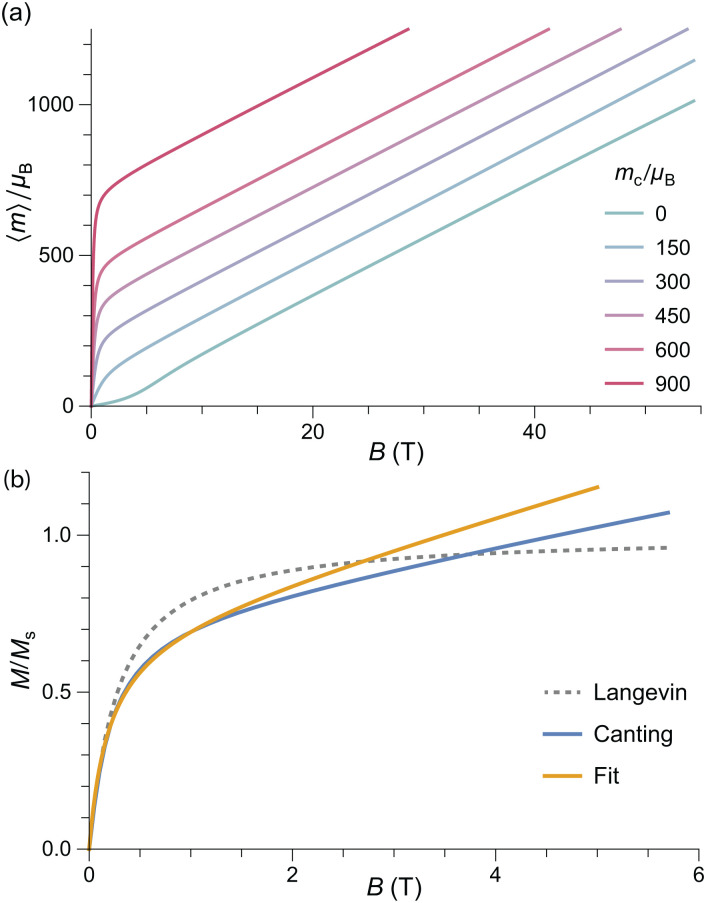
(a) Numerical simulations of the *T* = 50 K equilibrium magnetization of randomly oriented AF particles with no anisotropy for selected values of the canting moment *m*_c_. Other model parameters are *M*_0_ = 366.8 kA m^−1^, *B*_E_ = 320 T, and *M*_0_*V* = 6000*μ*_B_. (b) Same as (a) for a lognormal distribution of magnetic moments with logarithmic mean of 215*μ*_B_ and logarithmic standard deviation of 0.963. The Langevin model and the sum of the Langevin and linear terms of the measurement fits are shown for comparison.

As expected, the equilibrium magnetization of AF particles with a spontaneous canting moment is equivalent to the sum of a Langevin term that describes the superparamagnetism of *m*_c_, and a linear term *M* = *χ*_⊥_*B* that accounts for the induced spin canting (Fig. 5a). Because *m*_c_ is already perpendicular to the Néel vector, there is no spin-flop transition. The *M*(*B*) curve resulting from the same distribution of moments used to simulate uncompensated moment now contains a linear term *M* = *χB* comparable with the non-paramagnetic component ℒ(*mB*/*k*_B_*T*) + *χB* of the model used to fit *M*(*B*) data (Fig. 5b). The normalized slope *χ*/*M*_s_ of the linear term is ∼40% smaller than the fitted value at 50 K. A good agreement, on the other hand, is obtained at 250 K. The temperature-dependent mismatch is due to the fact that this model does not account for the effects of single-particle anisotropy, and in particular surface anisotropy, on *χ*.

### 3.3 The role of distributed parameters

Magnetic properties of ferritin are usually expressed in terms of averaged quantities or treated as single-valued parameters (*e.g.*, *K*, *B*_a_, the blocking temperature *T*_b_). This approach is correct only when it is applied to intrinsic properties of the material, such as the sublattice magnetizations and exchange constants, and the bulk magnetocrystalline anisotropy. Parameters that do not represent intrinsic properties of an AF crystal, such as the magnetic moment and the anisotropy field, must be treated as statistical distributions to avoid incorrect interpretations, as shown by the following example. The mean ferritin magnetic moment of ∼350*μ*_B_ obtained from simple^10,68^ or distributed (this work) Langevin fits is usually attributed to an uncompensated moment matching one of the three models proposed by Néel.^61^ The first Néel model associates *m*_uc_ with defects randomly distributed among the AF sublattices: in this case, *m*_uc_ ≈ *μ*_Fe_(*cN*)^1/2^, where *N* ≈ 2500 is the total number of Fe ions in ferritin, *μ*_Fe_ ∼ 5*μ*_B_ their magnetic moment, and *c* ≪ 1 the concentration of defects. The good match between 〈*m*〉 estimates obtained with *c* = 1 and with the Langevin fit has often been used as a validation of this model,^10,30,31^ even though a rigorous probabilistic analysis shows the largest mean moment 〈*m*_uc_〉_max_ ≈ 0.56*μ*_Fe_*N*^1/2^ is obtained with *c* = 0.5.

**Fig. 6:**
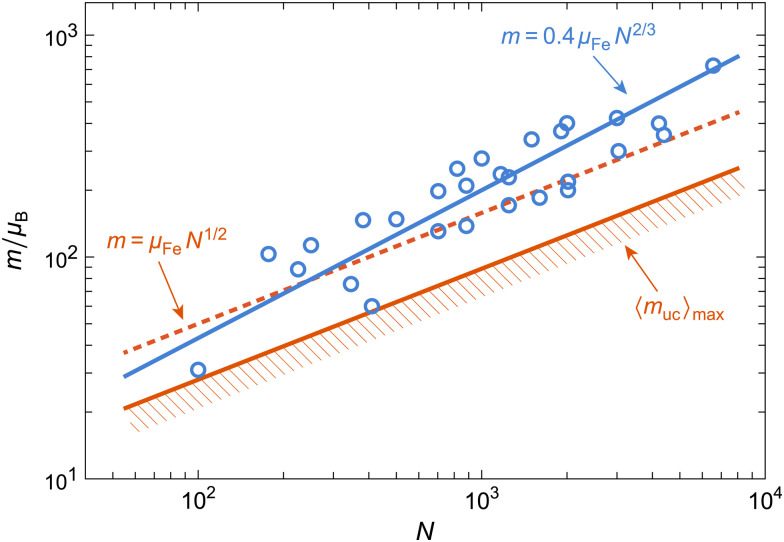
Mean magnetic moment *vs.* number of Fe atoms in ferritin and ferrihydrite particles (circles), obtained from a compilation of literature data.^10,30–32,69–79^ Lines show best fits with different power laws described in the text.

Magnetic moment estimates obtained from ferritin nanoparticles with different iron loadings support a power law of the form *m* ∝ *N*^*p*^ with *p* comprised between 1/2 and 2/3, the latter being the exponent expected from the uncompensated moments arising from surface spins belonging to one sublattice only.^32^ The same empirical power law applies to a large compilation of available data on ferritin and ferrihydrite, which yields *p* ≈ 0.59 ± 0.06, with no systematic differences related to particle composition (Fig. 6). The maximum possible defect moment 〈*m*_uc_〉_max_ associated with *p* = 1/2 is compatible only with two measurements out of a total of 28, so that the Néel defect model must be discarded. The empirical trend 〈*m*〉 ≈ 0.4*μ*_Fe_*N*^2/3^ fits the data almost optimally. Its compatibility with a surface-spin-canting origin of the magnetic moment is discussed below.

A better insight into the origin of the peculiar magnetic properties of ferritin is provided by the joint analysis of the magnetic moment and anisotropy distributions, through the relation between *m*, *B*_a_, *K*, and *T*_b_ imposed by the Néel–Arrhenius model6
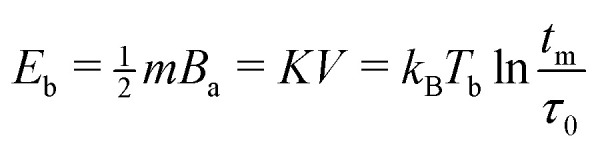
for the energy barrier *E*_b_ of uniaxial single-domain particles, with *t*_m_ being the measurement time and *τ*_0_ the attempt-time for spin reversal (see Section 5.2).

We also tried the alternative model of fitting measured equilibrium magnetization curves with two distinct magnetic moments, as proposed by Brem *et al.*^27^ Replication of this approach with our data yields superparamagnetic and linear contributions that are almost identical to our original model, with a slightly smaller misfit (Fig. S20, ESI†). The magnetic moments *m*_1_ = 97*μ*_B_ and *m*_2_ = 540*μ*_B_ and the relative superparamagnetic contributions (57% and 43%, respectively) are similar to those obtained by Brem *et al.* for horse-spleen ferritin, where *m*_2_ was attributed to magnetite. Using a magnetic moment of 4.1*μ*_B_ per formula unit of Fe_3_O_4_, magnetite must contain ∼20% of the ∼2000 Fe atoms in our ferritin cores in order to explain the magnetic moment of 540*μ*_B_ attributed to this phase. The model of Brem *et al.* requires the two moments to be fully uncoupled in order to be modeled by the linear combination of two independent Langevin functions, in which case their contributions to the remanent magnetization would add linearly in the same proportions as the superparamagnetic contributions. However, the magnetite-like component deduced from our IRM measurements contributes to ∼0.6% of the total blocked magnetization, much less than deduced from the two-moment Langevin model.^27^ This discrepancy is too large to be explained by uncertainties in the Langevin fit or in fits of the IRM acquisition curves. Therefore, we must conclude that ferritin cores are made either by a single phase or by different phases with rigidly coupled spins, justifying the representation of the core magnetization by a fixed magnetic moment.

### 3.4 Surface-spin model for ferritin

As discussed above, surface spin canting in ferritin cores appears to be the only source of a spontaneous magnetic moment that is compatible with all magnetic characterizations reported so far. This model represents the basis of our approach to fit our equilibrium magnetization curves and obtain the magnetic moment distribution (see Section 4.4). Along with the anisotropy field distribution obtained from the analysis of IRM acquisition curves, these results allow to verify the consistency of all magnetometry measurements, as explained in the following.

If the magnetic moment of ferritin cores is controlled by surface effects, we can expect *m* = *N*_s_*μ*_Fe_*η*_s_ to be proportional to the number *N*_s_ of surface spins, their magnetic moment *μ*_Fe_ ≈ 5*μ*_B_, and the degree *η*_s_ = sin *ε*_s_ of canting, regardless of the detailed spin configuration. Internal spins might also experience some canting through coupling with the surface.^66^ In all cases, the source of spin canting is related to the surface, and therefore, we assume *N*_s_ = *κ*_s_*V*^2/3^ with *κ*_s_ = (36π)^1/3^(*ρ*_f_*N*_A_/*u*_f_)^2/3^, where *ρ*_f_ ≈ 3.9 g cm^−3^ is the density of ferritin cores,^80^*N*_A_ the Avogadro constant, and *u*_f_ ≈ 96 g mol^−1^ the molar mass per Fe atom obtained with the chemical formula 5Fe_2_O_3_·9H_2_O of six-line ferrihydrite. The resulting expression *m* = *κ*_s_*μ*_Fe_*η*_s_*V*^2/3^ explains the data compilation in Fig. 6, yielding *η*_s_ ≈ 0.4 if only surface spins are canted. Much lower canting angles are required if spin canting extends to internal spins.^66^ Alternate compositions have been proposed for the ferritin mineral core:^17^ if this composition is indeed significantly different from that of ferrihydrite, it does not affect the magnetic moment, as seen in Fig. 6.

According to the above model for the magnetic moment, the distribution of ln*m*, which is usually assumed to be Gaussian when fitting *M*(*B*) curves, is given by *g*_m_ = *g*_*κμη*_ * *g*_*V*^2/3^_, where *g*_*κμη*_ and *g*_*V*^2/3^_ are the distributions of ln(*κ*_s_*μ*_Fe_*η*_s_) and ln *V*^2/3^, respectively, and “*” is the convolution operator. Deconvolution of *g*_m_ obtained from fitting *M*(*B*) curves with *g*_*V*^2/3^_ obtained from TEM observations thus yields an estimate of *g*_*κμη*_, from which the distribution of *η*_s_ easily derived. The maximum range of this distribution should not exceed *η*_s_ = 1 for a physically reasonable spin canting model.

The surface spin model must also satisfy the Néel–Arrhenius expression for the energy barrier (eqn (6)) when the distributions of *m*, *B*_a_ and *T*_b_ are considered. The normalized temperature dependence *M*_r_(*T*)/*M*_r_(0) of the saturation remanent magnetization *M*_r_ yields, by definition, the integral of the blocking temperature distribution, *f*_b_(*T*). The function *f*_b_(*T*) can also be reconstructed from *E*_b_ = *mB*_a_/2 using the distributions of *m* and *B*_a_ obtained from *M*(*B*) and from IRM acquisition curves, respectively. These distributions, however, are extremely broad, so that the product *mB* depends critically on the type of relation existing between *m* and *B*, and not just on the respective mean values. In the case of ferrimagnetic SD particles with spontaneous magnetization *M*_s_, *m* = *M*_s_*V* and *B*_a_ = 2*K*/*M*_s_ are independent variables, because *M*_s_ is a fixed material property. Accordingly, the distribution of ln(*mB*_a_) is given by the convolution of the distributions of ln*m* and ln*B*_a_, respectively. This approach, however, does not hold for AF particles, where *M*_s_ = *m*/*V* is itself distributed. Accordingly, in the case of ferritin, convolution of the distributions of ln*m* and ln*B*_a_ yields an extremely broad distribution of energy barriers, which does not match *f*_b_(*T*) (Fig. S21, ESI†).

The correct expression for the anisotropy field of AF particles obtained from eqn (6) is *B*_a_ = 2*K*/*m*: in this case, *B*_a_ is inversely proportional to the magnetic moment, and the two factors in *E*_b_ = *mB*_a_/2 are no longer independent variables. The inverse relation between *m* and *B*_a_ can be understood by considering that the work required to reverse all spins of the AF lattice must be provided by the Zeeman energy of the magnetic moment in the switching field *B*_sw_, so that a smaller magnetic moment must be compensated by a larger *B*_sw_ ∝ *B*_a_. The above model for *m* yields *B*_a_ = 2*KV*^1/3^/*κ*_s_*μ*_Fe_*η*_s_. If *V* is a narrow distribution, as in the case of ferritin cores, any size dependence of *K* and *η*_s_ is negligible, and *B*_a_ is the product of almost completely independent statistical variables. The resulting anisotropy field distribution is then given by *g*_a_ ≈ *g*_2*K*/*κμ*_ * *g*_*V*1/3_ * *g*_*η*−1_, where *g*_2*K*/*κμ*_, *g*_*V*1/3_, and *g*_*η*−1_ are the distributions of ln(2*K*/*κ*_s_*μ*_Fe_), ln *V*^1/3^, and ln *η*^−1^, respectively so that *g*_a_ can be reconstructed from estimates of *K*, *V*, and *m* obtained from magnetometry measurements. On the other hand, *g*_a_ can also be obtained directly from IRM acquisition curves, using the well-known relation *B*_a_ = 2.083*B*_sw_ for randomly oriented, uniaxial SD particles.^81^ The two reconstructions of the anisotropy field distribution must coincide if the surface spin model described above correctly describes the spin configuration of ferritin cores.

## 4 Results

### 4.1 Electron paramagnetic resonance

**Fig. 7:**
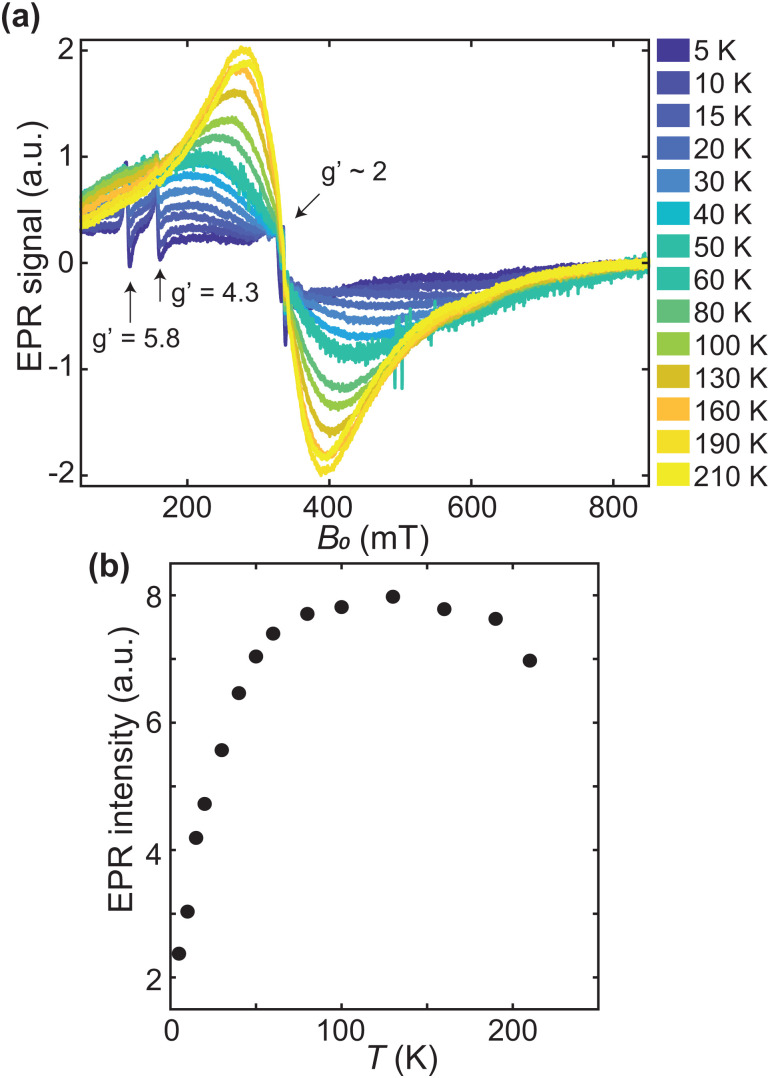
(a) EPR spectra acquired between 5 and 210 K (in order of ascending amplitude from lowest to highest temperature). Arrows point to the narrow signals centered at *g*′ = 2, 4.3, and 5.8, respectively. (b) The double integral of the broad signal component in (a), as a function of measurement temperature.

Fig. 7a shows the continuous wave 9 GHz EPR spectra between 5 and 210 K. The most prominent feature is a broad signal with a linewidth of ∼100 mT at 190 K, which is centered at *g*′ = 2.0. This broad signal is due to the mineral core in the protein shell of ferritin, in agreement with what has been reported in literature.^13,14,21,22,25^ Three narrow signals overlap with the broad spectrum at *g*′ = 2.0, 4.3, and 5.8, respectively (arrows). The *g*′ = 4.3 and *g*′ = 5.8 signals are usually attributed to mononuclear rhombic Fe(iii) sites^13,21,22,52,82^ and high-spin Fe(iii) in methemoglobin,^83^ respectively. Multiple lines at *g*′ = 2.0^52,83^ might be ascribed to radical impurities, Cu(ii), and possibly a small indication of Mn(ii),^22^ but the origin is not further investigated. The amplitude of the *g*′ = 4.3 signal is inversely proportional to the logarithm of temperature, as expected from a paramagnetic contribution (data not shown). The other two narrow signals at *g*′ = 2.0 and 5.8 follow qualitatively the same trend.

The lineshape of the broad signal, which is due to the magnetic moment of ferritin cores (Fig. 7a), is nearly Lorentzian at higher temperatures. Below 100 K, the shape becomes more asymmetric and is better fitted to a Gaussian shape. At 5–10 K, the amplitude of the broad signal has decreased to the point of being barely identifiable. The double integral of the broad EPR component, which reflects the total number of ferritin-core spins in the sample, increases with temperature, reaching a plateau at 100 K, followed by a slight decrease above 180 K (Fig. 7b). The increase in EPR signal amplitude with temperature is typical for superparamagnetic particles with an antiferromagnetic ground state,^14,21,23,43^ and can be explained by the fact that only unblocked particles, whose fraction increases with temperature, contribute to the signal. Once all particles are unblocked, a paramagnetic behavior, characterized by a decrease of the EPR spectral amplitude with increasing temperature is observed. Therefore, the maximum value of the signal intensities (Fig. 7b), at *T* ∼ 100 K, marks the transition from a regime of progressive unblocking of the magnetic moments to a regime where all moments are unblocked, thus representing the maximum blocking temperature of ferritin cores.

EPR simulations provide further insights into the nature of the broad signals. For this purpose, the ferritin core is considered as a single large spin *S* resulting from the coupling of individual iron ions in the core.^50,51^ The corresponding spin Hamiltonian, used to describe the spectra, is given by

7 ℋ = *μ*_B_**S**·**g**·**B** + **S**·**D**·**S**,

with *μ*_B_ being the Bohr magneton, **S** the electron spin operator associated with the spin number *S*, **g** the *g* tensor, **D** the traceless zero-field splitting tensor, and **B** the applied magnetic field. In relation to magnetometry, the total spin of a particle can be expressed as^51^8
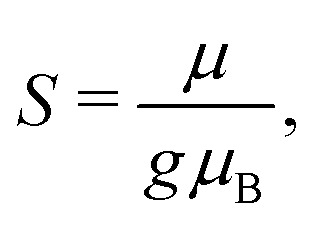
with *μ* being the effective magnetic moment of the particle.^51^ The zero-field splitting is then expressed by9
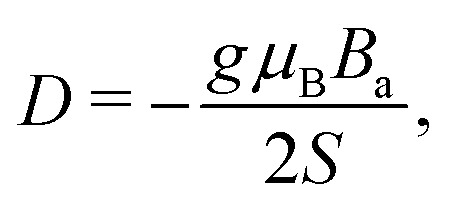
with *B*_a_ being the effective anisotropy field.^42^ In case of particles with uniaxial anisotropy, *B*_a_ = 2*KV*/*m*, with *K* being the anisotropy factor and *V* the particle volume.

Simulations based on a single component produce a poor fit to the data (Fig. S5, ESI†), suggesting that at least two components are needed in order to capture the relevant spectral features. Automated two-component fitting approaches, however, yield unphysical results even in the case of limited parameter sets (see ESI:† “EPR alternative fitting approach”). This is because the line shape of high-spin systems such as ferritin does not depend in a simple manner on *D* and *S*. Furthermore, very different component combinations can fit broad line shapes equally well, so that meaningful fitting results depend critically on the initial parameter guess.

Physically meaningful initial parameter guesses have been obtained at selected temperatures by visually matching the measured spectra with a set of components covering a wide range of values for *D*, *S* and the Gaussian broadening parameter *H*_strain_ (see ESI:† “EPR simulations of individual components”). Because of the excessive computation time required for simulating realistic values of *S* in excess of ∼100, the scaling procedure of Fittipaldi *et al.*^50,51^

**Fig. 8:**
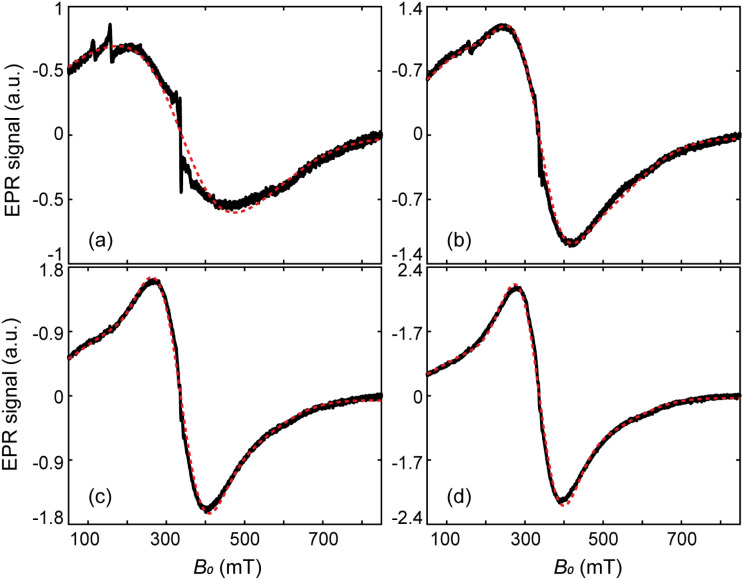
Selected EPR spectra (black lines) and their simulations (red dashed lines), at 30 K (a), 80 K (b), 130 K (c), and 190 K (d).

**Fig. 9:**
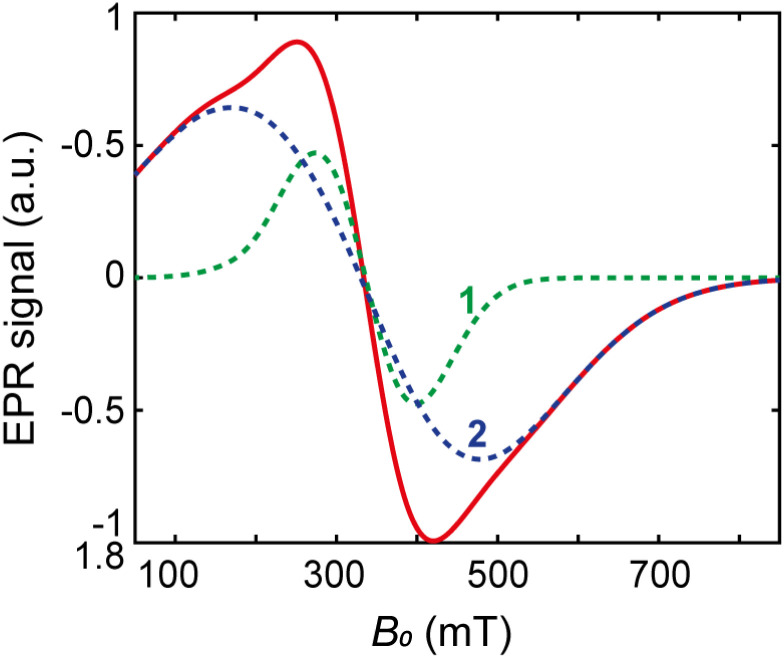
Total EPR spectrum (solid line) and components E1, E2 (dashed lines) at 80 K.

10 *S*_real_ = *S*·*n*, *D*_real_ = *D*/*n*, *T*_real_ = *T*·*n*

has been used to relate realistic parameters to those used for fitting, through a scaling factor *n*. For instance, a simulated spectrum with *S* = 100 is obtained by rescaling a corresponding calculation performed for *S* = 10, using *n* = 10. The combination of two model spectra with *g*′ = 2.01 and *S* = 10, which most closely reproduced a chosen experimental spectrum, served as initial guess for the final optimization of the component-specific parameters *D* and *H*_strain_. Simulated spectra obtained with this procedure are in excellent agreement with experimental data at each measurement temperature (Fig. 8, see Fig. S6, ESI† for all temperatures). Details of the optimization procedure are explained in Material and Methods, and the optimized model parameters are listed in Table S1 (ESI†). Both components are centered at *B*_0_ = 336 mT, but the first component (E1) is significantly narrower than the second one (E2) (Fig. 9a, see Fig. S7, ESI† for other temperatures). The temperature dependence of E1 and E2 is small compared with the respective error margins (Fig. S8, ESI†). A systematic variation of *D*, *H*_strain_, and the relative contributions of E1 and E2 cannot be excluded, but the trend is not sufficiently well defined to support further interpretations.

### 4.2 DC susceptibility and hysteresis

**Fig. 10:**
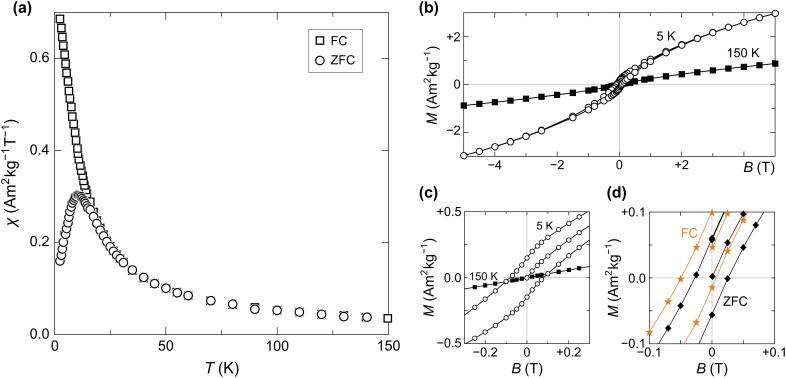
(a) ZFC–FC magnetization curves, measured at 5 mT. (b) Isothermal induced magnetization measured at 150 K (full squares) and 5 K (empty circles). Both temperatures were reached in ZFC conditions. (c) Close-up of (b) centered on the origin, showing hysteresis opening at low temperature. (d) Detail of ZFC (black diamonds) and FC (orange stars) hysteresis around the origin, measured at 5 K in a maximum field of 0.3 T.

The superparamagnetic behaviour of human-liver ferritin is well captured by FC-ZFC measurements (Fig. 10a). The curves bifurcate at *T*_b,max_ ≈ 24 K, which corresponds to the largest unblocking temperature of the particles. The ZFC data display a peak at *T̂*_b_ = 10.5 ± 0.5 K, in agreement with an earlier characterization of ferritin.^10^ The relation *T̂*_b_ < *T*_b,max_ is indicative of a distribution of blocking temperatures. The opening of the hysteresis loop below *T̂*_b_ confirms the blocking process and the occurrence of magnetic irreversibility (Fig. 10b). The slight horizontal offset of the FC hysteresis loops highlights the presence of an exchange coupling field *B*_ex_ ≈ 25 mT, persistent at 5 and 25 K (Fig. 10d). This exchange field is similar to *B*_ex_ ≈ 32 mT reported for horse-spleen ferritin.^10^

**Fig. 11:**
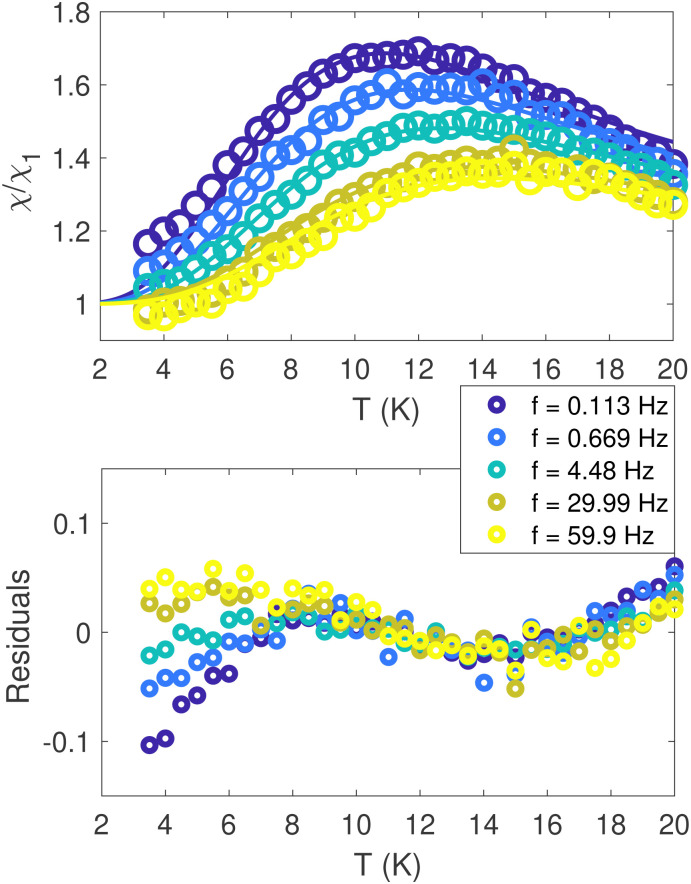
AC-magnetic moment and susceptibility of human-liver ferritin, probed in the sub-kHz frequency range. Top panel: Normalized in-phase susceptibility data (circles) and fit (solid line) to eqn (11). Bottom panel: Residuals of the fit.

**Fig. 12:**
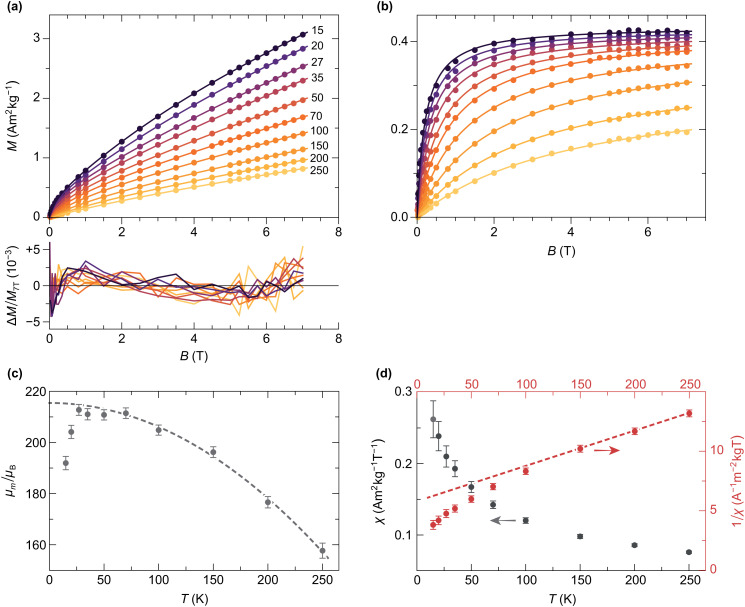
(a) Measured isothermal magnetization curves (dots) and corresponding best fits with eqn (12) (lines), at temperatures indicated by numbers. Residuals, defined as the difference between measurements and model, normalized by the measured magnetization at 7 T, are plotted below. (b) Same as (a), after subtracting the modeled linear term from each curve. The 15 and 20 K magnetizations have been multiplied by 1.13 and 1.05, respectively, for better visualization. (c) Logarithmic mean of the magnetic moment distribution at measurement (dots, with 2*σ* error bars). The dashed line is the best-fitting antiferromagnetic magnon law obtained from >50 K moment estimates, with *μ*_m_(0) = 215.4*μ*_B_ and *α* = 0.0043. (d) Non-paramagnetic susceptibility *χ* from the *χB* term in eqn (12) (black dots with 2*σ* error bars, left axis), and 1/*χ* (red dots with 2*σ* error bars, right axis). The dashed line represents the best-fitting Curie–Weiss law above 150 K, with *Θ* = −194 K.

### 4.3 AC susceptibility

The in-phase AC susceptibility *χ*′(*T*) (Fig. 11) shows a broad peak that shifts towards higher temperatures upon increasing the frequency of the AC field.^84^ In-phase measurements have been fitted to a model derived from Gittleman *et al.*,^85^ while the imaginary part was ignored, due to its low SNR (see Fig. S18, ESI†). Full *χ*′(*T*) curves were calculated by integrating the analytical expression^85^ for *χ*′ over a distribution *Γ*(*E*_b_) of anisotropy energy barriers *E*_b_, thereby relaxing any assumption about the analytical dependence of *E*_b_ on the particles volume distribution:11
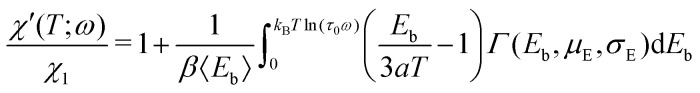
where *ω* = 2π*ν* is the AC frequency in rad s^−1^, *τ*_0_ is the inverse attempt frequency of thermal activations, *χ*_1_ is the susceptibility in the blocked state, *a* = 〈sin^2^*ϕ*〉/2, where the average is over all angles *ϕ* between easy axis and field, with *a* = 1/3 in case of random orientations, and *β* is a scaling factor with a theoretical value of 1 for blocked particles described by the model of Stoner and Wohlfarth.^81^ We note that the single-domain susceptibility of blocked particles is not temperature-independent as assumed in the fitting equation, since it depends on the temperature-dependent anisotropy constant and spontaneous magnetization. Therefore, this remains a coarse approach to determine *E*_b_.

Eqn (11) was used to fit the AC susceptibility measurements using the Gamma function *Γ*(*E*_b_,*μ*_E_,*σ*_E_) with mean *μ*_E_ and width parameter *σ*_E_ as energy barrier distribution.^54^ The attempt time was fixed to 9 ps, based on reported AC susceptibility ferritin data.^30^

The mean and standard deviation of the energy-barrier distribution used to fit the data (eqn (11)) are 144.74 K and 57.02 K, respectively. Note that the scaling parameter, *β*, decreased by 30%, upon decreasing the frequency.

### 4.4 Equilibrium magnetization

Isothermal magnetization curves acquired in fields up to 7 T at temperatures ≥15 K (Fig. 12a) are close to equilibrium: the residual hysteresis opening at 15 K is ∼3% of *M*_s_, and drops to ∼0.5% at 27 K. A modified Langevin model of the form *M*(*B*) = 𝒮(*B*,*f*) + *χB* was used to fit the equilibrium magnetization, where 𝒮 is a superparamagnetic term resulting from the partial alignment of unblocked magnetic moments with distribution *f* in the applied field, and *χB* is a linear term that includes superantiferromagnetic and paramagnetic contributions. In case of isotropic particles with identical magnetic moments *m* that are sufficiently large to ignore quantization effects, the superparamagnetic term is proportional to the Langevin function ℒ(*ξ*) = coth*ξ* − *ξ*^−1^ with *ξ* = *mB*/*k*_B_*T*, where *k*_B_ is the Boltzmann constant, and *T* the absolute temperature.^30,32^ This model reproduces the equilibrium magnetization calculations for canted spins discussed in Section 3.2 (Fig. 5).

Single-particle magnetic anisotropy decreases the magnetic moment alignment of mechanically blocked, randomly oriented particles as soon as the linear regime of ℒ is left.^59,86,87^ In the limit case of infinite uniaxial anisotropy, particles possess only two magnetic states with *m* parallel or antiparallel to the easy axis, in which case ℒ is replaced by 𝒢(*ξ*) = 〈cos *ϕ* tan h(*ξ* cos *ϕ*)〉, where 〈·〉 denotes the average of individual particle contributions over all angles *ϕ* between easy axes and field. This function was originally proposed by Néel,^61^ and used by Gilles^41^ as a model for ferritin superparamagnetism. ℒ and 𝒢 have the same slope at *B* = 0, but their *B* → ∞ limits are 1 and 1/2, respectively. Particles of volume *V* and finite anisotropy constant *K* are characterized by intermediate equilibrium magnetization functions comprised between ℒ and 𝒢, whose shape is controlled by the anisotropy parameter *κ* = *KV*/*k*_B_*T*. Unfortunately, these functions cannot be expressed analytically, so that ℒ or 𝒢 are used instead, regardless of the effective particle anisotropy.^10,30,56,68,87^ The Langevin function is a good approximation of the superparamagnetic behavior for *κ* < 2 (maximum error: 2%), and still considerably better than 𝒢 for *κ* < 10 (maximum error: 20%). Using *K* = 18.3 kJ m^−3^ for our ferritin sample (see Section 5.3) and the volume of spherical cores with a diameter of 7 nm (Fig. 1), the *κ* < 2 and *κ* < 10 conditions are fulfilled for *T* > 100 K and *T* > 24 K, respectively, which means that ℒ is a valid model for all measurements shown in Fig. 12a, except the 15 K one.

Implementation of the superparamagnetic term for a single-valued magnetic moment yields large model residuals with systematic trends (Fig. S17, ESI†), indicating that the real moment distribution is a broad function. Therefore, ℒ needs to be integrated over the moment distribution *f*(*m*), usually assumed to be a lognormal function with unknown logarithmic mean *μ*_m_ and logarithmic standard deviation *σ*_m_.^41,68^ Furthermore, the presence of paramagnetic spins, suggested by EPR measurements, requires to split the linear term of the fitting function into a term associated with the ferritin cores, and another term for the paramagnetic contributions. The resulting model is given by12
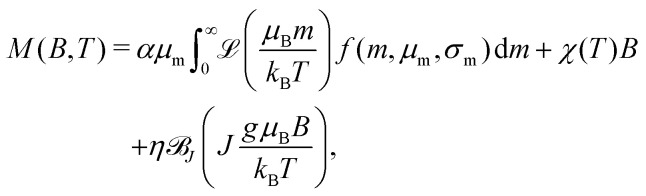
where *α* is a scaling factor, such that *M*_s_ = *αμ* is the saturation magnetization of the superparamagnetic particles, *χ* is the high-field magnetic susceptibility of the cores, and ℬ_*J*_ is the Brillouin function describing the magnetization of paramagnetic spins with total spin quantum number *J* and magnetic moment *gμ*_B_. The scaling factors *α* and *η* account for the unknown concentration of ferritin cores and paramagnetic spins, respectively. The use of *M*_s_ = *αμ* ensures that the saturation magnetization has the same temperature dependence as the magnetic moment, as expected for a superparamagnetic system where saturation is reached by magnetic moment rotation only. The paramagnetic term is justified by the identification of a corresponding EPR component with *g*′ = 4.3, originating from mononuclear Fe^3 +^ ions with *J* = *S* = 5/2. This EPR component covers a large field range and is therefore dominant over other paramagnetic contributions with *g*′ = 2 and 5.8. In order to avoid model instabilities caused by the similar shapes of the Langevin and Brillouin functions, the shape of the latter has been fixed using *J* = 5/2 while maintaining *g* unconstrained.

All *M*(*B*) curves in the 15–250 K range have been fitted globally, that is, with common temperature-independent parameters *α*, *η*, *σ*_m_, *g*, and one set of temperature-dependent parameters *μ*_m_ and *χ* for each curve. The assumption that *σ*_m_ does not depend on temperature is justified by the fact that the width of a broad moment distribution is relatively insensitive to possible differences between the temperature dependencies of small and large magnetic moments. Parameter confidence intervals have been calculated using a Monte Carlo error estimation, which consisted in adding random errors to the data, based on the standard deviation of the random component of model residuals. Residuals are comprised between ±0.5% of the maximum magnetization at 7 T (Fig. 12a), with a common field-dependent trend limited to ±0.2% and a random component associated with measurement errors. The small systematic misfit might be caused by a non-lognormal distribution of magnetic moments, by deviations from the Langevin model due to single particle anisotropy, or by a small field dependence of *χ*. An almost complete saturation of the superparamagnetic contribution in the 7 T maximum field is attained at the lowest temperatures (Fig. 12b), meaning that the moment distribution can be recovered from the data, up to a small fraction of smallest moments, whose magnetization saturates in larger fields.

The temperature dependencies of *μ*_m_ and *χ* (Fig. 12c and d) are qualitatively similar to those obtained by Makhlouf *et al.*^10^ and Gilles *et al.*^56^ The maximum mean moment 〈*m*〉 = exp(*μ*_m_ + *σ*_m_^2^/2) ≈ 333*μ*_B_ is slightly smaller than the single-valued estimate of ∼350*μ*_B_ obtained from horse-spleen ferritin using a simple Langevin fit with linear term.^10^ The temperature dependence of *μ*_m_ is characterized by two opposed trends: a ∼10% increase over the 15–27 K range, followed by a quadratic decrease compatible with the bulk antiferromagnetic magnon law, *μ*_m_(*T*) = *μ*_m_(0)(1 − *aT*^2^) (Fig. 12c).^68^ The initial increase *μ*_m_(*T*) is likely an artifact of the Langevin model. As previously mentioned, the equilibrium magnetization of particles with finite anisotropy becomes proportional to 𝒢(*ξ*) at *T* → 0. In a Langevin fit, this function is approximated by ∼0.5ℒ(2*ξ*). Because *ξ* ∝ *m*, the apparent moment obtained from the Langevin fit decreases as the appropriated model function changes from ℒ(*ξ*) to 𝒢(*ξ*). The non-paramagnetic susceptibility *χ* decreases monotonically with temperature, approaching a Curie–Weiss law with *Θ*_CW_ ≈ −194 K above ∼100 K (Fig. 12d). Deviations from this trend are expected in the case of AF nanoparticles, because of superantiferromagnetic contributions arising for instance from uncompensated spin planes.^39^ Spin frustration might also contribute to *χ*, as seen by similar temperature dependencies encountered in systems dominated by this effect.^88^

### 4.5 Coercivity distributions

**Fig. 13:**
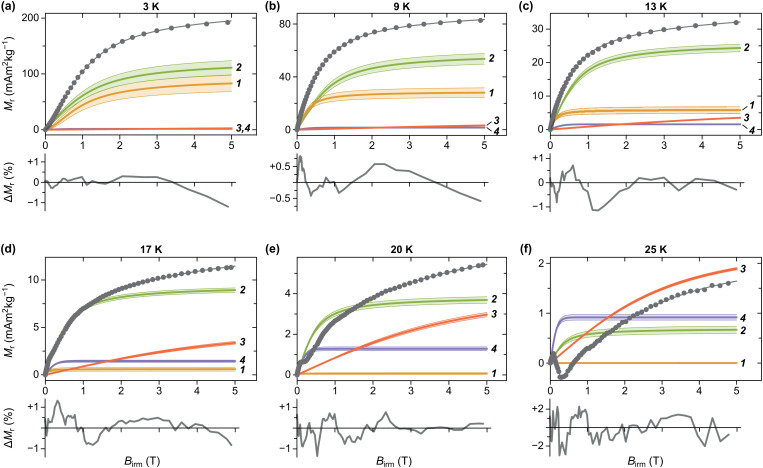
IRM acquisition curves at selected temperatures (dots), model components used to fit the data (solid curves labelled by component number), and modelled total magnetization (unlabelled gray curve). The shaded band around each component correspond to the 1-standard-deviation uncertainty obtained from bootstrap simulations of measurement errors. Model residuals, expressed in percent of the maximum magnetization at 5 T, are plotted below.

IRM acquisition curves *M*_r_(*B*) describe the acquisition of a remanent magnetization from an initially demagnetized state after the application of increasingly large fields, until the so-called saturation remanent magnetization *M*_rs_ is reached. Only particles that are blocked over the time needed to zero the magnetic field and measure the magnetic moment (about a minute in case of MPMS measurements) contribute to the IRM. Accordingly, the amplitude decrease of *M*_r_(*B*) with increasing temperature is caused by the progressive unblocking of magnetic moments (Fig. 13), and *M*_rs_(*T*) is the integral of a blocking temperature distribution probed by remanent magnetization measurements.

IRM curves become non-monotonic around 20 K, with an inflection around 0.15 T. The negative slope section denotes a low-coercivity phase that acquires a negative remanent magnetization. Because of the nature of the IRM acquisition protocol, which is formally equivalent to a partial hysteresis between *B* = 0 and *B*_irm_, negative *M*_r_(*B*) slopes must be associated with inverted hysteresis,^89^ a phenomenon that arises from the exchange coupling between phases with different coercivities. These phases can be detected by fitting *M*_r_(*B*) with a linear combination of model curves representing their individual contributions:13
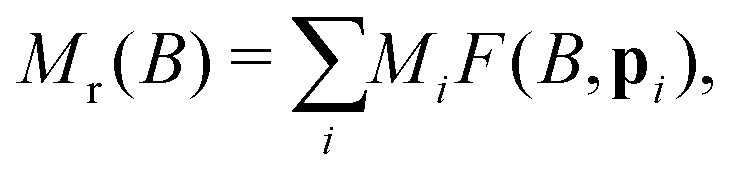
where *M*_*i*_ is the saturation remanent magnetization of the *i*-th component, and *F* the primitive of a model function *f* used to represent the corresponding coercivity distribution,^90,91^ whose shape is controlled by a set of parameters **p**. Assemblages of magnetic particles with uniform composition, size, and shape are characterized by unimodal coercivity distributions, which, on a logarithmic field scale, are well approximated by a normal distribution 𝒩(log *B*,log *μ*_B_,*σ*_B_) with logarithmic mean *μ*_B_ and standard deviation *σ*_B_,^90,92^ or, more often, by slightly left-skewed generalizations of the normal distribution.^91,93^ Depending on the distribution skewness, *μ*_B_ is more or less close to the median acquisition field *B*_1/2_, which is the field required to acquire half of the saturation remanent magnetization.

Coercivity distributions do not have necessarily an intrinsic physical meaning, being just defined as the first derivative of magnetization curves. A notable exception is represented by uniaxial single-domain particles described by the Stoner–Wohlfarth model.^81^ In this case, *f*(*B*) represents the relative contribution of particles with switching field *B*_sw_ = *B* to *M*_rs_. Ferritin behaves as an assemblage of non-interacting Stoner–Wohlfarth particles, as seen from the identity between the shape of IRM acquisition curves and curves obtained by applying the same protocol to samples with a previously imparted negative saturation remanent magnetization.^94,95^ Well below the blocking temperature, the switching field is related to the anisotropy field *B*_a_ = 2*KV*/*m* by *B*_sw_ = *ζB*_a_ with *ζ* ≈ 0.524, if particles are randomly oriented.^96^

A linear combination of four coercivity components of the form14
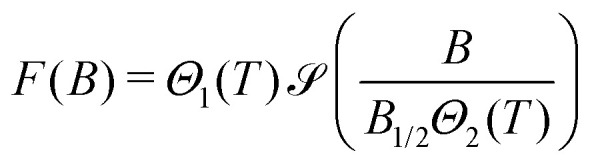
has been used to model IRM acquisition curves at selected measurement temperatures. In eqn (14), 𝒮 (*x*) is a sigmoidal function with 𝒮 (0) = 0 and 𝒮 (∞) = 1, based either on the Langevin or the tanh function, *Θ*_1_(*T*) is a function describing the cumulative blocking temperature distribution of the corresponding component, with *Θ*_1_(0) = 1, *B*_1/2_ is the median acquisition field at 0 K, and *Θ*_2_(*T*) is a monotonically decreasing function describing the decline of *B*_1/2_ with temperature, caused by thermally activated moment switching.^96^ The effects of exchange coupling between a higher- and a lower-coercivity component are modelled by multiplying *F*(*B*) of the lower-coercivity component with a smoothed sign function, centered at the mean value of the exchange field, which roughly coincides with the inflection point of the IRM curve (ESI:† “Equilibrium magnetization models”).

**Fig. 14:**
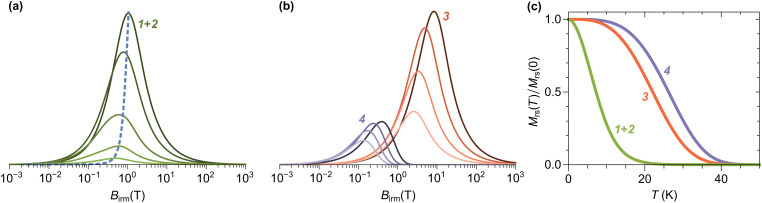
(a) Logarithmic switching field distribution of Components 1 + 2 at 3, 5, 9, 13, and 17 K (solid lines in order of decreasing amplitude), and the predicted temperature dependence of the peak for particles with randomly oriented uniaxial anisotropy axes (dashed line). (b) Same as (a) for components 3 and 4. (c) Fraction of blocked magnetic moments for components 1 + 2, 3, and 4, as a function of temperature.

Below ∼15 K, the IRM is dominated by two coercivity components, C1 and C2, which contribute to 96% of the total *M*_rs_. The blocking temperatures of the other two components, C3 and C4, are much larger than those of the bulk sample, contributing mainly to the IRM curves acquired at 20 and 25 K. In the case of C4, the maximum *T*_b_ is close to that of magnetoferritin.^97^ C1 and C2 are characterized by slightly different temperature and field dependencies, with *B*_1/2_ ≈ 1 T at 3 K. C3 and C4 are characterized by strongly contrasting median acquisition fields, with *B*_1/2_ ≈ 3.2 T and ∼0.12 T, respectively, at 20 K. C3 is heavily unsaturated at 5 T. Extrapolation of the model function used to fit this component to higher fields suggests that saturation occurs above 50 T (Fig. 14b), similarly to what has been reported for goethite.^98^ The field dependence of C4 is within the range that can be expected from ferrimagnetic minerals: equidimensional magnetite and maghemite nanoparticles with sizes above 4 nm are characterized by coercivities of 40–50 mT at 5 K.^99,100^ The larger median field of C4 might be explained by additional contributions from shape and surface anisotropy, in the case of smaller, irregular crystals partially replacing the Fh core.

C3 and C4 are coupled by an exchange field *B*_ex_ ≈ 82 mT. At fields ≪*B*_ex_, C4 acquires a significant fraction of its saturation remanent magnetization, while C3 is still close to its initial demagnetized state, owing to its much larger coercivity. As C3 becomes progressively magnetized in the positive direction, negative exchange coupling causes C4 to be switched to the opposite direction, leading to a decrease of the total IRM. When C4 is negatively saturated, around 200 mT, the total IRM starts to increase again, due to the continuing IRM acquisition of C3. The non-monotonic IRM acquisition characteristics of these two negatively coupled components becomes clearly visible above 20 K, when C1 and C2 are almost completely superparamagnetic, no longer contributing to the remanent magnetization.

While the identification of C3 and C4 with independent entities is justified by the exchange coupling signature, the existence of C1 and C2 as independent components, instead of a single component, might just reflect the need to use two model functions to describe the complex shape of a single coercivity distribution.^91^ Because of their similar field and temperature dependencies, C1 and C2 are merged into a single component, labelled as C1+2. C1+2 is characterized by a very broad coercivity distribution, which extends over ∼4 orders of magnitude (Fig. 14a), and a maximum blocking temperature of ∼20 K (Fig. 14c), which is close to the merging point of FC-ZFC low-field magnetization curves (Fig. 10a). The temperature dependence of *B*_1/2_, which can be identified with the coercivity distribution peaks in Fig. 14a, is well described by the thermal activation model of Egli and Lowrie^101^ for the switching field of randomly oriented single-domain particles with uniaxial anisotropy energy *mB*_a_/2, when *B*_a_ = 2*B*_1/2_(*T* = 0) is taken from the extrapolation of the IRM fitting model to 0 K, and *m* = 325*μ*_B_ is assumed (dashed line in Fig. 14a). The required magnetic moment is close to the mean value of ∼333*μ*_B_ derived from the Langevin model of isothermal magnetization curves. Consideration of the random particle orientation is very important, as simpler thermal activation models based on aligned anisotropy axes^102^ require unrealistically large moments of the order of ∼1000*μ*_B_ to fit the distribution maxima in Fig. 14a.

## 5 Discussion

**Fig. 15:**
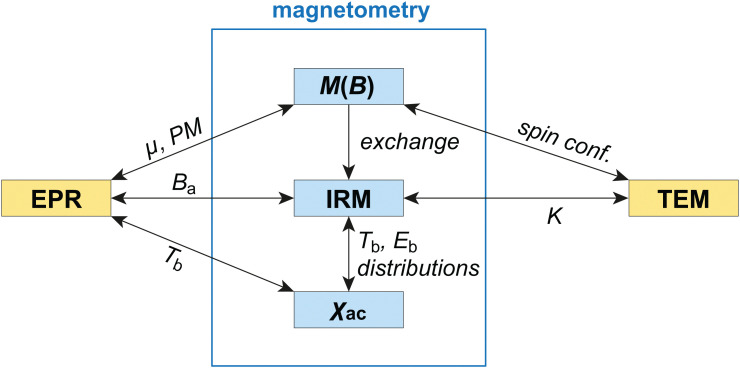
Schematic representation of different techniques used for ferritin characterization and their use for the determination of relevant properties. Abbreviations: PM—paramagnetic contribution, exchange—exchange coupling between magnetic components, spin conf.—spin configuration. For the rest of the symbols, the reader is referred to the main text.

In this work, we present a comprehensive investigation of human-liver ferritin by in-depth electron paramagnetic resonance and an extensive set of magnetometry techniques. The goal is to determine the spin-structure of ferritin in order to elucidate the composition of the ferritin core in terms of magnetic phases. Fig. 15 shows the combination of techniques used to determine the properties of ferritin cores and produce a model for their spin configuration. Magnetometry measurements yield the magnetic moment, blocking temperature, and energy barrier distributions, while EPR provides an important constraint on the paramagnetic contribution to *M*(*B*) curves, as well as independent estimates of the anisotropy field and the blocking temperature over a much shorter time range of the order of 0.1 ns. Comparison with magnetometric blocking temperatures permits to verify the Néel–Arrhenius law and estimate the attempt time of thermal activations. The volume distribution of ferritin cores is obtained from TEM observations and yields, in combination with the anisotropy field distribution, an estimate of the magnetocrystalline anisotropy constant (see Section 5.2 for details). The volume and magnetic moment distributions provide also important constraints on the spin configuration of ferritin cores.

The combination of this unusually broad set of experimental data shows that several of the previous approaches to interpret ferritin data give inconsistent results, requiring a new theoretical model to fit the data and derive the magnetic properties of this iron-oxide nanoparticle. One of the crucial findings is that the distribution of magnetic parameters that do not reflect intrinsic properties of the core material, such as *μ*(*m*) and *B*_a_, need to be taken into account explicitly.

The following discussion starts with the properties of ferritin derived from the EPR analysis, and continues with key parts of the analysis of magnetometry results. The latter leans heavily on Section 3, which describes the framework on which the interpretation is based. The discussion is concluded by the joint interpretation of EPR and magnetometric models, leading to the description of the spin structure of the core of human-liver ferritin.

### 5.1 EPR simulations

In this section we describe the EPR spectra of ferritin, which are characterized by broad signal attributed to the core material (Fig. 7). Compared to standard EPR signals, ferritin spectra are extremely broad and the changes with temperature are small, thus lacking clearly resolved features. The several hundred mT width and the overall Gaussian shape at temperatures smaller than 100 K suggest an ensemble of ferritin cores with slightly different properties resulting in a distribution of EPR parameters. To analyze the EPR spectra, we use a quantum-mechanical description of the magnetic properties of ferritin cores, and in particular, we focus on the lineshape and its temperature dependence. This approach allows us to directly obtain the spin structure of ferritin from the simulated EPR spectra. For other approaches see “Other possible approaches…” in ESI.†

There are several challenges associated with analysing the EPR spectra. For high-spin systems such as ferritin, variations in the lineshape due to the spin state *S* and the zero-field splitting *D* are not systematic (see Fig. S13, ESI†), making it challenging to predict which parameters should be used in the simulations of the EPR spectra. Therefore, equivalent simulated spectra can be obtained by many different sets of EPR parameters, such as *S* and *D*, defying standard optimization methods. As an illustration of this problem, an attempt to perform an automatic fit by varying just a few parameters did not lead to a global minimization of the model misfits (see ESI:† “EPR alternative fitting approach”). Therefore, a different approach based on the use of carefully chosen model spectra (see Section 4.1) was used, as discussed in the following.

A minimum of two components are required to simulate the experimental spectra in a satisfactory manner (Fig. S5, ESI†*vs.*Fig. 8b). Simulations with more than two components were not attempted, to avoid an excessive number of free parameters. No assumptions were made about the temperature behaviour, such as, for example, that the relative weight of the components or the *D* values have to be constant for all temperatures, even if these assumptions might be justified by certain models.^50^ The model parameters for the two components E1 and E2 were selected from simulated spectral lineshapes obtained for a range of *D* and *S* values (see: “EPR Model simulations”, and Fig. S13, ESI†). A summary of the parameters used in the final simulations is given in Table S1 and Fig. S8 (ESI†). The temperature dependence of the parameters does not exclude a systematic variation of *D* with temperature, but the trend is not sufficiently well defined to support further interpretations.

How different are the two components E1 and E2 chosen for fitting EPR measurements? For all temperatures, *D*_1_ is 2.5 times smaller than *D*_2_, on average. However, *D* and *S* are not independent, but inversely related (eqn 9), so that a change in *D* can be compensated by the inverse change in *S* without changing the overall shape (see ESI:† “EPR *S*–*D* inverse compensation”). For instance, in the case of equal *D* values for both components, *S*_1_ must be ∼2.5 times smaller than *S*_2_ to obtain the same total EPR spectrum. The two components do not necessarily represent two distinct families of ferritin, rather they should be considered as a mathematical construct to represent an overall broad distribution. The hypothesis of an underlying broad distribution is supported by the fact that the Gaussian broadening used in the simulations exceeds the *D* parameters by at least an order of magnitude, which can only be explained by a distribution of ferritin-core spin configurations within the ferritin population. The overall Gaussian lineshape of the EPR signal below 100 K reveals an inhomogeneous broadening typical of a resonance that consists of centers with a distribution of anisotropic magnetic parameters. The resonance narrows at higher temperatures, yielding an overall Lorentzian lineshape above 100 K, revealing that a dynamic process averages the differences in the anisotropy of the different centers. Such a line narrowing at higher temperatures was attributed to anisotropy averaging^46^ or, alternatively, anisotropic melting.^47,48^

#### 5.1.1 Scaling model of EPR parameters

The real value of *S* for ferritin cores must be much larger than *S* = 10 used in our simulations. The scaling approach proposed by Fittipaldi *et al.*^50^eqn (10) permits to find equivalent parameters *S*_real_ and *D*_real_ corresponding to more realistic magnetic moment estimates, while maintaining the same lineshape. Two examples with scaling factors *n* = 10 and *n* = 30 are given in Table 1.

**Table 1:** EPR parameter scaling and corresponding magnetic moments *μ* for *T* = 20 K. *n* is the scaling factor and *B*_a_ the scale-independent anisotropy field

*n*	**E1** (*B*_a_ = 0.1 T)		**E2** (*B*_a_ = 0.3 T)		E1 & E2
	*S*_real_	*D*_real_ (MHz)	*S*_real_	*D*_real_ (MHz)	*μ* (*μ*_B_)
1	10	−180	10	−450	20
10	100	−18	100	−45	200
30^a^	300	−6	300	−15	601

a Maximum scaling factor, see text.

EPR results can be compared with ferritin properties reported in the literature by deriving *μ* and *B*_a_ from *S*_real_ and *D*_real_ through eqn (8) and (9), respectively. Our range of estimates for *μ* is within the range published by Brem *et al.*^27^ (128–556*μ*_B_) and by Koralewski *et al.*^103^ (133–239)*μ*_B_. On the other hand, our estimated interval of 0.1–0.3 T for *B*_a_ is independent of scaling, and agrees well with the 0.08 ≤ *B*_a_ ≤ 0.27 T range reported in the literature^13,104^ for 5.4–7.0 nm ferritin cores. Previous EPR studies on horse-spleen and human-spleen ferritin focused on describing qualitative aspects of the EPR lineshape (EPR intensity and width variations *vs.* temperature and different ferritin samples), all agreeing on the broad signal located at *g*′ = 2.0 and its observed temperature dependence.^13,14,21,23,24,105^ However, a few EPR spectra present a more asymmetric lineshape, especially at lower fields,^23,24^ the shape of which does not agree well with our spectra.

### 5.2 Ferritin properties derived from magnetometry

Combined analysis of *M*(*B*) curves, AC susceptibility and IRM curves highlights the inconsistency of models used in the literature to interpret the magnetic properties of ferritin (see Section 3). IRM acquisition curves (Fig. 13) support the existence of multiple magnetic phases. The main phase, which we attribute to Fh cores, has a median blocking temperature of ∼8 K and contributes to ∼96% of the remanent magnetization at 3 K. The remaining magnetization is carried by a magnetite-like low-coercivity phase (∼0.6%), exchange-coupled with a high-coercivity phase, which become dominant above ∼17 K. The very large saturation field of the high-coercivity phase is compatible with goethite nanoparticles,^98^ and possibly also wüstite.^106^ The contribution of these two secondary phases in our sample is negligibly small, compared to estimates obtained with diffractometric techniques (*e.g.*, 30% magnetite in horse-spleen and human-liver ferritin).^6^ While these secondary phases must coexist within the same protein shells due to their exchange coupling, it is not clear whether they represent fully altered ferritin cores, or if they coexist with Fh as intermediate alteration products. In any case, non-Fh phases detected with the analysis of IRM curves are negligible. The main IRM component exhibits a magnetic behavior explainable with a fixed total magnetic moment, whose distribution is given by the Langevin model described in Section 4.4. This does not exclude that this component consists of two or more phases with a sufficiently strong magnetic coupling, such that the vector sum of their magnetic moments does not change during isothermal magnetization measurements.

**Fig. 16:**
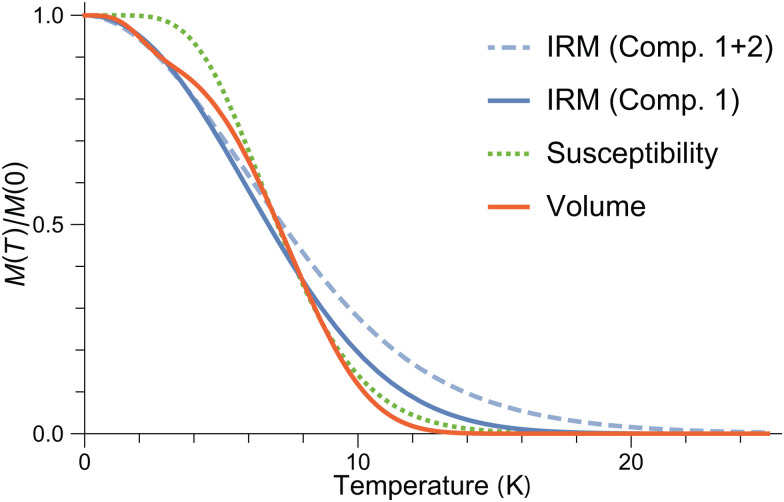
Cumulative blocking temperature distributions reconstructed from IRM measurements (Comp. 1 and Comp. 1 + 2), from low-field susceptibility measurements (Susceptibility), and from *E*_b_ = *KV* with *K* ≈ 19 kJ m^−3^ (Volume).

Another proof for the above conclusion is provided through the analysis of the relations existing between blocking temperature, magnetic moment, and anisotropy field distributions. In case of single-domain particles with a fixed spin configuration, these relations are established by the Néel–Arrhenius model (eqn (6)). We start with the normalized temperature dependence of the saturation remanent magnetization *M*_rs_ corresponding to the sum of the IRM components C1 and C2, which we attributed to Fh (Fig. 16). The normalized saturation remanent magnetization *M*_rs_(*T*)/*M*_rs_(0) of C1+2, coincides, by definition, with the integral of the blocking temperature distribution *f*_b_(*T*) of the Fh cores. Assuming the anisotropy constant *K* to be the same for all ferritin cores, one can expect from eqn (6) that *V* is proportional to *T*_b_, so that a rescaled version *cf*_*V*_(*cV*) of the volume distribution *f*_*V*_(*V*) obtained from TEM statistics (Fig. 1) will match *f*_b_(*T*) for a certain value of the proportionality constant *c* = *V*/*T*_b_. Indeed, a good match with the IRM component C1 is obtained using *c* ≈ 20 nm^3^ K^−1^ (Fig. 16). The match is less good if one considers C1+2, because of the wider composite blocking temperature distribution. It is therefore possible that C1 and C2 represent two distinct groups of ferritin cores with a narrower and a wider anisotropy distribution, respectively, so that the narrow anisotropy distribution of C1 is well approximated by a mean *K* value. In this case, *K* = *c*^−1^*k*_B_ ln *t*/*τ*_0_ ≈ 19 kJ m^−3^ is obtained for C1 using *τ*_0_ = 10^−11^ s^84^ and *t* = 5 s for the IRM measurement time. Comparable values have been reported in the literature, *e.g.*, *K* ∼ 25 kJ m^−3^ for horse spleen ferritin,^38^ and *K* ∼ 17 kJ m^−3^ for bacterial ferrihydrite.^69^ A slightly worse match is obtained when a similar rescaling procedure is applied to the distribution of *E*_b_ derived from susceptibility measurements (Fig. 16). In this case, the lack of contributions at temperatures smaller than 4 K reflects the shape of the gamma distribution used to fit the measurements. The relatively well-constrained value of the bulk anisotropy *K* suggests that most Fe ions are embedded in a crystalline structure.

Our surface-spin model of ferritin (Section 3.4) predicts that the distribution of ln *m*, which we assumed to be Gaussian when fitting *M*(*B*) curves, is given by *g*_m_ = *g*_*κμη*_ * *g*_*V*^2/3^_, where *g*_*κμη*_ and *g*_*V*^2/3^_ are the distributions of ln(*κ*_s_*μ*_Fe_*η*_s_) and ln *V*^2/3^, respectively, and “*” is the convolution operator. Because *κ*_s_ and *μ*_Fe_ are fixed quantities, and the standard deviation *σ* ≈ 0.08 of ln *V*^2/3^ is much smaller than *σ* ≈ 0.96 for ln *m*, the moment distribution is controlled mainly by the degree of spin canting *η*_s_ = sin *ε*_s_, and thus, by the surface anisotropy. The distribution *g*_*η*_ of *η*_s_ obtained from the deconvolution of the empirical moment and volume distributions (Fig. S22, ESI†) is almost perfectly Gaussian, owing to the fact that *g*_m_ used in the Langevin model was a normal distribution. About 96% of the distribution is comprised between *η*_s_ ≈ 1% and ∼50%, which is a reasonable upper limit for the alignment of surface spins.

**Fig. 17:**
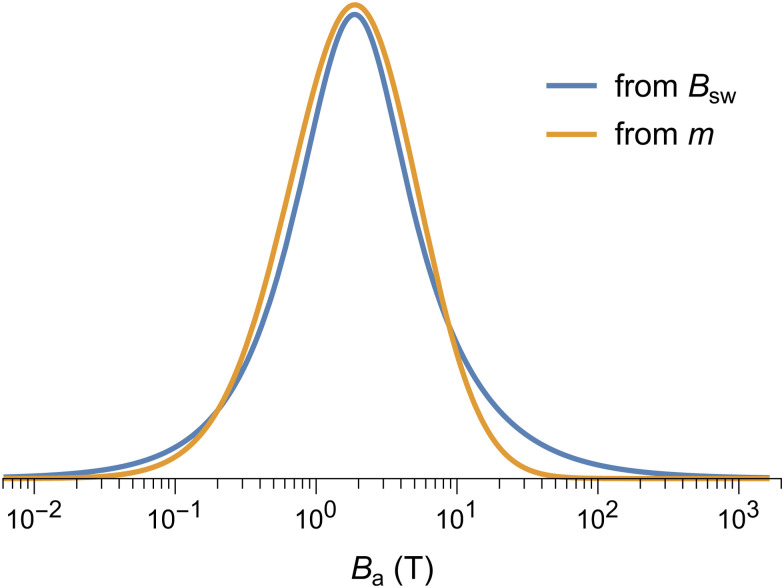
Comparison between a direct estimate of the anisotropy field distribution obtained from *B*_a_ = 1.8*B*_sw_, with *B*_sw_ being the switching field distribution of the IRM Component 1 + 2 at 3 K, and a reconstruction based on the magnetic moment model explained in the text.

Finally, we compare the anisotropy-field distributions obtained from IRM acquisition curves and from the magnetic moment and volume distributions, respectively. In the first case, we identify the distribution of *B*_a_ with the IRM coercivity component C1+2, using the relation *B*_sw_ = *ζB*_a_ between *B*_a_ and the switching field of single-domain particles well below the blocking temperatures. In the case of randomly oriented particles with uniaxial anisotropy,^96^*ζ* ≈ 0.524. In the second case, the distribution of ln*B*_a_ is given by *g*_a_ = *g*_2*K*/*κμ*_ * *g*_*V*1/3_ * *g*_*η*−1_ (Section 3.4). A good match between the two estimates is obtained using *B*_a_ = 1.8*B*_sw_ (Fig. 17). The corresponding *ζ* ≈ 0.56 is slightly larger than the value expected for randomly oriented uniaxial single-domain particles and might be explained by a single-particle anisotropy with a small degree of non-uniaxiality.

In summary, the modified Langevin model used to fit *M*(*B*) curves (Section 4.4), the lack of a spin-flop transition (Section 3.2), the magnetic moment distribution (Sections 3.3 and 3.4), the coercivity distribution (Section 4.5) and the compatibility of volume, magnetic moment, blocking temperature, and anisotropy field distributions with the Néel–Arrhenius model (Section 3.4, Fig. 17) point, altogether, to a model for the spin structure of ferritin cores where the magnetic moment arises from surface-spin canting, rather than randomly distributed defects in the AF sublattices. The ferritin cores behave as single-domain particles with uniaxial magnetocrystalline anisotropy. The wide distribution of *m* is explained by the variability of spin configurations deviating from the two-sublattice AF model, rather than the volume distribution. Despite the strong heterogeneity of magnetic configurations, all ferritin cores appear to share a well-defined magnetic anisotropy constant *K*. This is consistent with a model where spins are rigidly coupled and switching occurs by uniform rotation, so that most of the work necessary to overcome the energy barrier originates from the intrinsic anisotropy of the AF sublattices. Finally, the wide distribution of switching fields, from ∼0.03 to >30 T, is also a direct consequence of this model, which predicts *B*_sw_ ∝ *m*^−1^ for AF particles with a spontaneous moment *m*. In particular, *m* → 0 yield extremely large switching fields, which are limited only by the sublattice exchange field *B*_E_.

The above model explains magnetometry measurements almost completely. As discussed in Section 4.5 only ∼4% of the total remanent magnetization is carried by phases that cannot be associated with a Fh core composition. *M*(*B*) curves contain a paramagnetic contribution that is carried by a small fraction (∼6.5%, Table 2) of mononuclear Fe^3+^ atoms. This fraction of ∼6.5% is obtained from the ratio *αμ*_m_/*η* between the saturation magnetization of these atoms and the Fh cores in eqn (12). The last magnetic parameter that describes the ferritin cores is the non-paramagnetic susceptibility *χ* that contributes with a linear term *χB* to the *M*(*B*) curves. This term originates from the field-induced spin canting of surface spins and of the inner spins with AF order. The estimate of *χ* at 50 K obtained from *M*(*B*) curves fits with eqn (12) is ∼1.4 times larger than the value expected from the simple model of spin-canted AF nanoparticles discussed in Section 5.3.2 (Table 2). Measurements of horse-spleen ferritin up to 50 T show that the slope of *M*(*B*) curves measured at 4.2 K continues to decrease up to ∼35 T, before merging with the superantiferromagnetic trend.^39^ It is therefore possible that the excess susceptibility in our fits of *M*(*B*) curves limited to a maximum field of 7 T is caused by the linear approximation of a small residual curvature due to superparamagnetic contributions that saturate in much larger fields. These contributions might require a magnetic moment distribution with a heavier left tail associated with much smaller Fh cores or isolated core fragments, as suggested by the measured volume distribution (Fig. 1).

**Table 2:** Magnetic properties of ferritin cores derived from magnetometry (M) and EPR (E) measurements. *μ*—spontaneous magnetic moment; *σ*_*μ*_—standard deviation of the distribution of ln *μ*; *B*_a_—anisotropy field; *σ*_B_—standard deviation of the distribution of ln *B*_a_; *B*_c_—coercive field of hysteresis; *E*_b_—energy barrier; *σ*_E_—standard deviation of the distribution of ln *E*_b_; *K* uniaxial anisotropy constant; *σ*_*K*_—standard deviation of the distribution of ln *K*; *M*_p_/*M*_Fh_—paramagnetic saturation magnetization, normalized by the saturation magnetization of the superparamagnetic contribution; *N*_p_/*N*_Fh_—number of paramagnetic Fe atoms, normalized by number of superparamagnetic atoms of ferritin core; *χ*/*χ*_c_—non-paramagnetic susceptibility in *M*(*B*) fits with eqn (12), normalized by the value expected from superparamagnetic particles with spin canting moments (Section 3.2)

Property	From magnetometry	From EPR
*μ*/*μ*_B_	337^a^	200, ∼1000^m^
*σ*_*μ*_	0.947^b^	—
*B*_a_ (T)	1.9^c^	0.1–0.3^n^
*σ*_B_	1.44^d^	—
*B*_c_ (T)	0.08^e^	—
*E*_b_/*k*_B_	227^f^	—
*σ*_E_	0.679^g^	—
*K* (kJ m^−3^)	19^h^	—
*σ*_*K*_	0.417^i^	—
*M*_p_/*M*_Fh_	2.39^j^	
*N*_p_/*N*_Fh_	0.0645^k^	0.0036^o^
*χ*/*χ*_c_	∼1.4^l^	—

a Mean, Fig. 12c, 0 K.

b Langevin fit.

c Median of C1 + 2, 3 K.

d C1 + 2, 3 K.

e Hysteresis.

f *T*_b_ of C1 + 2.

g *T*_b_ of C1 + 2.

h Eqn (6).

i Eqn (6).

j *η*/*αμ*_m_ in eqn (12).

k *η*〈*m*〉/*αμ*_m_*Nμ*_Fe_ in eqn (12).

l 50 K, Fig. 5b.

m For *n* = 10, 30, *i.e.*, *S* = 100 and *S* = 300, respectively.

n E1 & E2, 20 K.

o For *S* = 300; for *S* = 100 *N*_p_/*N*_Fh_ = 4 × 10^−4^ From ESI, section “Ferritin core and mononuclear Fe(iii) EPR intensities”.

### 5.3 Comparison between EPR and magnetometry

#### 5.3.1 Blocking temperature from EPR and magnetometry

According to the Results section (Fig. 7b), the maximum EPR blocking temperature, *T*_b_ ∼ 100 K, is in agreement with values reported for horse-spleen ferritin.^13,43^ The blocking temperature values obtained from magnetometry, *e.g.*, from the ZFC susceptibility maximum at ∼10.5 K, are much lower than those obtained from EPR, owing to the drastically different characteristic timescales *t*_ZFC_ ∼ 100 s and *t*_EPR_ ∼ 0.1 ns. From the Néel–Arrhenius law15
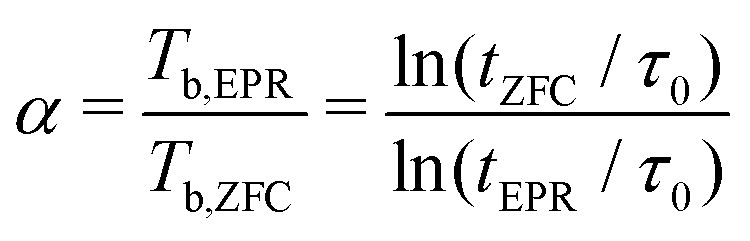
the attempt time estimate *τ*_0_ = (*t*^*α*^_EPR_/*t*_ZFC_)^1/(*α*−1)^ = 3.9 ps is obtained, in good agreement with the range of values reported for ferritin^107^ and other AF nanoparticles of similar size.^108^

#### 5.3.2 Magnetic moment and anisotropy field from EPR and magnetometry

**Fig. 18:**
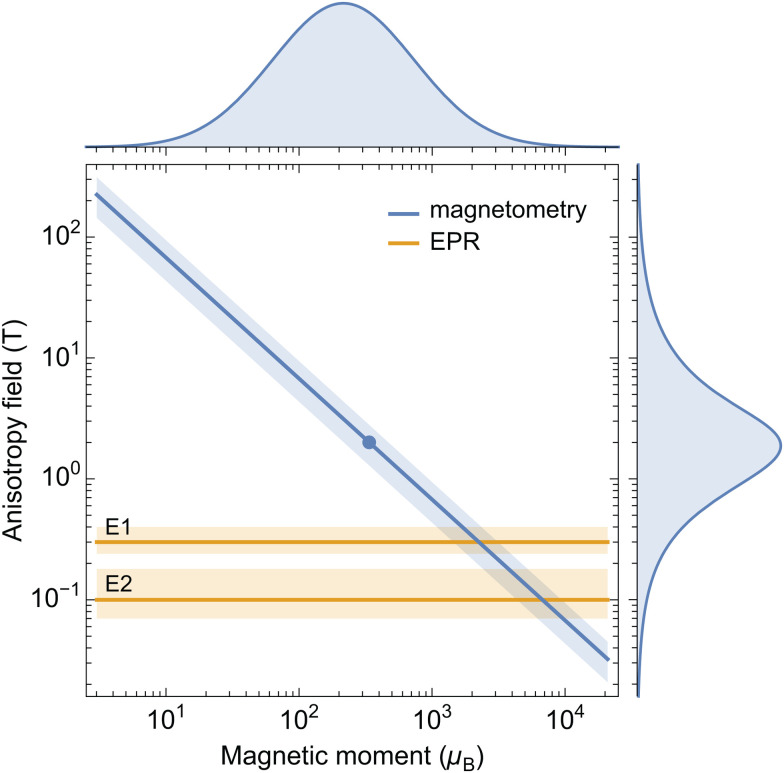
Anisotropy field (*B*_a_) *vs.* magnetic moment (*μ*) from EPR (components E1 and E2 at 20 K) and from magnetometry. Shading around the magnetometry and EPR trends correspond to the quartiles of *T*_B_ distribution (Fig. 16) and to the range of EPR parameters yielding similar simulated spectra, respectively. The dot represents the averages of *B*_a_ and *μ* obtained from magnetometry. The distributions of *μ* and *B*_a_ obtained from magnetometry are plotted on the corresponding axes.

As discussed above, it is not possible to perform a full inversion of EPR spectra to resolve the magnetic moment and anisotropy-field distributions needed for a quantitative comparison with magnetometry results. The amount of paramagnetic *vs.* ferrihydrite-like phases by EPR is obtained from the ratio of the intensities of the *g* = 4.3 signal and the broad EPR signal, resulting in 0.4% of mononuclear Fe(iii) atoms (Table 2). The difference with respect to the magnetometry results (6.5%) is dominated, from the EPR side, by the uncertainty in the spin quantum number *S* of the ferritin core (see ESI,† section “Ferritin core and mononuclear Fe(iii) EPR intensities”). Also, only the mononuclear Fe(iii) signal is taken into account, leading to a possible underestimation of the paramagnetic contribution from EPR. From the magnetometry side, small clusters of iron ions with a superparamagnetic contribution similar to the Brillouin function used to model the paramagnetic phase might lead to an overestimation of the paramagnetic contribution, whereas in EPR such clusters may escape detection due to broadening or unfavorable relaxation properties. The existence of small iron clusters or incomplete ferritin cores with a much smaller magnetic moment is supported by the <4.5 nm tail of the core-size distribution obtained from TEM (Fig. 1). In view of the above mentioned uncertainties in the determination of para- and superparamagnetic contributions, the agreement between EPR and magnetometry can be considered satisfactory. Nevertheless, it is still possible to verify the compatibility of the two EPR components E1 and E2 with magnetometric parameters. For this purpose, we plot the estimated *μ* and *B*_a_ ranges obtained from both techniques (Fig. 18). As discussed in Section 5.3, an inverse relationship of the form *B*_a_ = 2*E*_b_/*m* holds between the anisotropy fields and magnetic moments of individual ferritin cores. Because the distribution of *E*_b_ is much narrower than those of *B*_a_ and *μ*, possible magnetic moment and anisotropy field combinations of individual cores lie close to a line with slope −1 in the bilogarithmic plot of Fig. 18. On the other hand, simulated EPR spectra sharing the same value of *S*⋅*D* are characterized by almost identical shapes (Fig. S10, ESI†), owing to the scaling rules of Fittipaldi *et al.*^50,51^ Because *S*⋅*D* = *gμ*_B_*B*_a_/2 (eqn (9)), EPR spectra with same *B*_a_ are indistinguishable, which means that the magnetic moment is totally unconstrained. Accordingly, the EPR components E1 and E2 define two horizontal lines in Fig. 18. If these components are interpreted as the discrete representation of a broad distribution of EPR parameters, the corresponding *B*_a_ values (Table 1) can be interpreted as two discrete samples of a broad anisotropy field distribution.

The intersection between the constraints plotted in Fig. 18 gives a rough estimate of the anisotropy fields and magnetic moments probed by EPR, which are centered around *B*_a_ ≈ 0.2 T and *μ* ≈ 4000*μ*_B_, respectively. This combination of parameters is off by a factor ∼10 with respect to mean values obtained from magnetometry (*B*_a_ ≈ 2 T and *μ* ≈ 337*μ*_B_). This discrepancy originates from the ∼10 times smaller anisotropy fields of E1 and E2. A possible explanation for this difference is discussed in the next section.

#### 5.3.3 Origin of the lower EPR anisotropy field

As discussed in Section 5.3.2, *B*_a_ estimates obtained from EPR spectra are about one order of magnitude smaller than the median *B*_a_ obtained from IRM acquisition curves, meaning that the two measurements probe distinct processes related to particle anisotropy. In the case of IRM acquisition curves, *B*_a_ is probed through the switching field *B*_sw_, defined as the field required to reverse the remanent magnetic moment of a single-domain particle. In the case of a sufficiently rigid coupling between spins, particles with uniaxial anisotropy possess only two antiparallel magnetic states, and *B*_sw_ is the field in which a transition occurs between these two states. Coherent rotation,^81^ fanning,^109^ and curling,^110^ are just some examples of such two-state models of single-domain particles. The type of reversal mechanism determines the dependence of *B*_sw_ on the angle between anisotropy axis and applied field, and thus the relation between the bulk *B*_sw_ of IRM acquisition curves and *B*_a_, *e.g. B*_sw_ ≈ 0.524*B*_a_ in case of coherent rotation. Regardless of the reversal mechanism, transitions between the two magnetic states in IRM and EPR measurements are governed by the same equation for the particle energy: *E* = −*B*_a_*μ*(**e**·**n**)^2^/2 − *Bμ*(**ẑ**·**n**), where **e** and **n** are the unit vectors parallel to the easy axis and the magnetic moment respectively, and the applied field is parallel to **ẑ**. Accordingly, the same *B*_a_ is sensed by the two methods.

Surface anisotropy complicates the description of the particle magnetization by introducing intermediate magnetic states along the path that produces a field-induced reversal of the bulk magnetic moment.^111^ In practice, the switching process begins by reversing discrete groups of surface spins in increasingly large fields, until reversal of the internal core spins completes the process. As a result, single-particle hysteresis contains several discrete magnetization jumps corresponding to partial reversals,^111^ instead of the single jump at *B*_sw_ of two-state particles. The way multistep magnetic moment reversals are recorded depends on the measurement protocol. In the case of IRM acquisition curves, partial reversals of surface spins will not be recorded, because the strong exchange coupling with the internal core spins recovers the initial spontaneous moment as soon as the field is removed. Accordingly, switching occurs only in a field that is sufficiently strong to reverse the internal core spins. As shown in Section 5.2, this field is given by *B*_a_ = 2*KV*/*m*, where *K* is the anisotropy constant of the AF-coupled core spins. On the other hand, the entire sequence of partial reversals is recorded by the hysteresis loop (Fig. 10). As a result, the coercive field of hysteresis, *B*_c_ ≈ 0.1 T, is ∼10 times smaller than the median switching field *B*_1/2_ ≈ 1 T obtained from IRM curves. For comparison, randomly oriented Stoner–Wohlfarth particles^112^ are characterized by *B*_1/2_/*B*_c_ ≈ 1.2. Like hysteresis, EPR spectra are expected to record all transitions between magnetic states, and therefore also partial reversals occurring at lower fields. The apparent anisotropy field of EPR spectra is therefore more similar to the coercive field of hysteresis than the field required for a complete reversal. This explains why a direct comparison of EPR and magnetometry data, as in Fig. 18, does not work.

The existence of multiple magnetic states for the ferritin cores questions the applicability of the Langevin model for fitting *M*(*B*) curves, since particles in the superparamagnetic state can undergo thermally activated transitions between any pair of states. Intermediate states with partially reversed surface spins have higher energies than the ground states, and the whole sequence of reversal steps is represented by a path in a multidimensional energy landscape, which connects a series of local minima distributed along a “valley” running from a ground state to its opposite.^113^ The presence of local minima along this valley raises the probability of finding particles with these discrete intermediate states, compared to the continuous transition between ground states of particles with no intermediate states. Close to the blocking temperature, the probability of magnetic configurations different from ground states is negligibly small, and intermediate states do not play any role. At higher temperatures, the existence of multiple states with different energy levels has a similar effect as multiaxial anisotropy, and deviations from the Langevin law can be expected to be smaller than those produced by uniaxial anisotropy (Section 3.2), owing to the smaller switching fields associated with transitions between intermediate states. Because the effect of uniaxial anisotropy is small in the temperature range of our *M*(*B*) measurements (Fig. 4), the Langevin model is expected to be a good representation of the equilibrium magnetization even in the case of particles whose magnetic moment is not reversed in a single step. Consequently, the Langevin model, as applied in the present context is a good approximation for the magnetic properties of ferritin.

## 6 Conclusions

In this work, we have probed the magnetic response of a sample of human liver ferritin with a broad set of available experimental techniques and at different excitation frequencies, sparsely covering the DC-to-9 GHz interval. While magnetic analyses of ferritin are not new, our synergetic comparison of magnetometry and electron paramagnetic resonance offers, for the first time, an in-depth description of ferritins spin behavior. Our methods reconcile recurring discrepancies on the origin of the magnetic moment in ferritin on the one hand, while offering a quantitative approach to the challenging determination of spin Hamiltonian components emerging from EPR data fitting on the other hand, over a temperature range extending close to the DC-blocking temperature.

With regard to EPR data, the broad and seemingly featureless nature of the spectra can be well captured by at least two components, with *S* and *D* parameters returning an anisotropy field in the 0.1–0.6 T range, and an *S* parameter above 50. These two components most likely represent a broad distribution of magnetic properties. While the magnetic moment can only indirectly be derived from the EPR data, the technique identifies also paramagnetic contributions, providing information complementary to magnetometry.

Moreover, a more complete understanding of the magnetometry data of ferritin, which has been the subject of extensive debate, can be achieved with IRM acquisition data. IRM acquisition curves reveal (i) the mineral composition of the protein core, which complements the information from other methods, such as energy-resolved electron microscopy; (ii) the blocking temperature distribution, in reasonable agreement with the particle volume distribution and the assumption of a single anisotropy constant; and (iii) the existence of a negative exchange field between the dominant antiferromagnetic phase and a minor ferrimagnetic phase within the same core. This exchange interaction prevents the magnetic moments of the two phases to display independent superparamagnetic behaviours.

From a new theoretical description of the induced magnetization data we conclude that the magnetic moment of ferritin is controlled mainly by spin canting caused by surface anisotropy. The wide distribution of magnetic moments can be explained by the strong heterogeneity of surface-spin configurations, to which the EPR spectrum seems to be particularly sensitive. Because of the inverse proportionality relation between magnetic moment and anisotropy field imposed by the Néel–Arrhenius law, the coercivity of ferritin is also represented by a broad distribution.

## Author contributions

LB devised the experiments, analyzed the data, and reviewed the manuscript. JLM devised EPR measurements, did the experiments and analysis, wrote parts of the manuscript, HvdZ read and reviewed the manuscript, VC did experiments and analysis and corrected the manuscript, AL did magnetometry measurements, RE implemented theoretical models for magnetometry analysis, analyzed the data, wrote parts of the manuscript and revised it. MH supervised the project, did part of the EPR analysis, and writing of the manuscript.

## Conflicts of interest

There are no conflicts to declare.

## Supplementary Material

CP-025-D3CP01358H-s001
