# Supplementary material for: In-depth magnetometry and EPR analysis of the spin structure of human-liver ferritin: from DC to 9 GHz
Source: Phys Chem Chem Phys. 2023 Oct 9;25(40):27694–717. doi: 10.1039/d3cp01358h (PMC10583656; doi:10.1039/d3cp01358h)
Supplement: CP-025-D3CP01358H-s001 [file CP-025-D3CP01358H-s001.pdf]

Electronic Supplementary Material (ESI) for Physical Chemistry Chemical Physics.
This journal is © the Owner Societies 2023

In-depth Magnetometry and EPR Analysis of the Spin Structure of Human-Liver Ferritin: from DC to 9 GHz

Lucia Bossoni,[†,⊥] Jacqueline A. Labra-Muñoz,[‡,¶,⊥] Herre S. J. van der Zant,[¶] Vera Čaluković,[‡] Anton Lefering,[§] Ramon Egli,[*,‖] and Martina Huber[*,‡]

†*C. J. Gorter Center for High Field MRI, Department of Radiology, Leiden University Medical Center*

‡*Department of Physics, Huygens-Kamerlingh Onnes Laboratory, Leiden University, 2300 RA Leiden, the Netherlands*

¶*Kavli Institute of Nanoscience, Delft University of Technology, 2628 CJ Delft, the Netherlands*

§*Reactor Institute, Delft University of Technology, Delft, The Netherlands*

‖*ZAMG Central Institute for Meteorology and Geodynamics, Howe Warte 38, 1190 Vienna, Austria*

⊥*These authors contributed equally*

E-mail: ramon.egli@geosphere.at; huber@physics.leidenuniv.nl

Contents

TEM description 3

Ferritin purity assessment 4

EPR baseline correction 5

EPR simulation with one component 8

EPR simulations, from 20 K to 210 K 9

EPR simulations of individual components 10

EPR simulations parameters 11

EPR simulations with different scaling factors 13

EPR S-D inverse compensation 14

Sensitivity of EPR parameters 15

Ferritin core and mononuclear Fe(III) EPR intensities 16

EPR Model Simulations 18

EPR alternative fitting approach 19

Equilibrium magnetization models 22

List of Symbols and Acronyms 29

TEM description

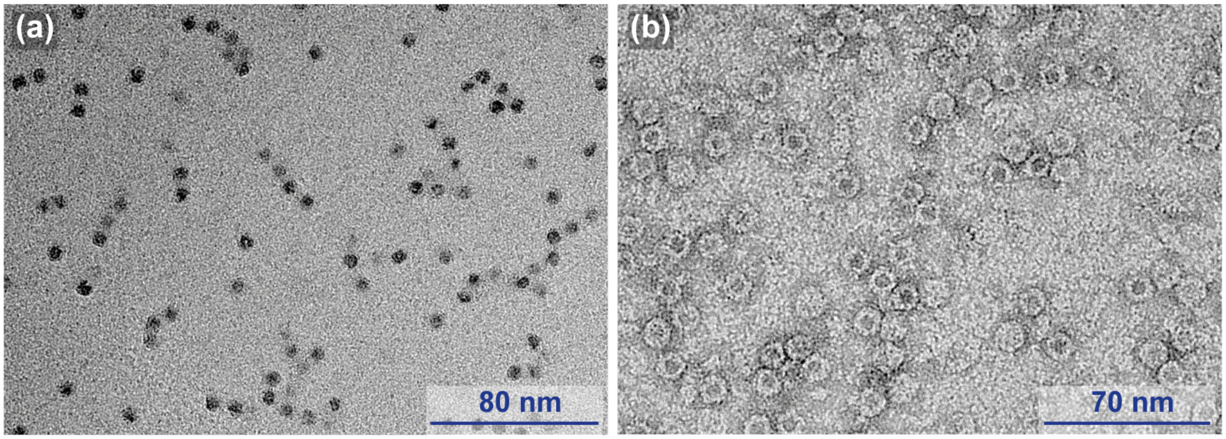

Figure S1: Transmission electron microscopy (TEM) characterization of human-liver ferritin. (a) TEM image of human-liver ferritin cores. (b) Stained-SEM image of human-liver ferritin particles allowing the protein shell visualization. The images were acquired with the JEOL JEM-1400 series 120 kV Transmission Electron Microscope.

Regionprops is a function implemented in MATLAB. In particular, we used the regionprops function implemented for MATLAB2019a. This function detects connected components in an image (objects). For each object, the total area, centroids, and many other properties can be extracted. For detecting the ferritin cores, we first binarized the images to enhance the contrast between the ferritin cores and the image background, then the center and radii of all the circular objects that are detected in the image are calculated. To separate the ferritin cores from other possible circular objects a threshold parameter (in the binarization preprocess) is needed to prevent the detection of false positives and minimize the false negatives (the undetected cores). In the image below, encircled in red are all the individual ferritin cores that were detected with the algorithm. Encircled in light blue is an example of a false negative. 7.7% of ferritin cores are undetected. The diameter estimation has a standard deviation of $\pm$ 0.35 nm, which is the uncertainty. A limitation of this algorithm is the assumption that the ferritin cores are spherical, which is not necessarily the case. Additionally, ferritin cores that are formed by two sub-nanoparticles inside one ferritin will be detected as two different ferritin cores.

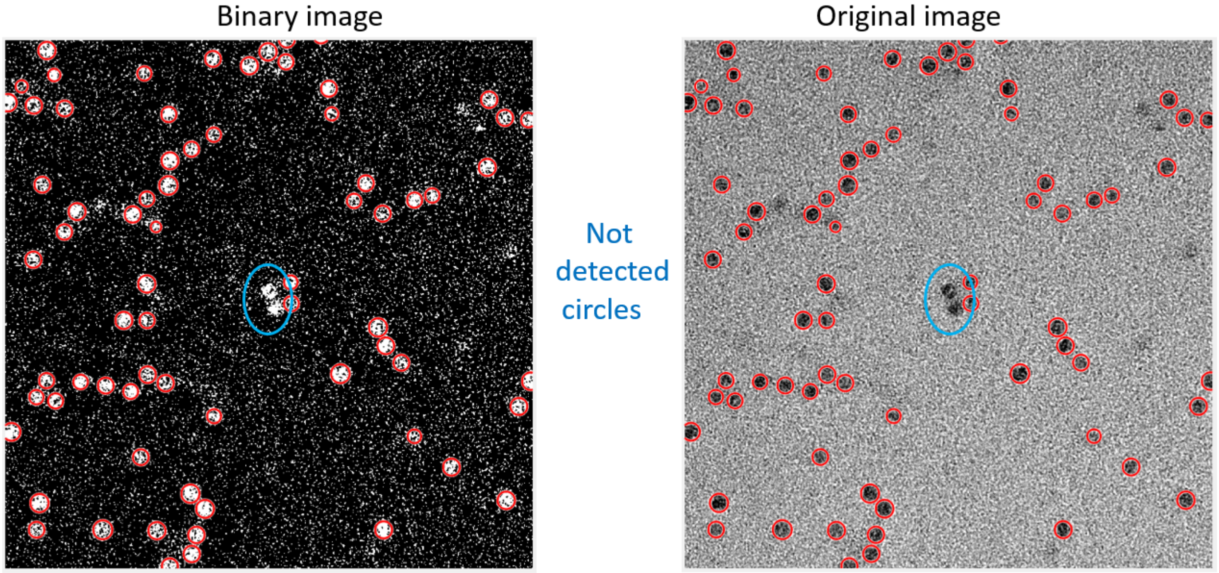

Figure S2: Transmission electron microscopy (TEM) characterization of human-liver ferritin. (a) Binarized TEm image. (b) Original image (before binarization). Red circles indicate detected ferritin cores. Blue circles indicate undetected cores.

Ferritin purity assessment

NativeMark Unstained Protein Standard (Cat. No. LC0725) was obtained from Life Technologies Corporation. The purity and the homogeneity of the protein were assessed by a 7.5% non-denaturing polyacrylamide gels electrophoresis. Gel electrophoresis was run at 4°C and 90 V until samples reached the running gel, then the voltage was increased to 160 V for 3 hours. After electrophoresis, Coomassie blue staining was used to stain proteins. For iron staining, the gel was immersed in Prussian blue staining solution, which was freshly prepared by mixing 2% $K_4Fe(CN)_6$ and 2% HCl (1g Potassium ferrocyanide + 47,3 ml H_2O + 2,7 ml HCl) for 1h at RT, then 1h in water, changing water every 15-20 min. Finally, the gel was incubated in a solution containing 0.025% DAB (3,3'-Diaminobenzidine-Sigma) and 0.05% H2O2 in 1X TBS [12,5mg DAB resuspended in 500 µl DMSO, added to 50 ml TBS 1x, 75-180 μl H_2O_2 added just before incubation] for 15-30 minutes at RT to enhance the signal.

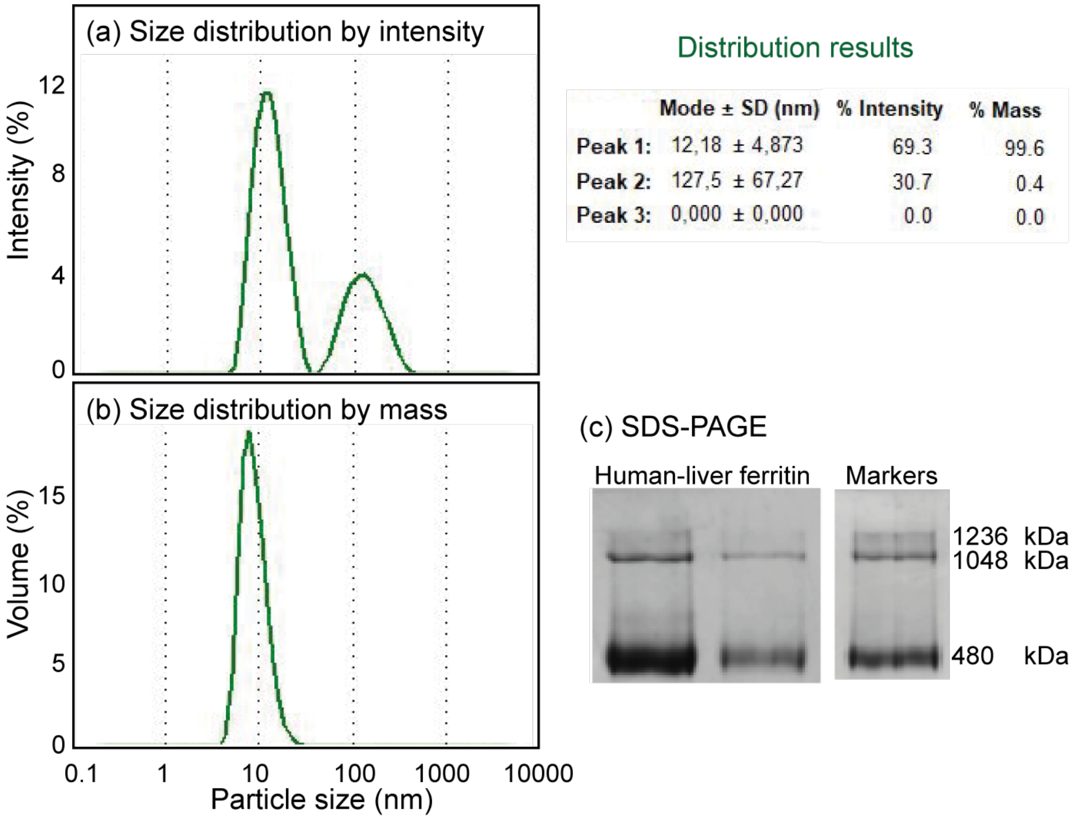

Figure S3: Purity assessment of human-liver ferritin. (a,b) Dynamic light scattering showing the particle size (hydrodynamic diameter) distribution: one main Gaussian peak with μ=12.2 nm and σ=4.5 nm. (c) Non-denaturing polyacrylamide gel electrophoresis of human-liver ferritin (HSF) stained with Coomassie. The strong band of 500 kDa agrees with ferritin monomers (24-mer protein) while the weaker bands of 1.048 and 1.236 kDa correspond to ferritin multimers (dimers/trimers) or aggregates of ferritin protein. Markers refer to NativeMark Unstained Protein Standard (Cat. No. LC0725).

EPR baseline correction

The baseline correction of the spectra was performed by a home-made script implemented in Matlab 2019a, which automatically looks for a linear transformation (Fig. S4a), such that the slope of the baseline correction is the optimal value by which the high magnetic field values of the second integral of the spectra reach a plateau-like region (Fig. S4c, which is equivalent to having a zero first integral value at the highest field values (Fig. S4b).

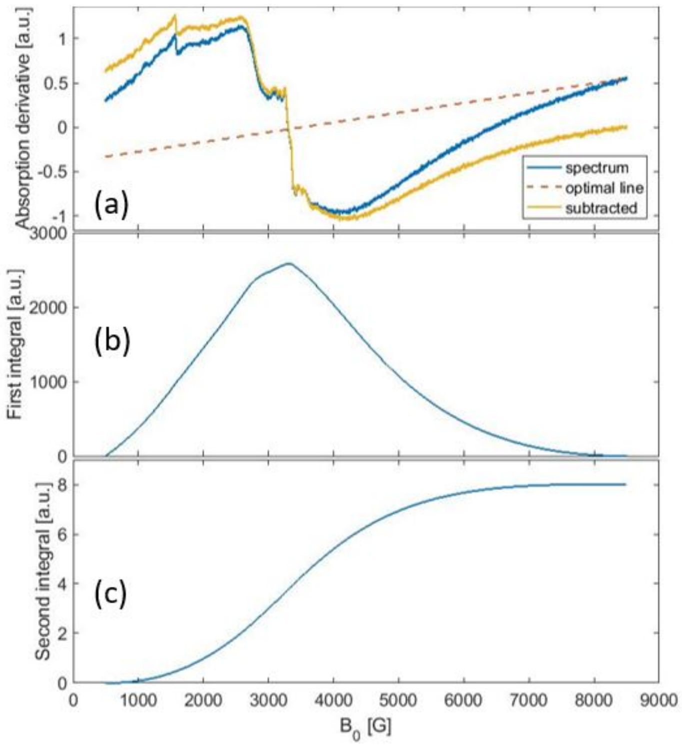

Figure S4: Automatic baseline correction algorithm. (a) The experimental spectrum (in blue), the optimal line (red dotted line), and the transformed spectrum (yellow curve). (b) The first integral of the baseline-corrected spectrum. Its high field values reach zero. (c) The second integral of the baseline-corrected spectrum. The high field values reach a plateau-like region.

ICP-MS characterization

Materials:

Nitric acid (65%, Suprapur®, Merck) was used in the digestion process, and further diluted 1% nitric acid was used as a carrying solution throughout the ICP measurements. National Institute of Standards and Technology (NIST)-traceable 1000 mg/L elemental standards were used (TraceCERT®, Fluka) for the preparation of calibration and internal standards. Approximately 18 MΩ cm^{-1} water (MiliQ) was used in all sample preparation and analysis steps.

Instrumentation:

Calibration standards were prepared in a Secuflow fumehood (SCALA) to prevent contamination by atmospheric particulates. The standards and samples were analysed for trace elements using the NexION® 2000 (PerkinElmer) ICP-MS equipped with a concentric glass nebulizer and peltier-cooled glass spray chamber. An SC2 DX autosampler (PerkinElmer)

was connected to the ICP-MS for sample introduction. SyngistixTM Software for ICP-MS (v.2.5, PerkinElmer) was used for all data recording and processing.

Elemental Analysis:

Five trace elemental calibration standards for ICP-MS analysis were prepared using NIST-traceable 1000 mg/L Fe standard: 0, 1, 5, 20, 100 ug/L. 10 ug/L Rh and In were used as internal standard. Fe was analyzed in kinetic energy discrimination (KED) mode with 10% He gas to minimize polyatomic interferences. To check the calibration, samples were analyzed consisting of a blank measurement and a repeat measurement of one of the calibration standards. For the calibration curve only to be accepted as correlation coefficient (Cor.Coeff) was found higher than 0.999.

EPR simulation with one component

Figure S5 shows an example of one of the best fits obtained by considering a simulation with only one EPR component. It shows the discrepancy between the data and the model, indicating the need of two or more EPR components.

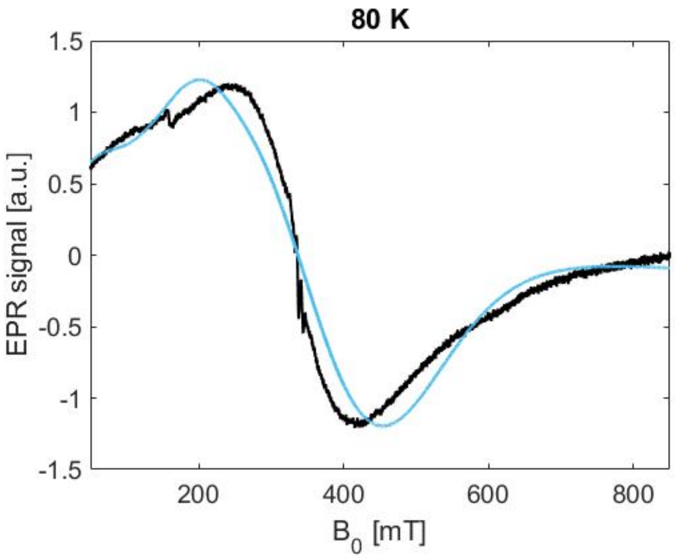

Figure S5: Simulation (light-blue line) of EPR spectrum (black line) with single component only (80 K). The simulation is performed with one spin component of S =15, g = 2.01, $D = -335$ MHz, and $H_{\mathrm{strain}} = 6500$ MHz.

EPR simulations, from 20 K to 210 K

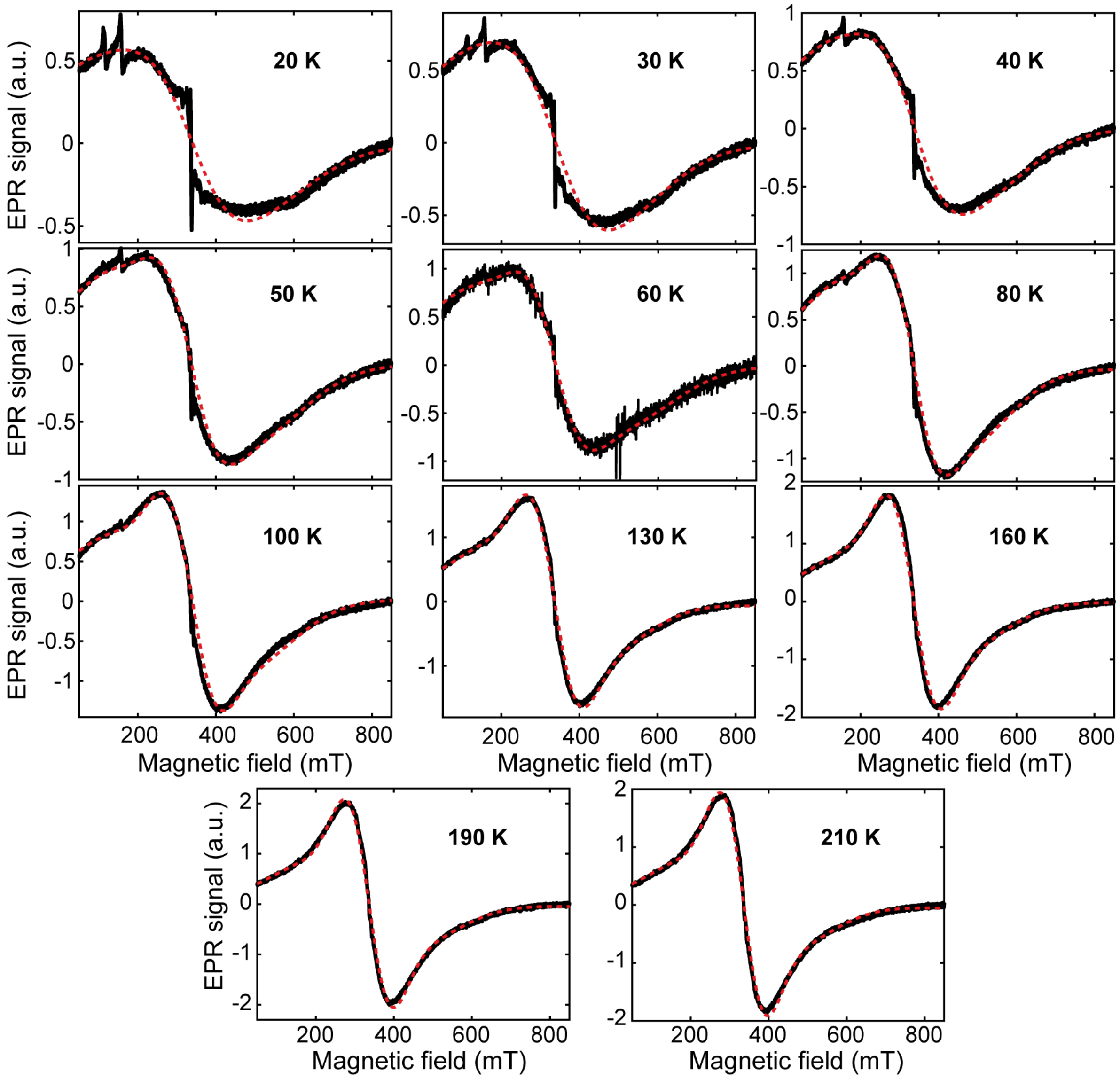

Figure S6: Simulations (red dotted lines) of EPR spectra (black lines) for all data recorded between 20 and 210 K. The simulations are performed with two spin systems, as described in the main text. The system parameters are shown in Table S1.

EPR simulations of individual components

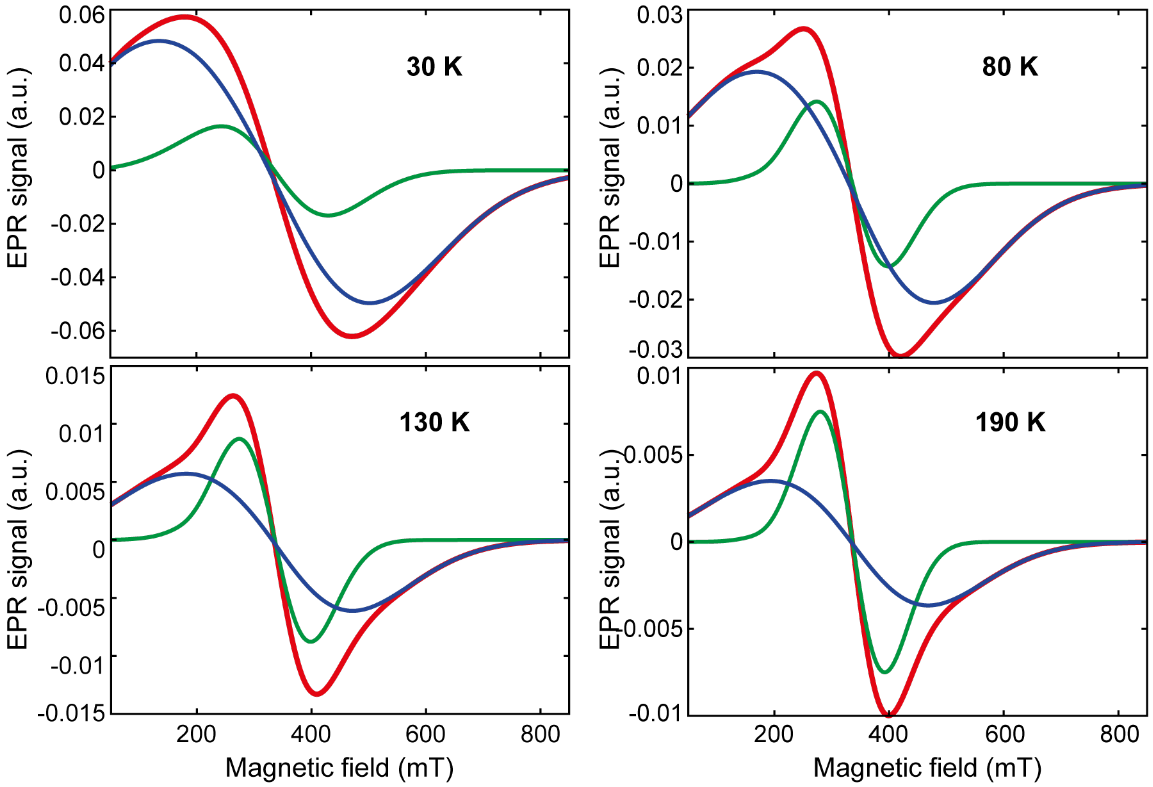

Figure S7: Total EPR simulations (red lines) performed at 30 K, 80 K, 130 K and 190 K. The simulations are performed with two spin systems, as described in the main text. The first system component is depicted in green. The second component is shown in blue.

EPR simulations parameters

Table S1: System parameters used for simulations of the EPR spectra. Simulation parameters: $S = 10$, D: axial zero-field splitting, H_{strain}: Gaussian broadening parameter. The parameters at 30 K, 80 K, 130 K, and 190 K were determined first.

	**Component 1**			**Component 2**		
T(K)	D (MHz)	H_{strain} (MHz)	Weight (%)	D (MHz)	H_{strain} (MHz)	Weight (%)
20	180	6000	7.7	450	11000	92.3
30	170	6000	9.1	400	10500	90.9
40	155	5500	9.5	370	10500	90.5
50	155	4600	10.0	370	10400	90.0
60	175	4500	10.5	300	11000	89.5
80	150	3900	11.1	370	8900	88.9
100	150	3900	15.4	370	8700	84.6
130	150	3900	22.2	350	8500	77.8
160	130	3900	24.4	350	8500	75.6
190	100	3600	26.3	250	8500	73.7
210	100	3500	26.3	250	8300	73.7

The simulation parameters used for all experimental spectra are shown in Table S1. The total spin for component 1 (E1) and component 2 (E2) is 10. Figure S8 shows the system-parameters temperature dependence for E1 and E2. A gentle slope is observed for the D values of both components, with a total decrease of 44% for both of them (Fig. S8a) as temperature increases. While the weight of E1 increases with the increase of the temperature, the weight of E2 decreases, although it remains preponderant over all the temperature range (see Fig. S8b). While decreasing the temperature, a gentle slope is observed for the H_{strain} (Gaussian broadening) of E1 and E2. However, around 50 K a sudden jump is observed in both cases.

The relative proportion of E1 decreases with temperature, from ∼93% at 20 K to ∼75% at 210 K (Fig. S8a). Finally, both components display similar temperature dependencies on D and H_{strain} (Fig. S8). The real temperature dependencies of E1 and E2 depend on the scaling factor given by the ratio between the unknown S_{real}, and $S = 10$ used for the model. For instance, $S_{\text{real}} = 100$ yields $n = 10$, and, according to the scaling laws of eq. 10 (see main article), the real temperature in Fig. S8 is 10 times larger than the modeled values.

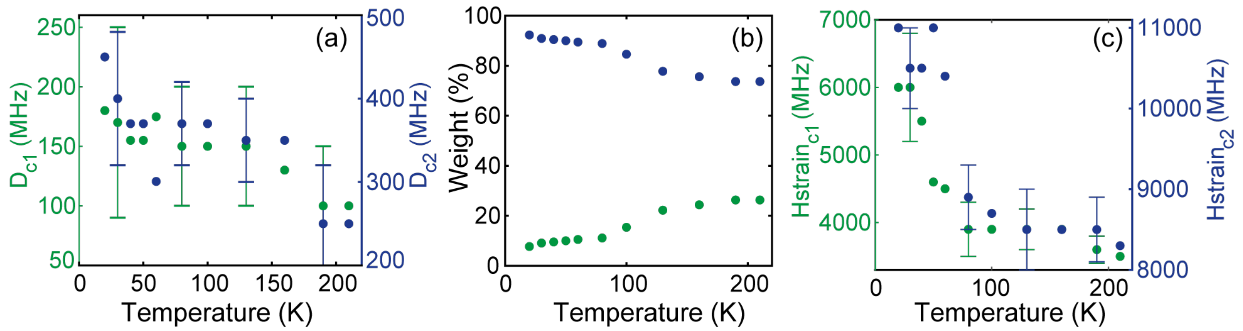

Figure S8: Simulation parameters as a function of temperature, for component 1 (green dots) and component 2 (blue dots). (a) D values. (b) Weights. (c) Broadening (H_{strain}). The parameters are given in Table S1.

Interpretation of D and H_{strain}

The temperature dependence of the H_{strain}, the Gaussian broadening of both components, see Fig. S S8, decreases with increasing temperature, revealing an averaging process that was previously[1] interpreted as anisotropy melting. We also observe a slight, though barely significant trend of D towards smaller values with increasing temperatures. Such a trend could also be a reflection of a reduction of the anisotropy of the ferritin core by some thermal averaging process. That the trend in D is not very pronounced suggests that the main effect of the anisotropy melting reflects itself in the reduction of the broadening parameter of the line, the H_{strain} parameter, in the simulation model we employ.

EPR simulations with different scaling factors

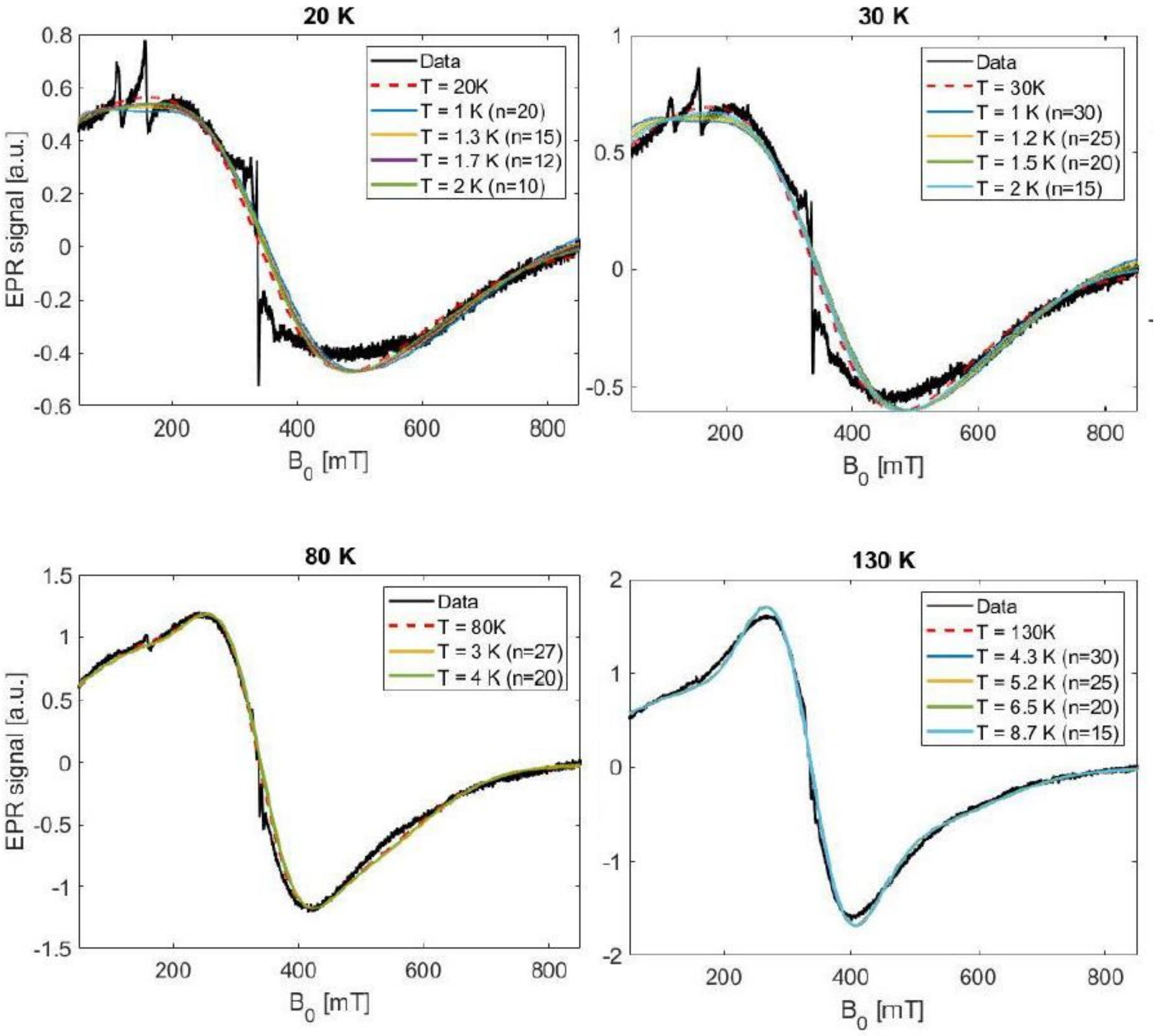

Figure S9: Simulations (colored lines) of EPR spectra (black lines) recorded at 20 K, 30 K, 80 K, and 130 K. The simulations are performed with two spin systems using different scaling factors (n). The remaining parameters are shown in Table S1. For the entire temperature range, the line shape presents negligible changes, confirming the scaling method as a suitable candidate to reproduce and study the EPR line shape of high-spin systems.

EPR S-D inverse compensation

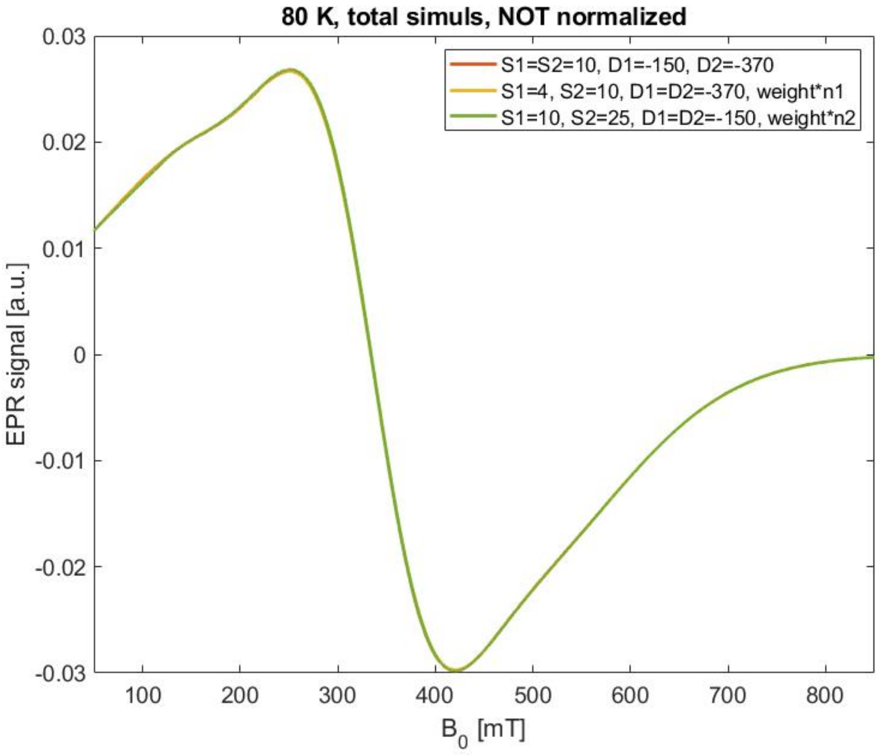

Figure S10: Simulations of EPR spectra performed at 80 K. The original simulation is shown in red. The simulations obtained by considering both components with equal D, The yellow curve is obtained by simulating both components with equal D.

The parameters S and D are inversely related. That means, an increase (decrease) of S of one of the components followed by a decrease (increase) of D of the same component by the same amount, results in a simulation with the same lineshape as the original simulation. It's important to mention that a correction in the weight of the component needs to be applied to preserve the same simulation amplitude. The correcting factor for components 1 (n1) and component 2 (n2) is defined by

$$\mathrm{n1} = \frac{S1_{\mathrm{orig}} \cdot (S1_{\mathrm{orig}} + 1)}{S1_{\mathrm{new}} \cdot (S1_{\mathrm{new}} + 1)} = \frac{10 \cdot 11}{4 \cdot 5} = 5.5, \tag{1}$$

$$\mathrm{n2} = \frac{S2_{\mathrm{orig}} \cdot (S2_{\mathrm{orig}} + 1)}{S2_{\mathrm{new}} \cdot (S2_{\mathrm{new}} + 1)} = \frac{10 \cdot 11}{25 \cdot 26} = 0.1692, \tag{2}$$

where SX_{orig} refers to the S used in the original simulations and SX_{new} to the change in S that is being applied and will result in the opposite change in D, by the same factor. $X = 1, 2$ refers to EPR components 1 and 2, respectively.

Sensitivity of EPR parameters

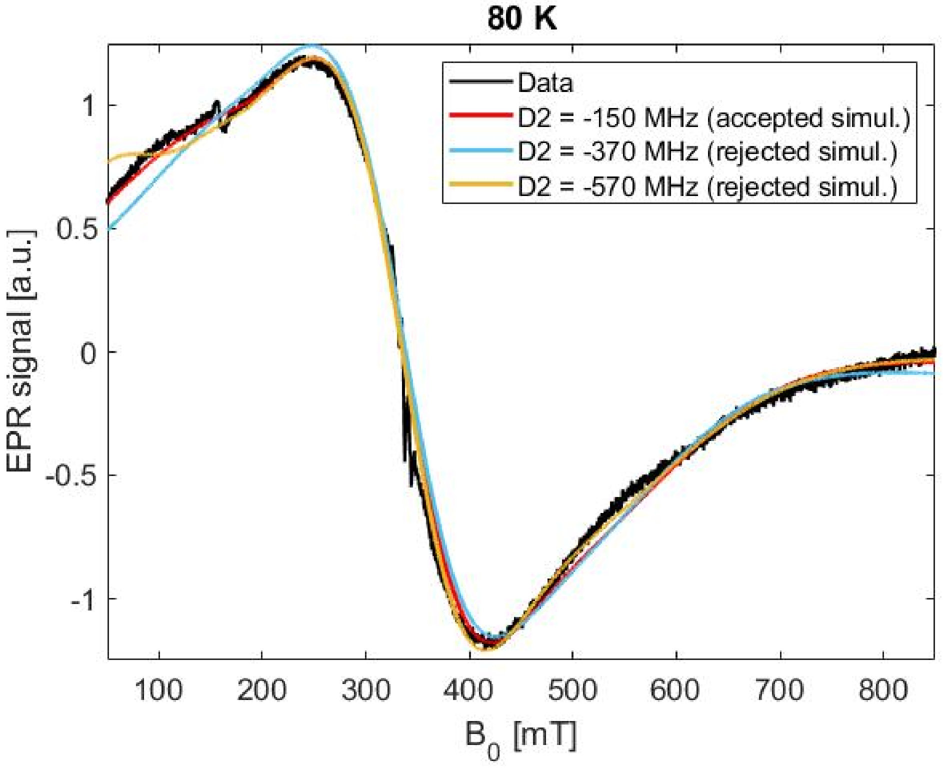

Figure S11: Simulations (colored lines) of EPR spectra (black lines) at 80 K performed by varying $D2$. An example of what is considered to be an acceptable simulation is depicted in red. Examples of rejected simulations are shown in light-blue and yellow.

Ferritin core and mononuclear Fe(III) EPR intensities

Mononuclear Fe(III) gives a characteristic EPR signal, with a sharp resonance at $g' = 4.3$, the so-called $g' = 4.3$ signal. This signal is also observed in the ferritin EPR spectra and the temperature dependence of its intensity is that of a paramagnetic species. Here we compare the amount of these species with respect to the ferritin core signal, the broad $g = 2$ signal. The EPR active spin concentration ratio of Fe(III) ($g' = 4.3$) and the ferritin core spin system can be determined from[2]

$$\frac{[\mathrm{Fe_m}]}{[\mathrm{core}]} = \frac{A_{\mathrm{Fe_m}} R_{\mathrm{Fe_m}} (\mathrm{Scan}_{\mathrm{Fe_m}})^2 G_{\mathrm{core}} \cdot (B_{\mathrm{m}})_{\mathrm{core}} \cdot (g_{\mathrm{core}})^2 S_{\mathrm{core}}(S_{\mathrm{core}}+1)}{A_{\mathrm{core}} R_{\mathrm{core}} \cdot (\mathrm{Scan}_{\mathrm{core}})^2 G_{\mathrm{Fe_m}} \cdot (B_{\mathrm{m}})_{\mathrm{Fe_m}} \cdot (g_{\mathrm{Fe_m}})^2 \cdot S_{\mathrm{Fe_m}}(S_{\mathrm{Fe_m}}+1)}, \tag{3}$$

where g is the g factor, S electron spin number of each species and A is the enclosed area (second integral) that is calculated from the blue (mononuclear Fe(III)) and red (for the core) curves shown in Fig. S12. 'Scan' is the sweep range, G is the relative gain of the signal amplifier, B_{m} is the modulation amplitude, R is a factor related to the hyperfine interactions, but for both, $\mathrm{Fe_m}$ and core, the hyperfine interactions are neglected. Scan, G, B_{m}, and R are the same for both $\mathrm{Fe_m}$ and core, so they cancel out. Thus, equation 3 becomes

$$\frac{[\mathrm{Fe_m}]}{[\mathrm{core}]} = \frac{A_{\mathrm{Fe_m}} \cdot (g_{\mathrm{core}})^2 \cdot S_{\mathrm{core}}(S_{\mathrm{core}}+1)}{A_{\mathrm{core}} \cdot (g_{\mathrm{Fe_m}})^2 \cdot S_{\mathrm{Fe_m}}(S_{\mathrm{Fe_m}}+1)}, \tag{4}$$

and follows

$$\frac{[\mathrm{Fe_m}]}{[\mathrm{core}]} = \frac{4135 \cdot (2.01539)^2 \cdot S_{\mathrm{core}}(S_{\mathrm{core}}+1)}{6463828 \cdot (1.9825)^2 \cdot \frac{5}{2}(\frac{5}{2}+1)} = 7.56 \cdot 10^{-5} \cdot S_{\mathrm{core}}(S_{\mathrm{core}}+1), \tag{5}$$

this expression is solely dependent on the total spin of the ferritin core signal. As an example, this ratio is 0.8 and 6.8 if the spin number S of the core is 100 and 300, respectively.

In order to compare the calculated EPR active spin concentration ratio $\frac{[\mathrm{Fe_m}]}{[\mathrm{core}]}$ to the number of paramagnetic Fe atoms, normalized by the number of superparamagnetic atoms, obtained by magnetometry ($N_{\mathrm{p}}/N_{\mathrm{Fh}}$) we need to multiply [core] with the number of iron atoms contributing to the ferrihydrite-like ferritin-core-EPR signal (N_{fh}). The amount of iron determined by ICP-MS (N_{ICP}) includes both mononuclear ($N_{\mathrm{Fe_m}}$) and ferritin-like iron ions (N_{fh}). However, the number of mononuclear Fe-ions that contribute to their EPR signal is small (2nd integral: 4575) compared to the ones contributing to the ferritin EPR signal (6778579). Therefore, we approximate the value obtained by ICP-MS to the number of iron atoms that contribute to the ferritin EPR signal, i.e., $N_{\mathrm{fh}} = 1967 \pm 78$.

The error of $\frac{[\mathrm{Fe_m}]}{[\mathrm{core}] \cdot N_{\mathrm{fh}}}$ was calculated using the following error propagation expression

$$\delta(F) = \frac{\Delta(F)}{|F|} \approx \frac{\Delta(M_1)}{|M_1|} + \frac{\Delta(M_2)}{|M_2|} + \frac{\Delta(M_3)}{|M_3|}, \tag{6}$$

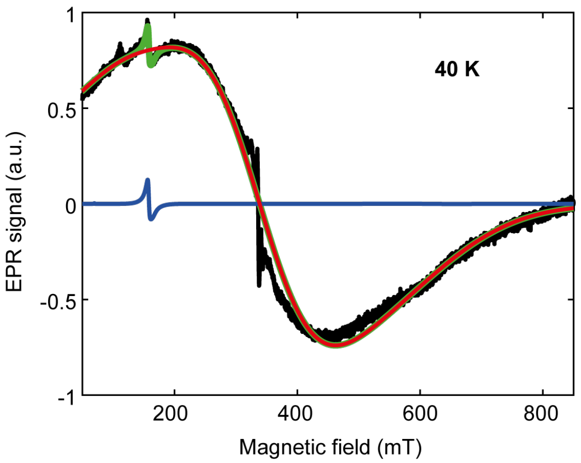

Figure S12: Simulations (colored lines) of EPR spectra (black line) at 40 K. In red, the simulation of the broad signal. In blue, the simulation of the rhombic mononuclear Fe(III) ion signal located at g'=4.3, using $S = 2.5$, $D = 20.96$ GHz, $E = 6.97$ GHz, $g = [1.9852, 2.0139, 1.9484]$, $g_{\mathrm{strain}} = [0.1328, 0.0242, 0.5009]$. In green, the superposition of both simulations.

where F is $\frac{[\mathrm{Fe_m}]}{[\mathrm{core}]\cdot N_{\mathrm{fh}}}$, M_1 is the second integral of the ferritin-core EPR signal, M_2 is the second integral of the mononuclear Fe EPR signal, M_3 is the number of iron atoms in the ferritin core N_{fh}. The errors of M_1 and M_2 were estimated by considering mainly the associated error of the simulations, using the Montecarlo error propagation with a small sampling number. Thus, δM_1 is 310 and δM_2 is 41. Finally, the error of M_3 is 78.

Table S2 shows the $\frac{[\mathrm{Fe_m}]}{[\mathrm{core}]\cdot N_{\mathrm{fh}}}$ estimated for both S_{core} of 100 and 300. The largest number ($3.6 \cdot 10^{-3}$) is obtained when considering $S_{\mathrm{core}} = 300$. This value is one order of magnitude smaller than the fraction $N_{\mathrm{p}}/N_{\mathrm{Fh}}$, found by magnetometry (0.0645). The relative errors in Table S2 round up to 10%. The difference in $\frac{[\mathrm{Fe_m}]}{[\mathrm{core}]\cdot N_{\mathrm{fh}}}$ for the two different S of a factor of 10 suggests that the uncertainty of $\frac{[\mathrm{Fe_m}]}{[\mathrm{core}]\cdot N_{\mathrm{fh}}}$ is the dominating source of uncertainty and could be in the range of an order of magnitude.

Table S2: EPR active spin concentration ratio per ferritin particle $\frac{[\mathrm{Fe_m}]}{[\mathrm{core}]\cdot N_{\mathrm{fh}}}$

S_{core}	$\frac{[\mathrm{Fe_m}]}{[\mathrm{core}]\cdot N_{\mathrm{fh}}}$	Abs. error	Rel. error (%)
100	$4 \cdot 10^{-4}$	$2 \cdot 10^{-5}$	5
300	$3.6 \cdot 10^{-3}$	$2 \cdot 10^{-4}$	6

Table S3: Data used in the error propagation calculation of $\frac{[\mathrm{Fe_m}]}{[\mathrm{core}]\cdot N_{\mathrm{fh}}}$

	M1 (a.u.)	M2 (a.u.)	M3 (iron ions)
Abs. value	6463828	4135	1967
Δ	310	41	78

EPR Model Simulations

In this section, some examples of the large set of spectra that constitute the EPR Model Simulations (80 K), are provided. The Model Simulations are used to constrain certain EPR parameters based on the trends seen. Figure S13 shows simulations of one EPR component with $S = 10$ by varying the D parameter from 100 to 5000 MHz and considering two different Gaussian broadening parameters (3000 and 8000 MHz). For the smallest broadening, D values larger than 500 MHz can not be used to account for the features observed in our experimental spectrum, this is shown in Fig. S13a where a sharp feature at low field is visible for the green curve (1000 MHz) and in Fig. S13c where the spectra become distorted. For the largest broadening, D cannot be greater than 1000 MHz (see Fig. S13d) since the spectrum starts to get progressively sharp at the lowest fields and flat at higher fields. In this way, to simulate the experimental spectrum measured at 80 K, figures S13a,b are used as starting points to inspect all parameters for D, H_{strain} and weights considering these two base components

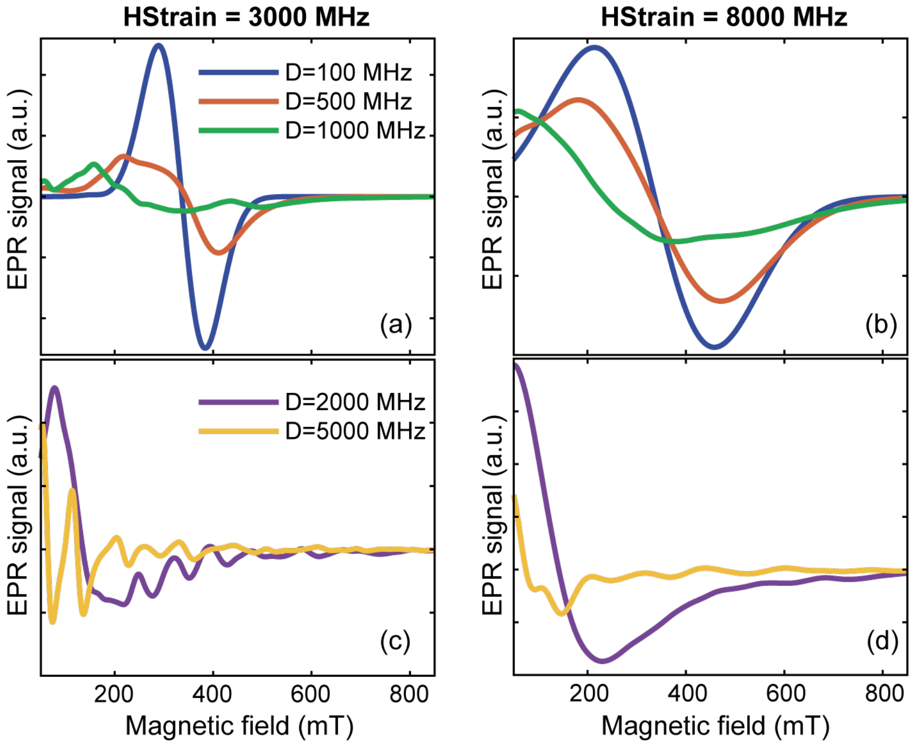

Figure S13: Model simulations (colored lines) performed at 80 K. The simulation parameters are $S = 10$, (a,c) $H_{\text{strain}} = 3000$ MHz and (b,d) $H_{\text{strain}} = 8000$ MHz. In blue, $D = 100$ MHz. In red, $D = 500$ MHz. In green, $D = 1000$ MHz. In purple, $D = 2000$ MHz. In yellow, $D = 5000$ MHz.

EPR alternative fitting approach

Following a different approach, a systematical fit of the spectra recorded at different temperatures was performed. First, We determined the S, D, and weight values of each component by fitting the EPR spectra recorded at 80 K (close to the middle-temperature range). The fitting was performed with a Matlab (R2019a) script, using the EasySpin package (5.2.4), using the scaling approach described in the main text.

The optimized scaled S and D values found for both EPR components, g and their respective weights are given in Table S4. Then, to fit the spectra from 20 to 190 K we used the scaled S, D and weight of each component determined at 80 K and only the Gaussian broadening given by an H_{strain} parameter was used as the fitting parameter for both components (see Fig. S16). The spectra between 5 and 15 K were omitted from the analysis because it was not possible to accurately discriminate the broad signal from the background noise.

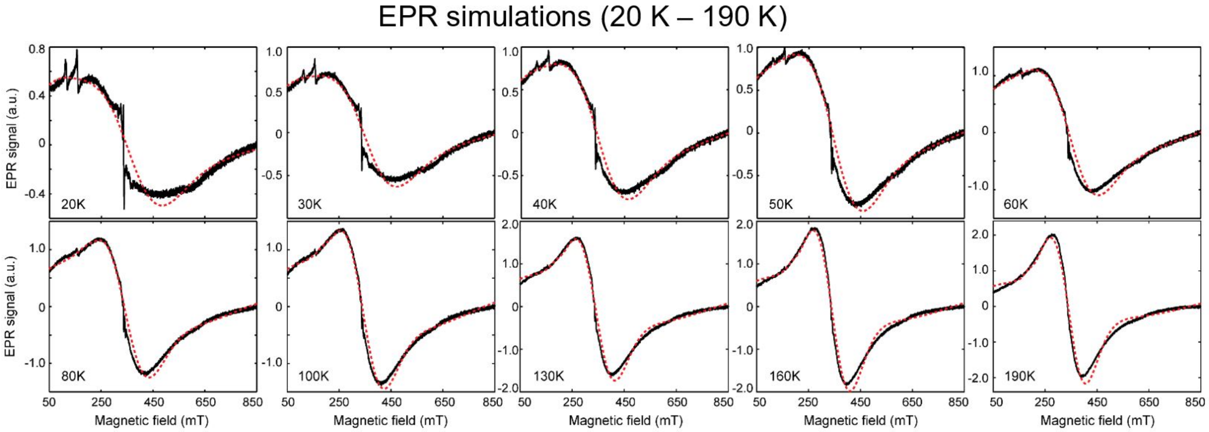

Figure S14: Simulations (red dash lines) of EPR spectra (black lines) using the 'alternative fitting model' (see text) for EPR spectra between 20 and 190 K. The simulations are performed with the automatic fitting routine with two spin systems. The system parameters are shown in Table S4. The scaled H_{strain} of both systems (fitting parameters) are depicted in figure S16.

The two individual components are shown in figure S15 at 20 K, 80 K, and 190 K. Component 2 (blue curve) is narrower and highly contributes to the total intensity of the spectra. Comparing the lineshape evolution at the different temperatures, it can be noticed that component 2 is responsible for the overall lineshape change. Whereas, component 1, being quite broad and lower in intensity, appears almost temperature independent. This is a sign of a poor fitting result since component 1 can be hiding many features. D parameter does not change the simulation shape. The H_{strain} changes the lineshape, but in an unsystematic way. In spite of the good agreement of simulations with the experiment, simulation parameters

do not give physical insight. Therefore, a different approach was used (see main text).

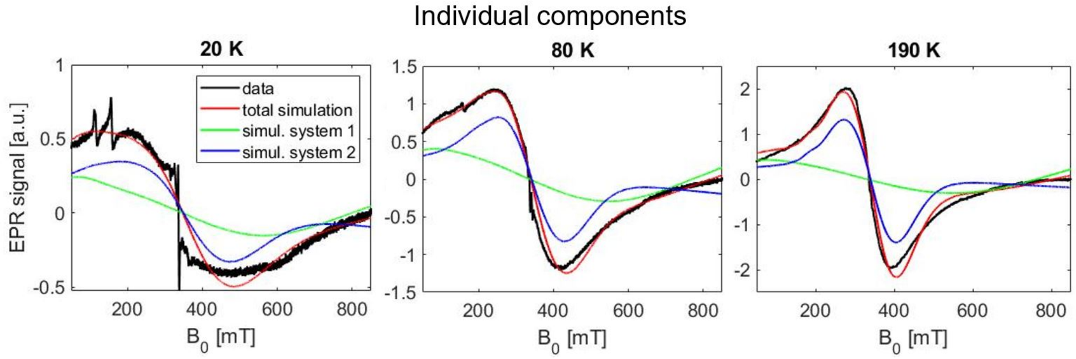

Figure S15: Total simulations (red lines) of EPR spectra (black lines) performed at 20 K, 80 K, and 190 K. The simulations are performed with two spin systems, as described above. The first system component is depicted in green. The second component is shown in blue.

Table S4: System parameters used for simulations of the EPR spectra. S is the Spin number and D the Zero-field axial component. The scaling factor is n = 14.6. The real parameters can be derived as follows: $D_{real} = D/n$, $S_{real} = S \cdot n$.

	S	g	D (MHz)	weight (%)
System 1	20	2.01	-167.2	36
System 2	7	2.01	-489.5	64

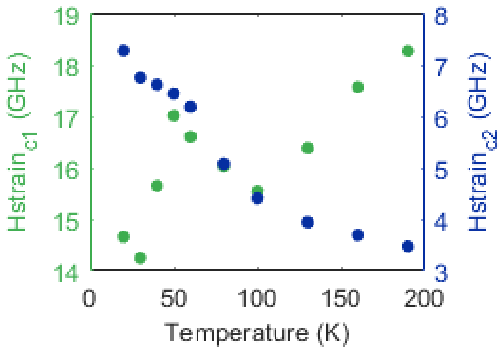

Figure S16: Broadening parameters (H_{strain}) from 'alternative fitting model' (see text) as a function of temperature, for component 1 (green dots) and component 2 (blue dots).

Other possible approaches to fitting EPR lineshapes and critical evaluation of the model used

Considering the quantum nature of small nano-particles, a quantum-mechanical model for the ferritin core is the method of choice. The Giant Spin model makes this computationally feasible and was shown to be valid also for large spin systems as the ferritin core.[3]

Before the Giant Spin model, several classical approaches were proposed.[1,4–7] These models are computationally less demanding, and, for example, distributions of magnetic parameters could be introduced more easily than in the Giant Spin model (see below). Usselman *et al.*[1] showed that the application of these models to the EPR spectra of magnetite nanoparticles lead to inconsistencies, either resulting in unphysical magnetic parameters or did not fit the lineshape in the low-temperature regime, making quantum mechanical models the more attractive choice.

To implement the distribution of magnetic parameters, suggested by the analysis of the magnetometry data, in the Giant Spin model, however, is not straightforward. In the present study, we use two EPR components, and a significant Gaussian broadening of each of these components to represent the distribution. This approach leads to good simulations of the experimental spectra, and, as shown in Table 2 (main text), overall the parameters derived from of this interpretation agree well with the magnetometry data. Yet, it cannot be excluded that other representations of magnetic properties could also work, given the broadness of the experimental spectra.

Equilibrium magnetization models

The evaluation of eq. 4 requires numerical integration. Standard numerical integration routines require excessive computation time to evaluate the five-fold integral, while Monte Carlo integration does not work well, due to the different evaluation points at the numerator and denominator. Therefore, integrals have been replaced with sums over discrete states for a finite number of particles, that is

$$M(B) = \frac{\displaystyle\sum_{\phi = j\,\delta\phi} \sin\phi \, \frac{\displaystyle\sum_{\varepsilon = i\,\delta\varepsilon} \sum_{\lambda = l\,\delta\lambda} \sum_{\psi = k\,\delta\psi} \sum_{\theta = n\,\delta\theta} \zeta\, e^{-E/k_{\mathrm{B}}T} \sin\theta}{\displaystyle\sum_{\varepsilon = i\,\delta\varepsilon} \sum_{\lambda = l\,\delta\lambda} \sum_{\psi = k\,\delta\psi} \sum_{\theta = n\,\delta\theta} e^{-E/k_{\mathrm{B}}T}}}{\displaystyle\sum_{\phi = j\,\delta\phi} \sin\phi} \tag{7}$$

where $\delta\phi = \delta\lambda = \delta\psi = \delta\theta = \pi/2N$ are exact dividers of $\pi/2$ and $\delta\varepsilon$ an exact divider of the maximum canting angle $\varepsilon_{\max}$. The maximum canting angle is defined as the mean root square of ϵ weighted by $e^{-E/k_{\mathrm{B}}T}$ and avoids the evaluation of strongly canted states with negligible occurrence. As ϵ increases with the applied field, $\varepsilon_{\max}$ is evaluated iteratively starting for a series of field values starting from $\varepsilon_{\max} = \pi B/2B_E$. The number $N+1$ of samples in each dimension is chosen to be the largest possible for given memory and time constraints. The numerical procedure has been implemented in Wolfram Mathematica and compiled in C using the built-in compiler. A PC with 64 GB RAM supports calculations up to $N = 27$ for a total of 10^9 combinations of particle orientations and magnetic states, for an angular resolution of $\sim 3.3^\circ$ in ϕ, λ, ψ, and θ.

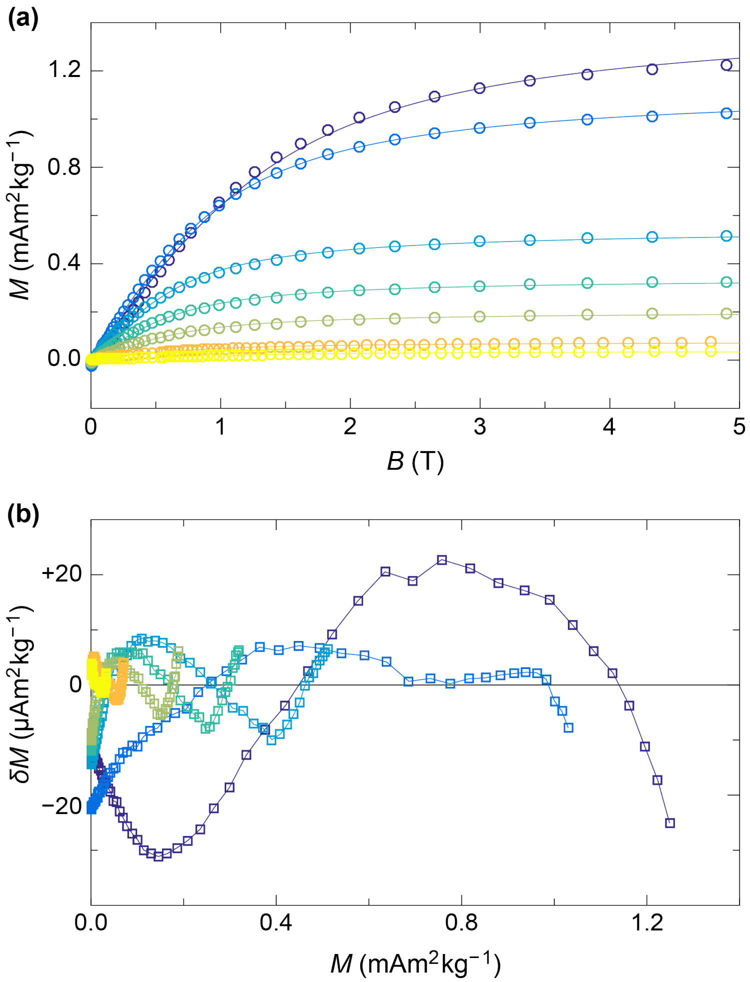

Figure S17: (a) Initial magnetization curves measured at temperatures comprised between 3 K (blue circles) and 200 K (yellow circles), and global fit to the modified Langevin model with one superparamagnetic component without moment distribution. (b) Fit residuals as a function of the predicted magnetization.

.

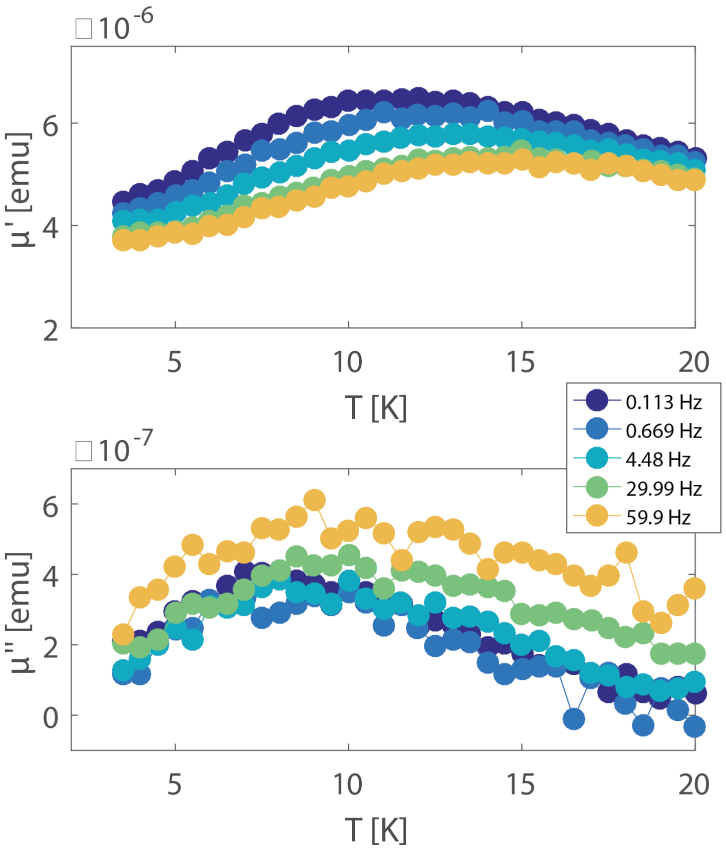

Figure S18: Real μ' and Imaginary μ'' parts of the magnetic moment of the human-liver ferritin (raw data). The legend refers to the frequency of the oscillating B_1 field.

.

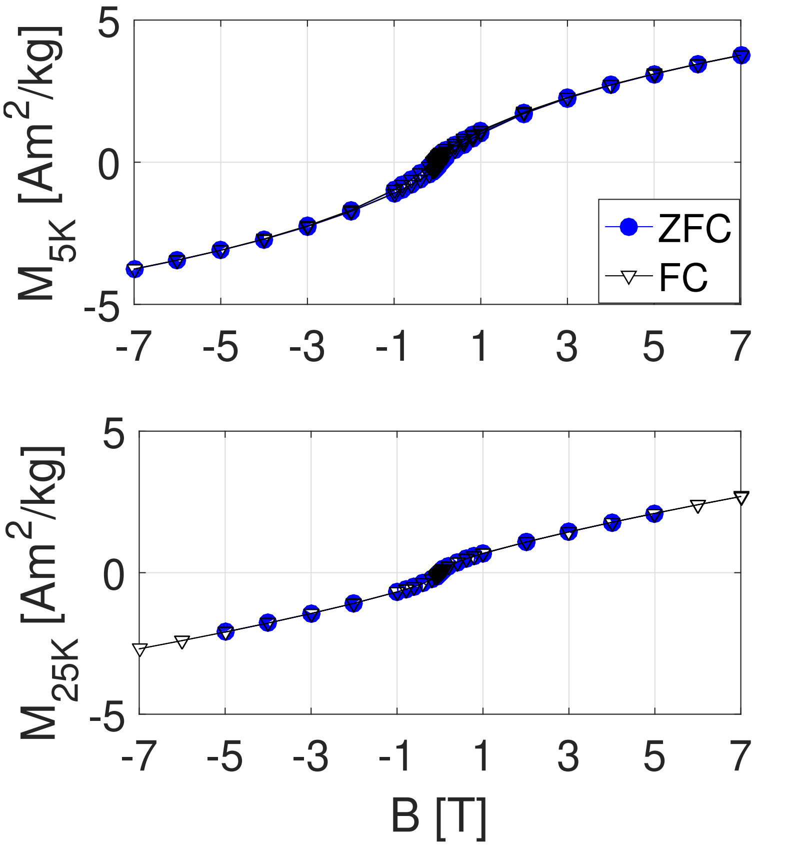

Figure S19: Full hysteresis acquired at 5 K and 25 K both in ZFC and FC conditions, to determine the exchange bias of human-liver ferritin.

.

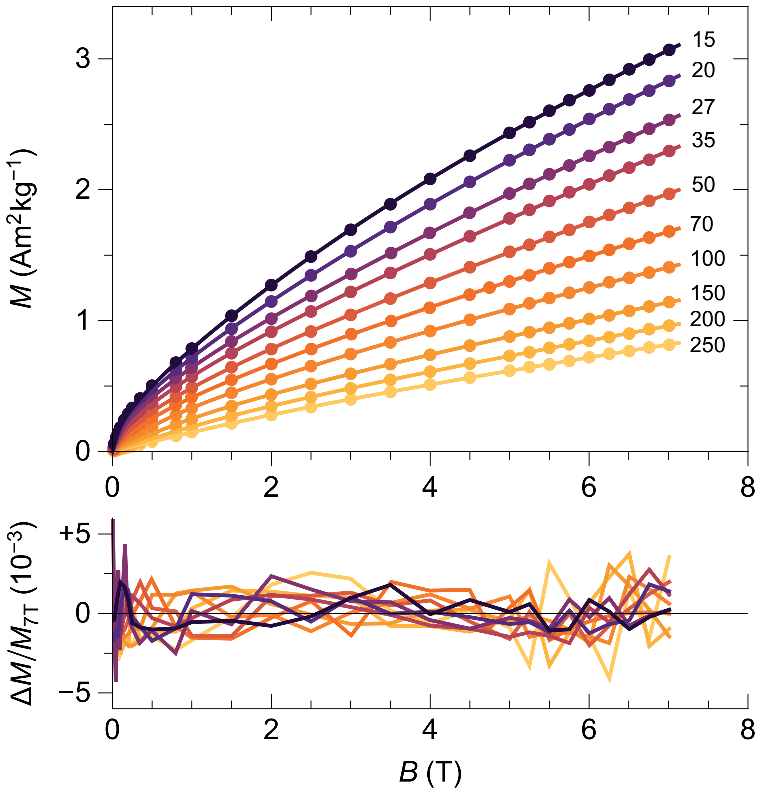

Figure S20: Measured isothermal magnetization curves (dots) and corresponding best fits with a two-component Langevin law obtained from eq. 12 after replacing the magnetic moment distribution with two single-valued moments (lines), at temperatures indicated by numbers. Residuals, defined as the difference between measurements and model, normalized by the measured magnetization at 7 T, are plotted below.

.

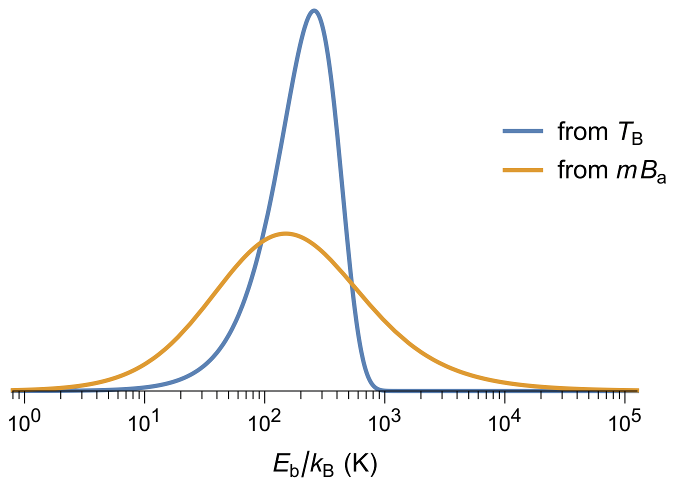

Figure S21: Comparison between a direct estimate of the energy barrier distribution, obtained from the blocking temperature distribution of the IRM Component 1, and the energy barrier distribution expected from $E_{\mathrm{b}} = mB_{\mathrm{a}}/2$ with the incorrect assumption that m and B_{a} are independent variables.

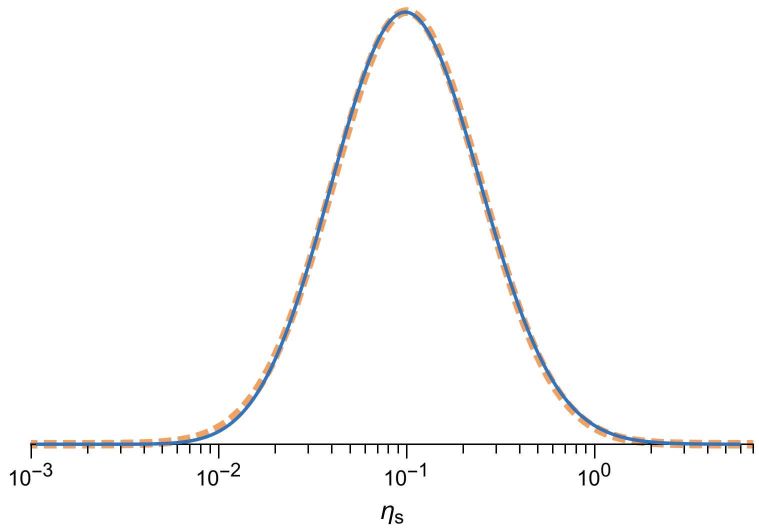

Figure S22: Reconstructed distribution of η_s obtained from the deconvolution of the magnetic moment and volume distributions (solid line), and best-fit normal distribution $N(\log \eta_s, -1, 0.392)$.

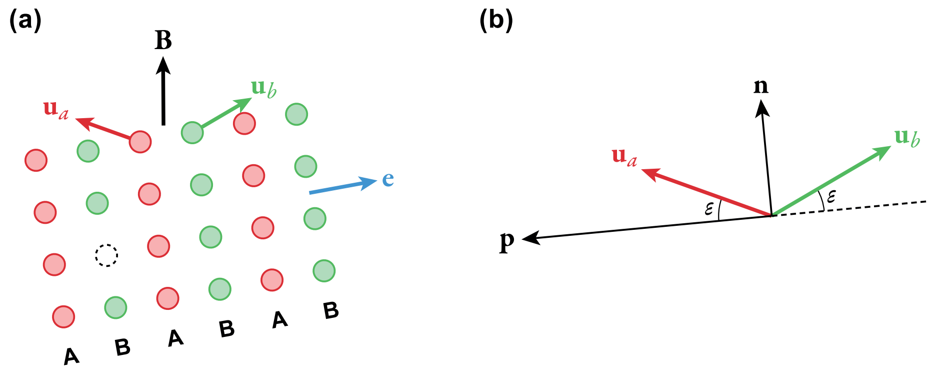

Figure S23: Vector conventions for the AF lattice model used in Section 3.2 main text. (a) Sublattices A and B, and corresponding lattice magnetization vectors $\mathbf{u}_a$ and $\mathbf{u}_b$, respectively. $\mathbf{B}$ is the applied field and $\mathbf{e}$ the unit vector pointing to the easy axis direction for uniaxial anisotropy. The dashed circle represents a defect in sublattice B. (b) Definition of the auxiliary vectors $\mathbf{p}$ and $\mathbf{n}$, which define the canting angle ϵ.

Spontaneous spin canting

Spontaneous canting is generated by an additional energy term proportional to $\mathbf{c} \cdot (\mathbf{u}_a \times \mathbf{u}_b)$, with $\mathbf{c}$ being the unit vector perpendicular to the canting plane. In this case, the total magnetic energy per unit of volume becomes

$$\begin{aligned} \frac{E}{M_0} = \quad & B_{\mathrm{E}}\mathbf{u}_a \cdot \mathbf{u}_b + B_{\mathrm{E}} \tan(2\varepsilon_{\mathrm{s}})\, \mathbf{c} \cdot (\mathbf{u}_a \times \mathbf{u}_b) - \mathbf{B}(\mathbf{u}_a + \mathbf{u}_b) \\ & + E_{\mathrm{a}}(\mathbf{u}_a, \mathbf{u}_b, \mathbf{e}), \end{aligned} \tag{8}$$

where the term E_{a} accounts for the combined effect of surface and volume anisotropy. In the isotropic case ($E_{\mathrm{a}} = 0$), the total energy is insensitive to the direction $\mathbf{n}$ of canting, as long as it is perpendicular to $\mathbf{c}$. Therefore, $E_{\mathrm{a}} = 0$ describes an azimuthal dependence of E with respect to $\mathbf{c}$, which requires an additional degree of freedom with respect to the case of a defect moment. Given the minor role played by realistic values of the magnetocrystalline anisotropy in the defect moment simulations of Fig. 4, numerical calculations have been performed with $E_{\mathrm{a}} = 0$.

List of Symbols and Acronyms

B	Applied magnetic field. Also $\mathbf{B}$.
B_0	Resonance field in EPR spectra.
B_{EX}	Exchange field.
B_{a}	Anisotropy field.
B_{esf}	Equilibrium spin-flop field.
B_{ex}	Exchange coupling field.
B_{sf}	Spin-flop field.
D	Zero-field splitting.
E_{b}	Anisotropy energy.
G_{pp}	Amplitude peak to peak of field modulation.
H_{strain}	Gaussian broadening.
J	Exchange constant.
K	Anisotropy constant.
K_{S}	Surface anisotropy.
K_{V}	Uniaxial volume anisotropy.
M_0	Sublattice magnetization.
M_{τ}	Remanent magnetization.
M_{s}	Saturation magnetization.
N_{A}	Avogadro constant.
N_{s}	Surface spins.
S	Total spin of the particle.
T	Temperature.
T_{b}	Blocking temperature.
V	Particle volume.
$\chi_{\perp}$	Bulk perpendicular susceptibility.
χ_{nc}	low-field susceptibility.
$\mathbf{D}$	Traceless zero-field splitting tensor.
$\mathbf{S}$	Electron spin operator.
$\mathbf{g}$	g tensor.
μ	(1) Magnetic moment in EPR. (2) Mean value of distributions.
μ_{B}	Bohr magneton.

ε_{s}	Zero-field-canting angle.
c	Concentration of defects.
g'	Effective g.
k_{B}	Boltzmann constant.
m	Magnetic moment in magnetometry.
m_{c}	Canting moment.
m_{uc}	Uncompensated moment in magnetometry.
**AF**	Antiferromagnetic.
**E1**	EPR simulation component 1.
**E2**	EPR simulation component 2.
**EELS**	Electron Energy-Loss Spectroscopy.
**EMR**	Electron magnetic resonance.
**EPR**	Electron paramagnetic resonance.
**FC**	Field-cooled.
**Fh**	Ferrihydrite.
**HuLiFt**	Human-liver ferritin.
**ICP-MS**	Inductively coupled plasma mass spectrometry.
**IRM**	Isothermal remanent magnetization.
**MRI**	Magnetic resonance imaging.
**NMR**	Nuclear magnetic resonance.
**SAXS**	Small-Angle X-ray Scattering.
**SDS-PAGE**	Sodium dodecyl sulfate–polyacrylamide gel electrophoresis.
**SNR**	Signal-to-noise ratio.
**TEM**	Transmission electron microscopy.
**XANES**	X-ray adsorption near edge spectroscopy.
**ZFC**	Zero-Field-cooled.

References

(1) Usselman, R. J.; Russek, S. E.; Klem, M. T.; Allen, M. A.; Douglas, T.; Young, M.; Idzerda, Y. U.; Singel, D. J. Temperature dependence of electron magnetic resonance spectra of iron oxide nanoparticles mineralized in Listeria innocua protein cages. *Journal of Applied Physics* **2012**, *112*, 084701.

(2) Weil, J. A.; Bolton, J. R. *Electron Paramagnetic Resonance. Elementary Theory and Practical Applications*; John Wiley and Sons, Inc., Hoboken, New Jersey, 2007; p 546.

(3) Fittipaldi, M.; Mercatelli, R.; Sottini, S.; Ceci, P.; Falvo, E.; Gatteschi, D. Sensing the quantum behaviour of magnetic nanoparticles by electron magnetic resonance. *Phys. Chem. Chem. Phys.* **2016**, *18*, 3591–3597.

(4) Noginova, N.; Chen, F.; Weaver, T.; Giannelis, E. P.; Bourlinos, A. B.; Atsarkin, V. A. Magnetic resonance in nanoparticles: between ferro- and paramagnetism. *Journal of Physics: Condensed Matter* **2007**, *19*, 246208.

(5) Berger, R.; Kliava, J.; Bissey, J.-C.; Baïetto, V. Magnetic resonance of superparamagnetic iron-containing nanoparticles in annealed glass. *Journal of Applied Physics* **2000**, *87*, 7389–7396.

(6) Berger, R.; Bissey, J.-C.; Kliava, J.; Daubric, H.; Estournès, C. Temperature dependence of superparamagnetic resonance of iron oxide nanoparticles. *Journal of Magnetism and Magnetic Materials* **2001**, *234*, 535–544.

(7) Usselman, R. J.; Klem, M. T.; Allen, M.; Walter, E. D.; Gilmore, K.; Douglas, T.; Young, M.; Idzerda, Y.; Singel, D. J. Electron magnetic resonance of iron oxide nanoparticles mineralized in protein cages. *Journal of Applied Physics* **2005**, *97*, 10M523.
